# An efficient Bayesian meta-analysis approach for studying cross-phenotype genetic associations

**DOI:** 10.1371/journal.pgen.1007139

**Published:** 2018-02-12

**Authors:** Arunabha Majumdar, Tanushree Haldar, Sourabh Bhattacharya, John S. Witte

**Affiliations:** 1 Department of Epidemiology and Biostatistics, University of California, San Francisco, California, United States of America; 2 Institute for Human Genetics, University of California, San Francisco, California, United States of America; 3 Interdisciplinary Statistical Research Unit, Indian Statistical Institute, Kolkata, India; Emory University, UNITED STATES

## Abstract

Simultaneous analysis of genetic associations with multiple phenotypes may reveal shared genetic susceptibility across traits (pleiotropy). For a locus exhibiting overall pleiotropy, it is important to identify which specific traits underlie this association. We propose a Bayesian meta-analysis approach (termed CPBayes) that uses summary-level data across multiple phenotypes to simultaneously measure the evidence of aggregate-level pleiotropic association and estimate an optimal subset of traits associated with the risk locus. This method uses a unified Bayesian statistical framework based on a spike and slab prior. CPBayes performs a fully Bayesian analysis by employing the Markov Chain Monte Carlo (MCMC) technique Gibbs sampling. It takes into account heterogeneity in the size and direction of the genetic effects across traits. It can be applied to both cohort data and separate studies of multiple traits having overlapping or non-overlapping subjects. Simulations show that CPBayes can produce higher accuracy in the selection of associated traits underlying a pleiotropic signal than the subset-based meta-analysis ASSET. We used CPBayes to undertake a genome-wide pleiotropic association study of 22 traits in the large Kaiser GERA cohort and detected six independent pleiotropic loci associated with at least two phenotypes. This includes a locus at chromosomal region 1q24.2 which exhibits an association simultaneously with the risk of five different diseases: Dermatophytosis, Hemorrhoids, Iron Deficiency, Osteoporosis and Peripheral Vascular Disease. We provide an R-package ‘CPBayes’ implementing the proposed method.

## Author summary

Genome-wide association studies (GWAS) have detected shared genetic susceptibility to various human diseases (pleiotropy). We propose a Bayesian meta-analysis method CPBayes that simultaneously evaluates the evidence of overall pleiotropy while determining which traits are pleiotropic. This approach investigates pleiotropy using GWAS summary statistics and allows for overlapping subjects across traits. It performs a fully Bayesian analysis and offers a flexible inference. CPBayes also provides additional information about a pleiotropic signal, such as the trait-specific posterior probability of association and the credible interval of unknown true genetic effects. Using computer simulations and an application to a large GWAS cohort, we demonstrate that CPBayes can offer improved accuracy compared to the existing subset-based meta-analysis approach ASSET. We provide a user-friendly R-package ‘CPBayes’ for general use of this approach.

## Introduction

Genome-wide association studies (GWAS) have detected loci associated with multiple different traits and diseases (i.e., pleiotropy) [1]. For example, pleiotropy has been observed for different types of cancers [2], immune-mediated diseases [3] and psychiatric disorders [4]. As another example, Pickrell et al. [5] systematically compared the genetic architecture of 42 phenotypes and reported substantial pleiotropy. Analyzing pleiotropy may provide a better understanding of shared pathways and biological mechanisms common to multiple different diseases/phenotypes. From the perspective of clinical genetics, the discovery of a locus simultaneously associated with multiple diseases can support the use of a common therapeutic intervention.

When evaluating a group of phenotypes, only a subset of them may exhibit pleiotropy. For example, the Global Lipids Genetics Consortium [6] discovered novel pleiotropic loci associated with different subsets of blood lipid traits. In particular, variants in the genes *RSPO3*, *FTO*, *VEGFA*, *PEPD* were associated with HDL and triglycerides, but not with LDL or total cholesterol. Hence, in addition to evaluating the evidence of overall pleiotropic association, it is crucial to determine which traits are associated with the risk locus to better interpret the pleiotropic signal. Another important consideration is the availability of individual level data from multiple GWAS of different phenotypes. When accessing individual level data is difficult, one can use recently developed methods to investigate pleiotropy using more readily available genome-wide (GW) summary statistics [7–12].

Pleiotropy can be detected in two main ways—a single nucleotide polymorphism (SNP) that is associated with multiple traits, or a genomic region that is associated with multiple traits. For example, Bhattacharjee et al. [7] proposed the subset-based meta-analysis approach ASSET focussing on SNP-level pleiotropy and Giambartolomei et al. [11] introduced a Bayesian approach to explore whether two association signals in the same genomic region obtained from two different GWAS share a single causal variant or multiple causal variants.

In addition, there have been other recent works on methods development and applications for pleiotropy. Andreassen et al. [8] proposed a conditional false discovery rate (FDR) approach, and detected novel loci associated with Schizophrenia by leveraging information on the genetic pleiotropy between Schizophrenia and cardiovascular risk factors. Andreassen et al. [9] applied the same approach to study the shared genetic architecture underlying Schizophrenia and Bipolar disorder. Liley and Wallace [12] modified the conditional FDR method to allow for shared controls between two GWAS. Chung et al. [10] proposed a statistical method GPA to prioritize GWAS signals by incorporating pleiotropy and annotation information. They also demonstrated that GPA performs better than the conditional FDR approach with respect to accurately prioritizing risk SNPs. However, these methods were mainly developed to only analyze a pair of traits at a time. In contrast, ASSET is more directly suited for evaluating pleiotropy simultaneously across two or more traits. It provides a p-value evaluating the evidence of aggregate-level pleiotropic association and an optimal subset of associated/non-null traits. Recent studies [13–17] have used ASSET as a primary tool for pleiotropy analysis.

In this article, we focus on SNP-level pleiotropy and propose a Bayesian meta-analysis approach CPBayes (Cross-Phenotype Bayes) that simultaneously provides a measure of the evidence of aggregate-level pleiotropic association and an optimal subset of associated traits underlying a pleiotropic signal. The evidence of pleiotropy is measured by the local false discovery rate (locFDR) and with Bayes factor (BF). CPBayes explicitly takes into account correlation between summary statistics. CPBayes considers heterogeneity both in the size and direction of genetic effects across phenotypes. It also estimates the posterior probability of each phenotype being associated with the risk locus that quantifies the relative contribution of the traits underlying a pleiotropic signal. It is explicitly designed to simultaneously analyze two or more phenotypes.

The Bayesian framework of CPBayes is based on a spike and slab prior, which is commonly used due to its appropriateness and simplicity in solving two-class classification problems [18–22]. The application of the spike and slab prior in genetic association studies is gradually increasing [23–25]. With a spike and slab prior, the spike element represents a null effect, and the slab component represents a non-null effect. The spike part can be either a positive mass at zero (Dirac spike [18]) or a normal distribution with mean zero and a small variance (continuous spike [19]). We design the Gibbs samplers for these two type of prior spikes for both uncorrelated and correlated summary statistics across traits. The continuous spike and slab prior can alternatively be viewed as a special case of the scale mixture of two normal distributions [26, 27]. Such scale mixtures have been employed to estimate the effect size distributions and replication probabilities in GWAS [28, 29]. We demonstrate by simulations that the continuous spike offers better accuracy in the selection of associated traits than the Dirac spike (also observed by George and McCulloch [19] in general context). The Gibbs sampling for the former is also computationally much faster than that for the latter due to simpler analytic expressions of the full conditional posterior distributions of the model parameters. Hence, we adopted the continuous spike for constructing CPBayes.

We compare CPBayes with ASSET in various simulation scenarios. While selecting the non-null traits underlying a pleiotropic signal, we also compared it with the standard Benjamini-Hochberg (BH) FDR controlling procedure with the level of FDR equal to 0.01 (BH_0.01_) [30]. The choice of the FDR level is guided by Majumdar et al. [31] who demonstrated that the simple BH procedure provides better selection accuracy than various different approaches. CPBayes resembles ASSET in that both methods simultaneously draw inference on the evidence of aggregate-level pleiotropic association and on the optimal subset of non-null traits. But, the key advantage of CPBayes is that it selects the non-null traits with substantially higher specificity (proportion of null traits discarded from the optimal subset) than ASSET while maintaining a good level of sensitivity (proportion of non-null traits included in the subset). We also compared CPBayes with GPA for a pair of traits using simulations.

We contrast CPBayes and ASSET in the analysis of 22 phenotypes in the large Kaiser “Resource for Genetic Epidemiology Research on Adult Health and Aging” (GERA) cohort [dbGaP Study Accession: phs000674.v1.p1]. CPBayes identified six independent pleiotropic loci associated with at least two phenotypes including a locus at chromosomal region 1q24.2 that exhibits an association with five different diseases: Dermatophytosis, Hemorrhoids, Iron Deficiency, Osteoporosis and Peripheral Vascular Disease. ASSET identified a larger number of independent pleiotropic loci associated with more than one trait, but selected many phenotypes with very weak genetic effects. We provide an R-package ‘CPBayes’ implementing the proposed method for a general use.

### Overview of methods

Given the summary statistics for a SNP across multiple traits, CPBayes estimates two different measures evaluating overall pleiotropic association and an optimal subset of associated traits underlying a pleiotropic signal. The evidence for aggregate-level pleiotropic association is given by the local false discovery rate (locFDR) and the Bayes factor (BF). Let *H*_0_ denote the global null hypothesis of no association with any trait and *H*_1_ denote the global alternative hypothesis of overall association with at least one of the traits. Then locFDR is defined as the posterior probability of *H*_0_ being true conditioned on the summary statistics and BF is the ratio of the likelihood of summary statistics under *H*_1_ versus *H*_0_. A small value of locFDR (e.g., 0.05) or a large value of Bayes factor (e.g., BF > 1) indicates an evidence for the overall pleiotropic association. We estimate the locFDR and BF based on the MCMC posterior sample of model parameters obtained by Gibbs sampling. The subset of traits which is selected as the set of non-null traits most often in the MCMC posterior sample is defined as the maximum a posteriori (MAP) estimate of the optimal subset of associated traits.

CPBayes explicitly accounts for possible correlation between the effect estimates across traits. To estimate the correlation structure of the effect estimates, we use two different approaches—one based on the number of overlapping cases and controls between studies and another based on genome-wide summary statistics across traits. The correlation formulae based on the number of shared cases and controls (Eq 6 in Material and methods) is accurate when the genetic variant and environmental covariates are not associated with the traits of interest. However in real data, environmental covariates are expected to be associated with the primary traits. In such scenario, the GW summary statistics based approach is more robust with respect to estimating a reasonably accurate correlation structure.

CPBayes also provides some more insights into a pleiotropic signal, e.g., marginal trait-specific posterior probability of association (PPA_*j*_), direction of associations, credible interval of true genetic effects, etc. Detailed description of CPBayes and a brief outline of ASSET, BH_0.01_ and GPA are provided in the “Material and methods” section and supporting information.

### Simulation study

We compare CPBayes and ASSET with respect to correctly detecting a signal of pleiotropy and the accuracy of selection of non-null traits underlying a pleiotropic signal in various simulation scenarios. We consider multiple case-control studies with or without shared controls [7] and a cohort study where the data on multiple disease states are available for a group of individuals [31, 32]. First, we specify the simulation model to generate the phenotype and genotype data. After computing the summary statistics based on the simulated data, we assume that only the summary-level data are available. For case-control studies with overlapping subjects or a cohort study, we estimate the correlation structure of summary statistics based on Eq 6 (Material and methods).

For non-overlapping case-control studies, we consider a separate group of 7000 cases and 10000 controls in each study. For overlapping case-control studies, we consider a distinct set of 7000 cases in each study, and a common set of 10000 controls shared across all the studies. For each disease, we assume an overall disease prevalence of 10% in the whole population. While simulating the genotype data for multiple case-control studies, we assume the standard logistic model of disease probability conditioning on the genotype: $\text{P}\left(\text{case}|G\right)=\frac{\text{exp}(\alpha +\beta G)}{1+\text{exp}(\alpha +\beta G)}$, where *G* is the genotype at the SNP of interest coded as the minor allele count (0, 1, 2). We assume that the SNP is in Hardy-Weinberg equilibrium (HWE). Let *A* (minor) and *a* be the two alleles at the SNP and *p* = *P*(*A*). Under HWE, the genotype probabilities are: P(*AA*) = *p*^2^, P(*Aa*) = 2*p*(1 − *p*), P(*aa*) = (1 − *p*)^2^. Given the log odds ratio (*β*) and disease prevalence, we compute the probability of observing each genotype conditioned on the case/control status using Bayes theorem. Based on these conditional probabilities, we simulate the genotypes in cases and controls.

For the cohort study, we consider 15000 individuals. First, we generate the genotype data at a quantitative trait locus (QTL) and continuous multivariate phenotype data using the simulation model in Majumdar et al. [31] that was adopted from Galesloot et al. [32]. Then we dichotomize each continuous phenotype (liability) to case-control status subject to an overall disease prevalence of 10%. We describe the simulation model in more details in S1 Text in supporting information.

We emphasize that the simulation models used here are general in nature and independent of the modeling assumptions underlying CPBayes which are also very general and only require that the sample sizes of the participating GWAS should be sufficiently large to satisfy the standard asymptotic properties (Material and methods). These simulation models were also used in Bhattacharjee et al. [7], Galesloot et al. [32], Majumdar et al. [31].

For overlapping case-control studies and a cohort study when the summary statistics are expected to be correlated, a combined strategy of CPBayes is implemented (Material and methods). If the effect estimates are strongly correlated and a majority of the traits are associated with the risk locus (non-sparse scenario), the Gibbs sampler underlying CPBayes may sometimes be trapped in a local mode of the posterior distribution of model parameters. To increase robustness for correlated summary statistics, CPBayes considers a joint strategy combining its uncorrelated and correlated versions. First, we implement CPBayes considering the correlation structure of the effect estimates. If the selected subset of non-null traits have the smallest univariate association p-values among all the traits, we accept the results; otherwise, we employ CPBayes assuming that the effect estimates are uncorrelated and accept the results obtained.

#### Evaluation of aggregate-level pleiotropic association

First we evaluate the methods in the context of detecting overall pleiotropic association. Consider 1000 SNPs of which 2% (20) are risk SNPs and 98% are null SNPs (not associated with any trait). The minor allele frequency (MAF) at all SNPs are randomly simulated from Uniform(0.05,0.5) distribution. One or more traits are associated with a risk SNP. Let *K*_1_ denote the number of associated phenotypes among a total of *K* phenotypes. We consider *K* = 5, 10, 15; *K*_1_ = 0, 1, 2, 3 when *K* = 5, *K*_1_ = 0, 2, 4, 6 when *K* = 10, *K*_1_ = 0, 3, 6, 9 when *K* = 15.

For *K* = 5, out of 20 risk SNPs, 8, 6 and 6 SNPs are associated with 1, 2 and 3 traits, respectively. For *K* = 10; 8, 6 and 6 risk SNPs are associated with 2, 4 and 6 traits, respectively. Similarly for *K* = 15; 8, 6 and 6 risk SNPs are associated with 3, 6 and 9 traits, respectively. For *K* = 5, first, *K*_1_ is set as 1 for 8 risk SNPs randomly selected from 20 risk SNPs without replacement; next, *K*_1_ is set as 2 for 6 risk SNPs randomly sampled from the remaining 12 risk SNPs. The same strategy is employed for *K* = 10 and 15. Corresponding to a risk SNP, each non-null trait is considered to be either positively or negatively associated with equal probability. The odds ratio (OR) for a non-null trait is randomly simulated from Uniform(1.05, 1.25) (Uniform(1/1.25, 1/1.05)) if the trait is positively (negatively) associated.

Note that the locFDR and Bayes factor are not comparable to the p-value. Hence we compared the number of risk SNPs identified as being associated (true discoveries) while adjusting the threshold of CPBayes locFDR and ASSET p-value such that both methods detect the same number of null SNPs as being associated (false discoveries). This can be viewed as a partial receiver operating characteristic (ROC) curve. The method with a greater area under the curve (AUC) is preferred. Coram et al. [33] considered a similar strategy to compare different methods that provide non-comparable measures of testing association. They also suggested an alternative strategy to successively reject SNPs with increasing locFDR until the average locFDR of the rejected set (estimated FDR) reaches a pre-fixed threshold. We implement this procedure for CPBayes to detect pleiotropic signals at a pre-specified level of FDR. We apply the Benjamini-Hochberg FDR controlling procedure (with the same FDR level as for CPBayes) to ASSET p-values and compare the detected number of true and false positives with that obtained by CPBayes (Table 1).

**Table 1:** Simulation results: The number of true positives and false positives detected by CPBayes and ASSET at different significance levels of FDR.

Study design	*K*	Method	Significance level of FDR								
			0.01			0.05			0.1		
			nTP	nFP	rFDR	nTP	nFP	rFDR	nTP	nFP	rFDR
Non-overlapping case-control studies	5	CPB	16.1	0	0	17.2	0.7	0.04	17.5	2.3	0.11
		AST	16.0	0	0	16.8	1.0	0.05	17.5	17.5	0.47
	10	CPB	18.7	0	0	19.3	0.5	0.02	19.5	1.3	0.06
		AST	18.8	0	0	18.9	0	0	19.0	0	0
	15	CPB	19.0	0	0	19.4	0.6	0.03	19.6	1.6	0.08
		AST	19.1	0	0	19.3	0	0	19.4	0	0
Overlapping case-control studies	5	CPB	16.1	0	0	17.3	0	0	18.3	0.3	0.01
		AST	14.2	0	0	15.2	0	0	15.7	0	0
	10	CPB	18.9	0	0	19.4	0.3	0.02	19.7	1.3	0.06
		AST	16.5	0	0	17.2	0	0	17.5	0	0
	15	CPB	19.2	0	0	19.7	0.4	0.02	20.0	1.3	0.06
		AST	17.0	0	0	17.7	0	0	18.0	3.5	0.13
Cohort study	5	CPB	15.5	0.16	0.01	17.2	1.5	0.08	17.8	3.1	0.15
		AST	12.5	0	0	14.3	0	0	15.3	0.04	0
	10	CPB	19.2	0	0	19.8	0.6	0.03	19.9	1.7	0.08
		AST	16.8	0	0	17.8	0.1	0.01	18.4	1.5	0.07
	15	CPB	19.7	0.02	0.001	19.9	0.9	0.04	19.9	2.2	0.10
		AST	16.7	0.02	0.001	17.8	0.5	0.02	18.1	1.5	0.07

*K* denotes the total number of studies/phenotypes. CPB denotes CPBayes and AST denotes ASSET. The number of true positives (out of 20 risk SNPs), number of false positives, and realized FDR are abbreviated as nTP, nFP and rFDR, respectively.

**Fig 1:**
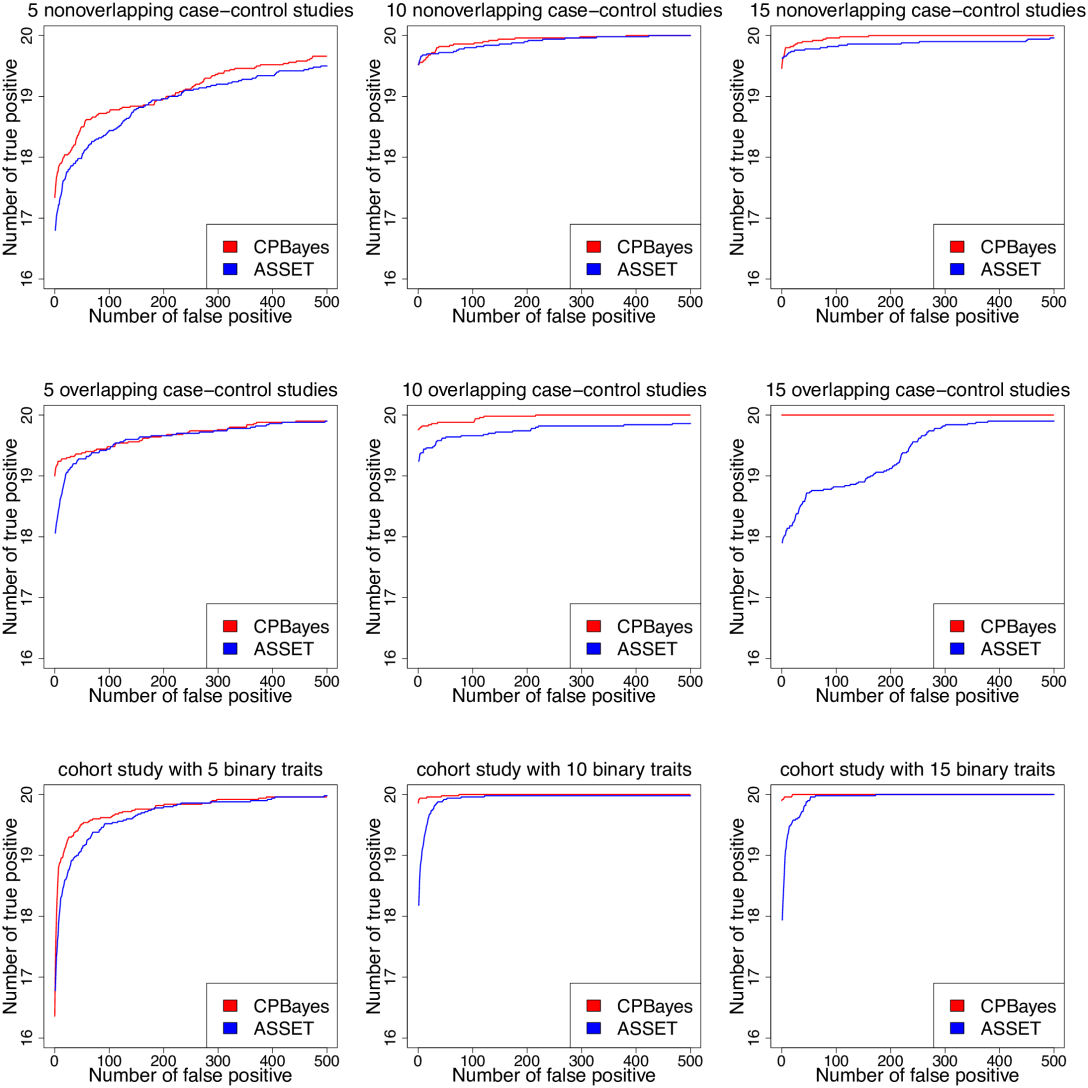
Simulation study results: Partial receiver operating characteristic (ROC) curves for CPBayes and ASSET. Number of true positives detected by CPBayes and ASSET at the expense of committing a given number of false positives are plotted. The number of false positives is varied across a range: 0, 1, …, 500. In each simulation scenario, 50 replications are performed to compute the mean number of true and false positives detected by each method.

In Fig 1, we present the partial ROC curves for CPBayes and ASSET. It shows that CPBayes has a greater AUC than ASSET consistently across different study designs and number of phenotypes. In Table 2, we provide the average number of true positives (nTP) and false positives (nFP) and the realized FDR (rFDR) detected by CPBayes based on different significance thresholds of locFDR in different simulation scenarios. As expected, rFDR is bounded above by the threshold of locFDR [27, 33]. We note that the estimated nFP and rFDR are zero in most of the cases. This is because we only considered 1000 SNPs for computational simplicity. From Table 1, we observe that both the methods control the FDR well overall (ASSET produced substantially inflated FDR only in one simulation scenario of five non-overlapping case-control studies). While the two methods detected similar number of true positives for non-overlapping case-control studies, CPBayes consistently identified a larger number of true positives than ASSET for overlapping case-control studies and cohort study (Table 1). We note that since the locFDR is directly estimated by the MCMC underlying CPBayes and it is the posterior probability of null association, we use it as the primary measure of evaluating aggregate-level pleiotropic association.

**Table 2:** Simulation results: The number of true positives and false positives detected by CPBayes at different significance levels of locFDR.

Study design		Significance level of locFDR								
		0.01			0.05			0.1		
	*K*	nTP	nFP	rFDR	nTP	nFP	rFDR	nTP	nFP	rFDR
Non-overlapping case-control studies	5	15.2	0	0	15.8	0	0	16.0	0	0
	10	18.0	0	0	18.4	0	0	18.6	0	0
	15	18.8	0	0	18.9	0	0	18.9	0	0
Overlapping case-control studies	5	14.7	0	0	15.6	0	0	16.1	0	0
	10	18.9	0	0	19.4	0.32	0.02	19.7	1.28	0.06
	15	18.9	0	0	19.0	0	0	19.2	0	0
Cohort study	5	13.5	0	0	14.9	0.04	0.002	15.7	0.2	0.01
	10	18.6	0	0	19.0	0	0	19.1	0	0
	15	19.4	0	0	19.5	0	0	19.6	0	0

*K* denotes the total number of studies/phenotypes. The number of true positives (out of 20 risk SNPs), number of false positives, and realized FDR are abbreviated as nTP, nFP and rFDR, respectively.

#### Selection accuracy for different methods

Here we assess the accuracy of selection of non-null traits underlying a pleiotropic signal detected by CPBayes and ASSET. We also applied BH_0.01_ (Material and methods) to select the optimal subset of non-null traits for a pleiotropic signal. We contrast the selection accuracy of CPBayes, ASSET and BH_0.01_ across a range of simulation scenarios. We consider multiple non-overlapping, overlapping case-control studies and cohort study with the same choices of *K* and *K*_1_ as provided above. For multiple case-control studies, we made an additional choice of *K*_1_ = 4 when *K* = 5, and *K*_1_ = 8 when *K* = 10.

Here we consider a single SNP with fixed MAF as 0.3 and 0.1, respectively. For a given choice of study design, *K* and *K*_1_, MAF at the SNP, we consider 500 replications in each of which OR for an associated trait is randomly simulated in the same way as described above. We separately consider the scenarios of all positive non-null effects and both positive and negative non-null effects to explore if the methods perform differentially under these two different scenarios. Suppose, $K_{1}^{+}$ traits are positively associated and $K_{1}^{-}$ traits are negatively associated ($K_{1}=K_{1}^{+}+K_{1}^{-}$). To ideally match with a GWAS setting, we should compute the average specificity and sensitivity of selected subset of traits only for the replications in which locFDR (or, p-value of ASSET) falls below a certain threshold showing an evidence of aggregate-level pleiotropic association. Hence, among the total of 500 replications, we consider only those replications in which the CPBayes locFDR is < 0.01. Based on these selected replications, we computed the average specificity and sensitivity of different methods while selecting the non-null traits. The minimum number of such selected replications was 153 for overlapping case-control studies with *K* = 5, $K_{1}^{+}=1$, $K_{1}^{-}=0$, *m* = 0.1. We note that it is also possible to select the replications based on a threshold of ASSET p-value instead of CPBayes locFDR. We explored this and observed that the overall conclusion about the selection accuracy of the methods remains unchanged.

**Fig 2:**
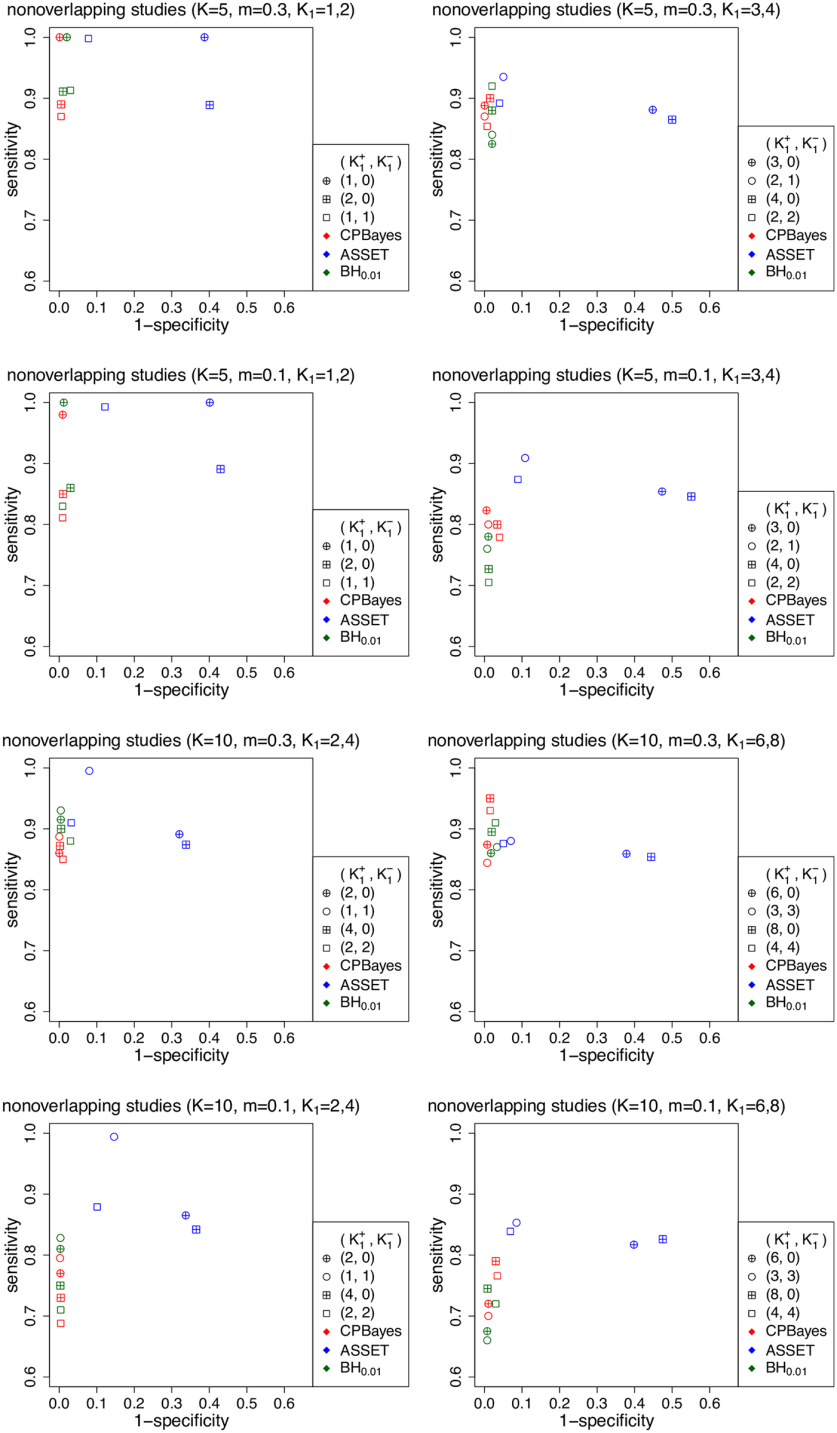
Simulation study results: Comparison of the accuracy of selection of associated traits by different methods for multiple non-overlapping case-control studies. The total number of studies is denoted by *K* and *m* denotes the minor allele frequency at the risk SNP. $K_{1}^{+}$ denotes the number of positively associated traits and $K_{1}^{-}$ denotes the number of negatively associated traits. Different type of points present different configurations of the number of associated traits and the number of positively and negatively associated traits.

**Fig 3:**
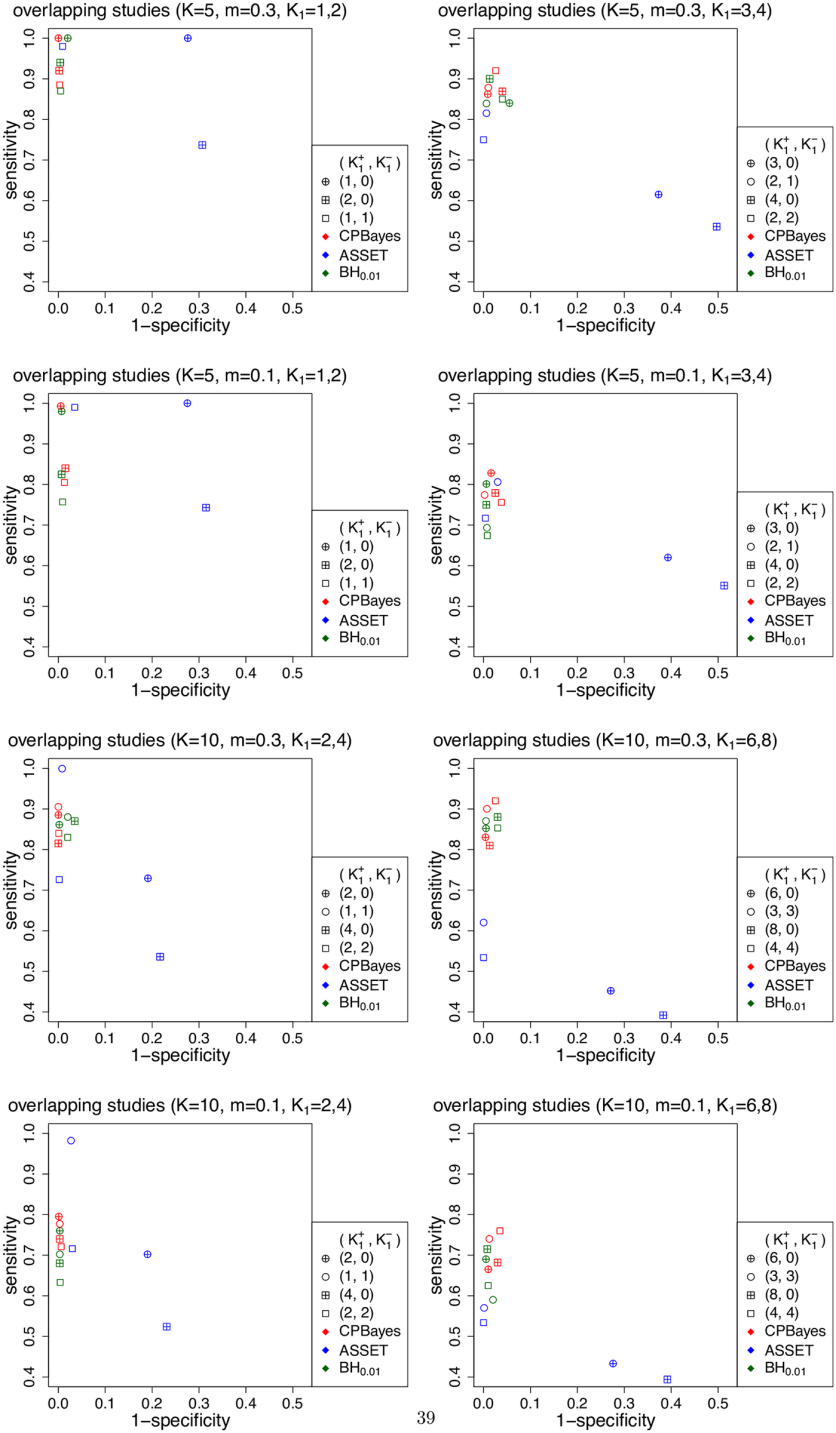
Simulation study results: Comparison of the accuracy of selection of associated traits by different methods for multiple overlapping case-control studies. The total number of studies is denoted by *K* and *m* denotes the minor allele frequency at the risk SNP. $K_{1}^{+}$ denotes the number of positively associated traits and $K_{1}^{-}$ denotes the number of negatively associated traits. Different type of points present different configurations of the number of associated traits and the number of positively and negatively associated traits.

First, we note that CPBayes and BH_0.01_ offer similar selection accuracy. In Figs 2 and 3, and S3, S4 and S5 Figs, points plotting the average specificity and sensitivity for CPBayes and BH_0.01_ across various simulation scenarios cluster around each other. So next, we focus on comparing the selection accuracies between the two main competing methods CPBayes and ASSET.

CPBayes yielded a very good level of specificity (consistently more than 94%) which is substantially higher than that of ASSET. For example, in Fig 2 (for non-overlapping case-control studies), while CPBayes’ specificity is > 96%, the specificity of ASSET varies in the range of 45%–98% when *K* = 5, and in the range of 53%–99% when *K* = 10. Similarly, in Fig 3 (for overlapping case-control studies), the specificity of ASSET varies in the range of 49%–100% when *K* = 5, and in 61%–100% when *K* = 10.

CPBayes also offers an overall good level of sensitivity. For 5 non-overlapping case-control studies (Fig 2), CPBayes produced a sensitivity of 87%–100% when MAF = 0.3 and 77%–100% when MAF = 0.1; ASSET yielded a similar sensitivity of 87%–100% (with 50%–98% specificity) when MAF = 0.3, and a slightly higher sensitivity of 85%–100% (with 45%–91% specificity) when MAF = 0.1. We observe a similar pattern for *K* = 10 (Fig 2) and *K* = 15 (S4 Fig). ASSET’s higher sensitivity than CPBayes appears to come at the expense of a substantially lower specificity.

For overlapping case-control studies (Fig 3 and S5 Fig), CPBayes gave a substantially better sensitivity than ASSET (along with substantially better specificity) for a majority of the simulation scenarios. For example, when *K* = 10 and *m* = 0.3, the sensitivity was 82%–89% for CPBayes, and 39%–73% for ASSET, except for the case $K_{1}^{+}=1,K_{1}^{-}=1$ when ASSET had 100% sensitivity (Fig 3). Similarly, for *K* = 10 and *m* = 0.1, the sensitivity was 68%–77% for CPBayes and 39%–72% for ASSET except for the choice $K_{1}^{+}=1,K_{1}^{-}=1$ when ASSET had 98% sensitivity (Fig 3). We observe a similar pattern for *K* = 15 (S5 Fig).

For a cohort study, CPBayes and ASSET both consistently exhibited good levels of sensitivity (S3 Fig). But, CPBayes produced a substantially higher sensitivity than ASSET in a number of cases. For all study designs, we also observed that when the non-null effects are both positive and negative, the specificity of ASSET is substantially higher compared to when the non-null effects are all positive. However, CPBayes performed more robustly with respect to the direction of non-null effects.

We also considered more stringent threshold of locFDR (e.g., 10^−4^) than 0.01 while selecting the replications showing evidence of pleiotropic association. We observed that for more stringent thresholds of locFDR, CPBayes provides a higher sensitivity compared to a larger threshold of locFDR while maintaining a good level of specificity. This is because a risk SNP will have a smaller locFDR if the odds ratios for the individual associated traits become stronger and this also leads to better sensitivity of the selected subset of associated traits for CPBayes while preserving a good level of specificity.

#### Comparison between CPBayes and GPA

We contrast CPBayes with GPA for two non-overlapping case-control studies. We have provided a summary of this comparison and a brief outline of the probabilistic model underlying GPA in supporting information (S1 Text and S6–S9 Figs). In summary, these two methods broadly agree with each other in terms of producing similar estimates of joint posterior probability of four possible configurations of association with two traits. However, GPA can wrongly conclude sometimes that both traits are associated even when only one of them is associated (S9 Fig and S1 Text), and hence can lead to lower specificity in selection. CPBayes and GPA use a mixture of two probability distributions—one to model null effects and the other to model non-null effects. While CPBayes directly models the effect estimates by a scale mixture of two normal distributions with mean zero (one with small variance to model the null effects), GPA models the univariate association p-values by a mixture of Uniform(0,1) distribution (modeling null effects) and a Beta distribution with its first shape parameter smaller than the second shape parameter (modeling non-null effects). This connection in the probabilistic modeling is a reason behind the similar performance of the two approaches. However, GPA is mainly suited for analyzing a pair of traits at a time, but CPBayes and ASSET are designed to analyze two or more traits simultaneously. For model fitting, GPA requires summary-level data for a sufficiently large number of GW SNPs which should include a substantial number of risk SNPs for better fitting. But CPBayes and ASSET can be implemented for any collection of SNPs individually.

### Application of CPBayes

To investigate the performance of CPBayes using real data, we analyzed multiple traits in the large Northern California Kaiser Permanente “Resource for Genetic Epidemiology Research on Adult Health and Aging” (GERA) cohort obtained from dbGaP [dbGaP Study Accession: phs000674.v1.p1]. We also analyzed this dataset using ASSET for an empirical comparison of the methods. We restricted our analysis to 62,318 European-American individuals, who constitute more than 75% of the dbGaP data. We tested 657,184 SNPs genotyped across 22 autosomal chromosomes for their potential pleiotropic effects on 22 case-control phenotypes in the GERA cohort (S8 Table). Note that in the dbGaP data, the cancers are collapsed into a single variable (any cancer). Therefore, we could only use an overall cancer categorization even though the genetic architecture is likely heterogeneous across different cancers. The phenotypes are correlated modestly with a maximum absolute value of correlation as 0.36 observed between Hypertension and Dyslipidemia.

Before our analysis, we undertook the following QC steps. First, we removed individuals with: over 3% of genotypes missing; any missing information on covariates (described below); genotype heterozygosity outside six standard deviations; first degree relatives; or discordant sex information. This left us with 53,809 individuals. Next, we removed SNPs with: MAF < 0.01; 10% or more missingness; or deviation from HWE at a level of significance 10^−5^. This leaves 601,175 SNPs that were tested for pleiotropic association by CPBayes and ASSET. We adjusted the analysis for the following covariates: age, gender, smoking status, BMI category and 10 principal components of ancestry (PCs). We tested the single-trait association for each of 22 phenotypes by a logistic regression of the case-control status on the genotype incorporating the same set of adjusting covariates. We used SNP-trait effect estimates (log odds ratios) and their standard errors in CPBayes and ASSET. As the summary statistics are correlated here, we used the combined strategy of CPBayes and the correlated version of ASSET.

Since we have environmental covariates in the GERA study, we estimated the correlation matrix of the effect estimates under the null using the GW summary statistics data [34]. First, we extracted all of the SNPs for which the trait-specific univariate p-value across 22 traits are > 0.1. This ensures that each SNP is either weakly or not associated with any of the 22 phenotypes. Then we selected a set of 24,510 independent SNPs from the initial set of null SNPs by using a linkage disequilibrium (LD) threshold of *r*^2^ < 0.01 (*r*: the correlation between the genotypes at a pair of SNPs). Finally, we computed the correlation matrix of the effect estimates as the sample correlation matrix of ${\hat{\beta }}_{1},\ldots ,{\hat{\beta }}_{22}$ across the selected 24,510 independent null SNPs. We also considered different SNP filtering thresholds and compared the resulting correlation matrices. We provide numerical results demonstrating that the estimated matrix was not sensitive to our primary choice of the thresholds. We also give numerical results indicating that the estimated correlation matrix based on the sample overlap counts (Eq 6) may be biased. These numerical results and their interpretation are provided in more detail in S9 Table and S1 Text.

We apply the conventional GW level of statistical significance 5 × 10^−8^ for ASSET. For CPBayes, a pre-fixed significance threshold of locFDR needs to be considered here. While concluding that a locFDR threshold of 5% indicates good evidence of association, selecting the most promising pleiotropic variants may require a more stringent threshold (as with the frequentist p-value threshold 5 × 10^−8^). Liley and Wallace [12] also highlighted this point and suggested using a more stringent threshold of conditional false discovery rate (cFDR) than the nominal levels (e.g., 0.05 or 0.01). They used a FDR and cFDR cut-off in the order of 10^−5^ or 10^−6^ to make the analysis analogous to using a stringent threshold of p-value (5 × 10^−8^). We applied various thresholds of locFDR and detected 610 (locFDR < 10^−2^), 537 (locFDR < 10^−3^), 523 (locFDR < 10^−4^), 481 (locFDR < 10^−5^), 442 (locFDR < 10^−6^), 417 (locFDR < 10^−7^) and 380 (locFDR < 10^−8^) SNPs, respectively. We note that locFDR is not used as extensively as p-value in practice. Unlike the commonly used p-value threshold 5 × 10^−8^, different thresholds of locFDR (or other FDR related measures) have been used in different applications [8, 9, 12]. Since Liley and Wallace [12] used a FDR and cFDR cut-off in the order of 10^−5^ or 10^−6^, here we report the results based on a similar threshold of locFDR as 10^−6^ which detected 442 SNPs. Of note, locFDR, FDR and cFDR are distinct by definition. Theoretically, locFDR is an upper bound of FDR [27]. ASSET detected 394 SNPs based on the p-value threshold 5 × 10^−8^. We note that CPBayes and ASSET identified a common set of 322 SNPs based on these chosen significance thresholds.

Many of the associated SNPs are expected to be in LD. It is challenging to report independent pleiotropic variants since the LD pattern across a chromosome is irregular and converting the conditional analysis approach used in single phenotype GWAS to select independently associated variants in multi-phenotype context is difficult. Hence for the sake of convenience, we undertook the following simplified approach to identify correlated LD blocks. For CPBayes (ASSET) on each chromosome, we first chose the associated SNP that has the minimum locFDR (ASSET p-value) and created a LD block around it using a threshold of *r*^2^ = 0.25. Then we implement the same strategy on the remaining set of associated SNPs to identify the next LD block, and so on. A major limitation of this approach is that defining such discrete LD blocks may not be on par with the irregular LD pattern across a chromosome and choosing an appropriate threshold of *r*^2^ is also difficult. For CPBayes, 442 GW associated SNPs comprised 59 LD blocks, and for ASSET, 394 GW associated SNPs comprised 30 LD blocks.

For each of 394 SNPs detected by ASSET, the optimal subset of non-null traits always included more than one trait. Within each LD block detected by ASSET, we chose the SNP associated with the maximum number of traits as the lead SNP of the block. If multiple SNPs in a LD block satisfy this criterion, the one with minimum p-value of pleiotropic association was selected. We present the results for the lead SNPs detected by ASSET only on chromosome 1 and 2 (to save space) in S11 Table.

CPBayes selected more than one trait for 93 among 442 SNPs. Within each LD block identified by CPBayes, we chose the SNP associated with the maximum number of phenotypes as the lead SNP of the block. If multiple SNPs satisfy this criterion, we chose the one having the minimum locFDR. In addition, if every SNP in a LD block is associated with one trait, we chose the SNP which provided the maximum number of traits having the marginal trait-specific posterior probability of association (PPA_*j*_) > 20% (these traits were termed important phenotypes). Again, if multiple SNPs satisfy this criterion, we chose the one having the minimum locFDR.

We note that the strategy of choosing the lead SNP in a LD block identified by CPBayes and ASSET are similar but technically different. As CPBayes locFDR and ASSET p-value are not directly comparable, it is very difficult to contrast the two methods with respect to the power of detecting aggregate-level pleiotropic association in a real data application. However, it makes sense to contrast the selection of non-null traits at a pleiotropic variant detected by both the methods.

In Table 3, we present the results for the independent pleiotropic SNPs at which CPBayes selected at least two phenotypes. At some of the lead SNPs detected by CPBayes, some phenotypes produced a non-negligible value of PPA_*j*_ but were left out of the optimal subset of non-null traits. In Table 4, we list these SNPs and the corresponding important phenotypes having a PPA_*j*_ > 20%. In S10 Table, we report the independent SNPs at which CPBayes selected one trait. In the tables for CPBayes, we present PPA_*j*_ and the direction of association (genotype was coded as the number of the wild allele) for the selected phenotypes (Material and methods). In all the tables for CPBayes and ASSET, we also provide the trait-specific univariate association p-values.

**Table 3:** Independent pleiotropic SNPs detected by CPBayes which are associated with at least two phenotypes.

rsID	chrom band	CPBayes locFDR	CPBayes log_10_BF	Subset of associated traits selected by CPBayes	PPA_*j*_	Direction	Univariate p-values
rs6025	1q24.2	5.06 × 10^−224^	220.83	Dermatophytosis	67%	positive	0.0018
				Hemorrhoids	71%	positive	0.0014
				Iron Deficiency	97%	positive	0.0004
				Osteoporosis	94%	negative	0.0002
				Peripheral Vascular Disease	100%	negative	6.81 × 10^−14^
rs7601401	2p16.1	6.27 × 10^−19^	17.24	Abdominal Hernia	100%	positive	3.88 × 10^−12^
				Osteoarthritis	68%	positive	3.46 × 10^−6^
rs13211628	6p21.32	1.99 × 10^−10^	8.32	Asthma	98%	positive	1.71 × 10^−7^
				Cancers	59%	negative	6.71 × 10^−5^
				Dyslipidemia	100%	negative	2.09 × 10^−10^
rs10455872	6q25.3	1.04 × 10^−25^	23.60	Cardiovascular Disease	62%	negative	6.14 × 10^−5^
				Dyslipidemia	100%	negative	6.97 × 10^−15^
				Peripheral Vascular Disease	61%	negative	0.0002
rs3957148	6p21.32	1.68 × 10^−21^	19.39	Asthma	100%	negative	7.10 × 10^−8^
				Type 2 Diabetes	100%	negative	4.74 × 10^−6^
				Macular Degeneration	62%	positive	0.0006
rs687289	9q34.2	1.11 × 10^−8^	6.57	Type 2 Diabetes	65%	negative	0.0002
				Dyslipidemia	100%	negative	1.21 × 10^−11^
				Peripheral Vascular Disease	95%	negative	7.25 × 10^−6^

The chromosome band of a SNP is denoted by ‘chrom band’. Direction means whether the SNP is positively or negatively associated with the phenotype. PPA_*j*_ denotes the marginal trait-specific posterior probability of association with a risk SNP.

**Table 4:** Pleiotropy results by CPBayes for those SNPs at which some phenotypes were not selected in the optimal subset of non-null traits but produced a non-negligible value of trait-specific posterior probability of association (PPA_*j*_).

rsID	chrom band	CPBayes locFDR	CPBayes log_10_BF	Important phenotypes	PPA_*j*_	Univariate p-values	Subset of associated traits detected by BH_0.01_
rs1410996	1q31.3	1.00 × 10^−300^	300.00	Macular Degeneration	100%	1.86 × 10^−75^	Macular Degeneration
				Iron Deficiency	20%	0.0008	Iron Deficiency
rs17647543	1p13.3	1.96 × 10^−10^	8.75	Dyslipidemia	100%	5.29 × 10^−9^	Dyslipidemia
				Peptic Ulcer	36%	0.004	
rs115946033	3q25.32	1.97 × 10^−10^	8.74	Type 2 Diabetes	100%	3.83 × 10^−7^	Type 2 Diabetes
				Depressive Disorder	23%	0.0008	Depressive Disorder
rs387608	6p21.33	1.94 × 10^−22^	20.75	Macular Degeneration	100%	4.29 × 10^−12^	Macular Degeneration
				Cancers	42%	0.0001	Cancers
rs849142	7p15.1	5.56 × 10^−13^	11.29	Type 2 Diabetes	100%	1.94 × 10^−14^	Type 2 Diabetes
				Asthma	28%	2.89 × 10^−5^	Asthma
rs10808546	8q24.13	5.49 × 10^−16^	14.30	Dyslipidemia	100%	6.00 × 10^−25^	Dyslipidemia
				Hypertension	21%	2.59 × 10^−5^	Hypertension
rs687289	9q34.2	1.1 × 10^−8^	6.57	Type 2 Diabetes	65%	0.0002	Type 2 Diabetes
				Dyslipidemia	100%	1.2 × 10^−11^	Dyslipidemia
				Peripheral Vascular Disease	95%	7.3 × 10^−6^	Peripheral Vascular Disease
				Peptic Ulcer	28.4%	0.003	
rs4506565	10q25.2	1.60 × 10^−117^	115.84	Type 2 Diabetes	100%	2.02 × 10^−55^	Type 2 Diabetes
				Dyslipidemia	52%	1.33 × 10^−6^	Dyslipidemia
rs76075198	19q13.31	5.58 × 10^−36^	34.29	Dyslipidemia	100%	5.29 × 10^−11^	Dyslipidemia
				Peripheral Vascular Disease	34%	0.007	

The chromosome band of a SNP is denoted by ‘chrom band’. PPA_*j*_ denotes the marginal trait-specific posterior probability of association with a risk SNP.

Many of the pleiotropic variants detected by CPBayes and ASSET are already reported in the NHGRI-EBI GWAS catalog. For example, rs6025 at 1q24.2 (Table 3) has been associated with inflammatory bowel disease and venous thromboembolism; rs10455872 at 6q25.3 (Table 3) has been associated with myocardial infarction, response to statins (LDL cholesterol change), coronary artery disease, and aortic valve calcification; rs1410996 at 1q31.3 (Table 4) is reported to be associated with Post bronchodilator FEV1/FVC ratio in COPD, End-stage coagulation and Age-related Macular Degeneration; rs4506565 at 10q25.2 (Table 4) has been associated with Fasting glucose-related traits and Type 2 Diabetes.

For a majority of the SNPs detected by ASSET, the subset of non-null traits included many phenotypes that have large univariate association p-values. For example, rs77394225 at 1q31.3 was detected by both the methods (S10 and S11 Tables); ASSET selected 10 traits, 9 of which had univariate p-value ≥ 0.3 and one (Macular Degeneration) had p-value = 6.04 × 10^−16^ (S11 Table). In contrast, CPBayes only selected Macular Degeneration (S10 Table). This suggests that CPBayes selects only those phenotypes with strong genetic associations, while ASSET may select many more traits with lower specificity as seen in our simulation study. For the independent pleiotropic SNPs identified by both the methods, for contrast’s sake, we applied BH_0.01_. At rs77394225, BH_0.01_ only selected Macular Degeneration which is consistent with CPBayes. Among the nine independent pleiotropic SNPs on chromosome 1 and 2 detected by ASSET, BH_0.01_ selected more than one trait only for two SNPs, whereas ASSET selected multiple traits for each of them (S11 Table). This again indicates lower specificity of ASSET.

**Fig 4:**
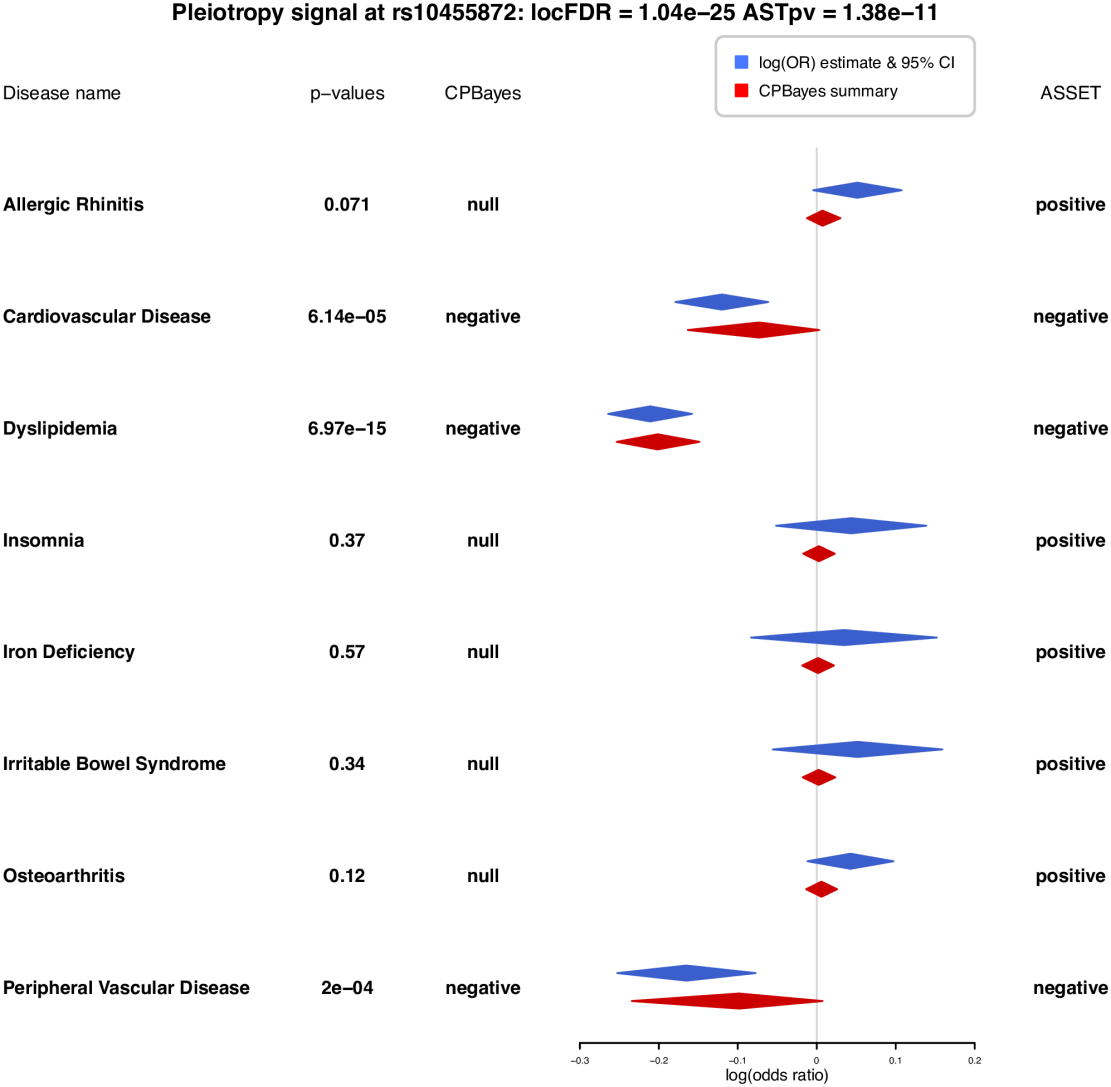
Forest plot for pleiotropic signal at rs10455872 on chromosome 6 contrasting the selection of traits by CPBayes and ASSET. Phenotypes selected by either of the two methods are plotted. Blue diamonds present the trait-specific univariate log odds ratio estimate with the corresponding 95% confidence interval. Red diamonds present the posterior mean and 95% credible interval of the trait-specific log odds ratio obtained by CPBayes. The CPBayes locFDR and ASSET p-value (ASTpv) are provided. The association status of a phenotype detected by a method is denoted by null (not associated), positive or negative (associated). The trait-specific univariate association p-values are also provided.

CPBayes detected six independent pleiotropic SNPs that were associated with at least two phenotypes (Table 3). For example, at rs6025 (1q24.2), it selected a maximum of 5 phenotypes: Dermatophytosis, Hemorrhoids, Iron Deficiency, Osteoporosis and Peripheral Vascular Disease, which have univariate p-values equal to 0.0018, 0.0014, 0.0004, 0.0002 and 6.81 × 10^−14^, respectively (Table 3). Interestingly, this SNP was positively associated with Dermatophytosis, Hemorrhoids and Iron Deficiency, but negatively associated with Osteoporosis and Peripheral Vascular Disease (Table 3). rs10455872 at 6q25.3 appeared to be the lead SNP for both the methods in their corresponding LD block on chromosome 6. In Fig 4, we contrast the selection of traits at rs10455872 between the methods. CPBayes selected Cardiovascular Disease, Dyslipidemia and Peripheral Vascular Disease (Fig 4). ASSET selected these three traits and five more phenotypes with large univariate p-values indicating weak genetic effects (Fig 4).

**Fig 5:**
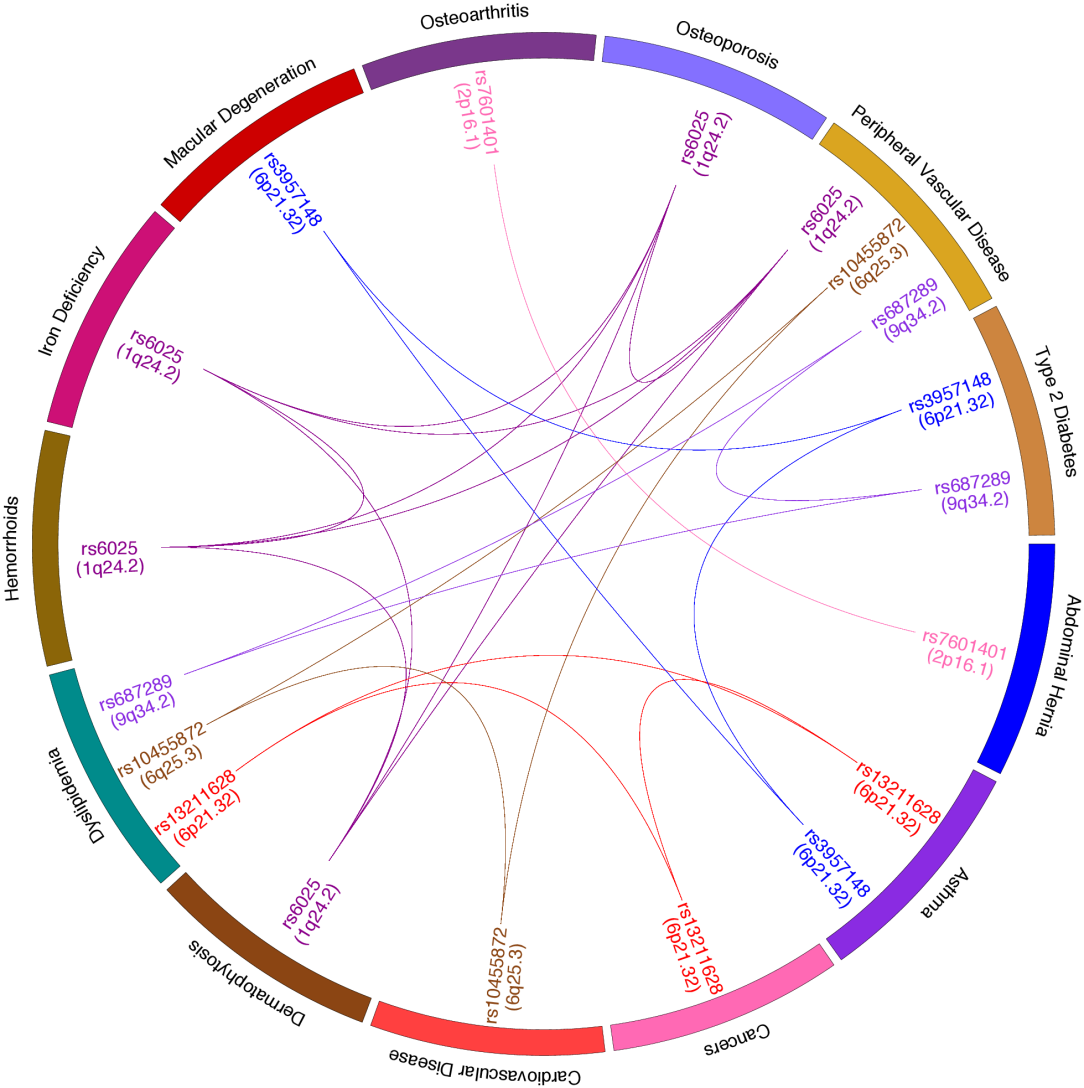
A circos plot presenting the pairwise trait-trait pleiotropic signals detected by CPBayes in GERA cohort.

CPBayes detected two independent pleiotropic SNPs in the chromosomal region 6p21.32: rs13211628 which was associated with Asthma, Cancers and Dyslipidemia, and rs3957148 which was associated with Asthma, Type 2 Diabetes and Macular Degeneration (Table 3). The *r*^2^ value between rs13211628 and rs3957148 was 0.03. We provide a circos plot in Fig 5 presenting 23 pair-wise trait-trait pleiotropic association signals detected by CPBayes. It shows that CPBayes detected rs7601401 at 2p16.1 associated with Osteoarthritis and Abdominal Hernia; rs687289 at 9q34.2 associated with Dyslipidemia, Type 2 Diabetes and Peripheral Vascular Disease. We also present forest plot for some of the independent pleiotropic signals (at rs6025, rs13211628, rs10455872, rs3957148, rs687289) detected by CPBayes in S11–S15 Figs. For these six independent pleiotropic SNPs, BH_0.01_ selected the same subset of traits as CPBayes.

For CPBayes, the marginal trait-specific posterior probability of association (PPA_*j*_) provides a better insight into the relative strength of association between a pleiotropic variant and the selected non-null traits. For example at rs6025, PPA_*j*_ for Dermatophytosis, Hemorrhoids, Iron Deficiency, Osteoporosis and Peripheral Vascular Disease are 67%, 71%, 97%, 94% and 100%, respectively (Table 3). This implies that the association with Peripheral Vascular Disease is the strongest among the five selected phenotypes.

At some of the GW significant SNPs detected by CPBayes, it produced a non-negligible value of PPA_*j*_ for some traits; but these traits were left out of the optimal subset of non-null traits (Table 4). For example at rs4506565 (10q25.2), CPBayes only selected Type 2 Diabetes, but Dyslipidemia also produced a PPA_*j*_ of 52%. Thus, even though the effect of rs4506565 on Dyslipidemia was not strong enough to make it into the optimal subset, a further consideration of the pleiotropic effect of rs4506565 on Type 2 Diabetes and Dyslipidemia looks promising. We observed similar pattern across the other pleiotropic variants listed in Table 4. We note that the combined strategy of CPBayes used the uncorrelated version only for one SNP among all the 601,175 SNPs analyzed.

#### Computational efficiency

For a larger number of phenotypes, CPBayes is computationally faster than ASSET. For example, in the analysis of 22 traits in the GERA cohort, CPBayes took an average run time of 3.5 hours for 1,000 SNPs, and ASSET took an average run time of 9 hours for 1,000 SNPs. However, as the number of traits decreases, ASSET gradually becomes faster due to the reduction in the number of all possible subsets of traits. That said, CPBayes is computationally feasible and can be implemented at a GW scale. As expected, the uncorrelated version of CPBayes is at least twice as fast as the correlated version of CPBayes.

## Discussion

We have proposed a Bayesian meta-analysis approach CPBayes for pleiotropic association analysis based on summary-level data. It simultaneously evaluates the evidence of aggregate-level pleiotropic association and estimates an optimal subset of traits associated with the risk locus under a unified Bayesian statistical framework. The method is implemented by Gibbs sampling designed for both uncorrelated and correlated summary statistics. We have conducted an extensive simulation study and analyzed the large GERA cohort for evaluating the performance of CPBayes.

An appealing feature of CPBayes is that, in addition to locFDR, Bayes factor, and an optimal subset of non-null traits, it simultaneously provides other interesting insights into an observed pleiotropic signal. For example, it estimates a trait-specific posterior probability of association (PPA_*j*_), the direction of association, posterior mean/median and the credible interval of the unknown true genetic effect across traits. PPA_*j*_ quantifies the marginal probability of each trait being associated with a pleiotropic variant. As demonstrated in the real data application, even if CPBayes does not select a phenotype in the optimal subset of non-null traits, PPA_*j*_ for the phenotype may not be negligible. This may help an investigator to better explain a pleiotropic signal. One can also define the optimal subset of associated traits as {*Y*_*j*_: PPA_*j*_ > *p*}, where *p* can be chosen as 0.5 (known as the median model), or other values. Moreover, the joint posterior probability of association for a particular subset of traits can be calculated. Such flexibility in making inference on pleiotropy is mainly due to the MCMC construction underlying CPBayes.

CPBayes selects the non-null traits underlying a pleiotropic signal with higher accuracy than ASSET. CPBayes performs the selection probabilistically through updating the latent association status by MCMC. ASSET selects that subset of traits as non-null which maximizes the observed value of a weighted linear combination of the normalized univariate association statistics corresponding to the phenotypes belonging to a subset. So given the summary statistics, ASSET does not select the non-null traits probabilistically based on the distribution of the summary statistics. For ASSET, even if a trait having a small genetic effect contributes a little to a pleiotropic signal, it is included in the optimal subset of associated traits. But CPBayes considers only those traits as non-null which substantially contribute to a pleiotropic signal. For example in the real data application, at rs10455872, allergic rhinitis had an estimated odds ratio 1.05 (univariate association p-value 0.07). ASSET included this phenotype in the optimal subset of non-null traits as it might contribute to the overall signal of pleiotropic assocition. However, CPBayes estimated PPA_*j*_ for this trait as 2.4% (S13 Fig). So its contribution to the pleiotropic signal was not necessarily null, but CPBayes did not include it in the optimal subset of non-null traits as the effect was weak. We also note that ASSET is based on the framework of a fixed effects meta-analysis and assumes that the effects in a given direction (positive/negative) have the same size. But we observed in our real data application that, in a given direction, the effects of a variant across phenotypes may often be heterogeneous. CPBayes allows for heterogeneity simultaneously in the direction and size of the effects. S2 Table summarizes the key features of CPBayes and ASSET.

While assessing the selection accuracy, we have placed more emphasis on specificity than sensitivity. This was because a higher sensitivity at the expense of a lower specificity can lead to a false selection of too many traits as non-null. CPBayes consistently maintained a very good level of specificity while offering a good level of sensitivity across a wide range of simulation scenarios. While CPBayes produced a limited number of independent pleiotropic SNPs associated with more than one phenotype in the analysis of GERA cohort, these pleiotropic signals seem very promising. The subsets of non-null traits selected by BH_0.01_ in the GERA cohort were consistent with CPBayes but not with ASSET which indicates that the non-null traits for a pleiotropic variant selected by CPBayes may be more reliable than ASSET. Here we note that, BH_0.01_ only facilitates the selection of associated traits underlying a pleiotropic signal but can not test for the evidence of overall pleiotropic association as CPBayes and ASSET. While CPBayes and ASSET estimate the measure of aggregate-level pleiotropic association and subset of non-null traits simultaneously under the same framework, BH_0.01_ has to be implemented in a separate step for a GW significant pleiotropic variant. Hence, CPBayes is a substantially more complete statistical tool for pleiotropy analysis than BH_0.01_.

While evaluating the selection accuracy of different approaches by simulations, we simultaneously obtained the measures of overall pleiotropic association provided by CPBayes and ASSET across 500 replications. We present various summary measures of these in some selected simulation scenarios (to save space) and corresponding brief interpretation in S3, S4 and S5 Tables and S1 Text. We also carried out simulations for 50 traits. Since ASSET is computationally very slow for 50 traits due to an extremely large number of possible subsets of traits, we only implemented CPBayes and BH_0.01_. CPBayes performed consistently well similarly as for smaller number of traits. See S1 Text and S6 and S7 Tables for more details.

Note that the continuous spike inherits the assumption that a SNP contributes to the variation of all traits under consideration, and the distinction is made between a negligible and a significant contribution. In contrast, the Dirac spike assigns the null effects explicitly to zero. We conducted a simulation study (see S1 Text and S10 Fig) to compare the continuous spike and Dirac spike. We found that the continuous spike offers better accuracy in the selection of non-null traits than the Dirac spike. The continuous spike is also computationally much faster (2-3 times) than the Dirac spike. Hence, we adopted the continuous spike for constructing CPBayes.

In a related work, Han and Eskin [35] proposed a modified random effects meta-analysis for combining heterogeneous studies coupled with a Bayesian approach to provide a better interpretation of an observed signal of aggregate-level association. They investigated how to combine heterogeneous genetic studies across different populations/ethnicities. However, they did not address how to account for a possible correlation between the summary statistics while selecting the most important studies underlying an observed signal of aggregate-level association. Moreover, they assumed that the non-null effects are similar across studies which is less likely to hold in the context of pleiotropy. Hence we compared CPBayes with ASSET and GPA.

We note that the CPBayes locFDR (Bayes factor) and ASSET p-value are not directly comparable. In our simulation study, we adopted the strategy outlined by Coram et al. [33] to compare CPBayes and ASSET with respect to the number of true positives detected while maintaining the FDR at a pre-specified threshold. Another possible approach to quantify the false positive rate could be the permutation-based strategy suggested by Servin and Stephens [36]. However in our context, such an approach is computationally too expensive as it requires the MCMC underlying CPBayes to be implemented for each permuted dataset. Of note, we did not conduct a replication study and all the pleiotropic association signals obtained from the Kaiser cohort are reported based on the analysis only in the discovery sample.

Even though we demonstrated CPBayes only for binary traits, we note that CPBayes can also be applied to non-binary traits. We carried out simulations for continuous traits (distributed as multivariate normal), and observed a similar pattern between the performance of CPBayes and ASSET as for binary traits.

Another useful approach to pleiotropy analysis is MultiPhen [37] which can accommodate general types of traits. However, we chose ASSET as the main competing method because it simultaneously provides an optimal subset of associated traits along with a measure of aggregate-level pleiotropic association, which provides a direct comparison to CPBayes. In contrast, MultiPhen does not facilitate the simultaneous selection of the optimal associated traits underlying a pleiotropic signal. Of note, MultiPhen requires individual level phenotype and genotype data.

In the analysis of GERA cohort, we assumed that the correlation matrix of estimated effect sizes is the same across SNPs, for each of which, we converted this correlation matrix to its covariance matrix by incorporating its standard error across traits. Hence, if the standard error vector varies across SNPs, the covariance matrix of the effect estimates also varies. We did some simulations to assess this assumption of constant correlation matrix. Consider the simulation framework of a cohort study with five case-control phenotypes designed to evaluate the partial ROC curves for CPBayes. Here the continuous traits (liability) underlying the binary traits were generated at random for every SNP following the simulation model in S1 Text; hence the binary traits dataset varied across SNPs. So the correlation matrix of effect estimates obtained by Eq 6 also varied across SNPs. We implemented CPBayes for each SNP using the corresponding effect estimates’ correlation matrix obtained by Eq 6 which is accurate for the null SNPs as there are no environmental covariates here. In the simulated data for a single risk SNP, we anticipate that the real correlation matrix of the effect estimates will be very close to the sample overlap correlation matrix (Eq 6). Because, a single risk SNP usually explains a very small proportion of total heritability for a complex trait and the genetic correlation between two traits due to a single risk SNP is expected to be very small. Next we estimated a constant correlation matrix as the sample correlation matrix of the observed effect estimates for the null SNPs (following the GW strategy in GERA cohort analysis), and implemented CPBayes for all the SNPs using this constant correlation matrix. We observed that the partial ROC curve for CPBayes obtained by using varying correlation matrices and constant correlation matrix across SNPs had almost the same AUC. We also found by simulations that using the constant correlation matrix provides a very similar selection accuracy compared to using varying correlation matrix across SNPs. We repeated these simulations for a cohort study with five continuous phenotypes distributed as multivariate normal. Since the phenotypes are normally distributed, the real correlation matrix of effect estimates can be analytically computed. Again, CPBayes performed very similarly using constant and varying correlation matrices.

If a set of binary traits are measured on separate independent group of individuals, one would expect the summary statistics across traits be independent. However, if these traits are mutually exclusive because of competing risks, risk SNPs for one trait may be underrepresented among the cases of the other trait, leading to a correlation in the summary statistics, especially for high penetrance variants. For common and complex traits, competing risks may not result in mutual exclusivity, and may lead to very limited correlation among subjects from independent samples. Moreover, since such traits arise from many different low risk SNPs, any corresponding correlation among summary statistics would also be limited. Taken together, we expect that this would have negligible impact on our assessment of pleiotropy, although separate studies may consider this possibility further.

Since CPBayes individually analyzes each SNP using marginal summary statistics across traits, it can not distinguish between pleiotropy and co-localization. Any marginal SNP-level meta analysis approach including ASSET also has this limitation. We analyzed 22 traits in the GERA cohort. Ideally one would only include genetically correlated traits in a pleiotropy analysis to maximize the power of a multi-trait approach. However, determining a priori which traits are genetically related can be challenging. This could be based on the literature or estimated from one’s own data. In the latter situation, one must be cognizant of potential bias due to empirically determining genetic correlations. To date, most pleiotropy analyses have focused on a small set of context-specific traits (e.g., psychiatric disorders, cancers). Expanding to larger numbers of disparate traits may provide important insights to shared biological mechanisms. In general, the selection of traits to consider for pleiotropy analyses can be based on co-heritability (genetic correlation) analyses, existing literature, and biological / clinical expertise.

In future work, we aim to investigate whether the computing speed of CPBayes can be increased by using a variational Bayes approach or by using an optimization technique (e.g., EM algorithm or its variants) instead of using MCMC, while preserving the efficiency of the method. Also, we want to explore how to relax the assumption in CPBayes that $s_{j}^{2}$ is a reasonably accurate estimate of $\sigma_{j}^{2}$ which requires a larger sample size to be satisfied. In summary, CPBayes is an efficient Bayesian meta-analysis approach to simultaneously analyze pleiotropy for two or more traits. It has a strong theoretical foundation and allows for heterogeneity in both the direction and size of effects. One can implement it for both cohort data and separate studies of multiple phenotypes having non-overlapping or overlapping subjects. In addition to parameters of primary interest (e.g., the measures of overall pleiotropic association, the optimal subset of associated traits), it provides other interesting insights into a pleiotropic signal (e.g., the trait-specific posterior probability of association, the direction of association, the credible interval of unknown true genetic effect across traits). It is computationally feasible and a user-friendly R-package ‘CPBayes’ is provided for general use.

## Materials and methods

Let *Y*_1_, …, *Y*_*K*_ denote *K* phenotypes, *G* denote genotype at a single nucleotide polymorphism (SNP), and *W* denote a set of covariates. For the SNP, assume a generalized linear model (GLM) is separately fit for each phenotype as: *g*(*E*(*Y*_*j*_)) = *α*_*j*_ + *β*_*j*_ *G* + *γ*_j_′*W*, *j* = 1, …, *K*. Let ${\hat{\beta }}_{1},\ldots ,{\hat{\beta }}_{K}$ denote the estimates (e.g., maximum likelihood estimates) of *β*_1_, …, *β*_*K*_ with the corresponding standard errors *s*_1_, …, *s*_*K*_. Let $\hat{\beta }=\left({\hat{\beta }}_{1},\ldots ,{\hat{\beta }}_{K}\right)$, ***β*** = (*β*_1_, …, *β*_*K*_), and ***s*** = (*s*_1_, …, *s*_*K*_). Now suppose that we only have the summary statistics (e.g., $\hat{\beta }$ and ***s***). For a large sample size, we can assume that ${\hat{\beta }}_{j}|\beta_{j},\sigma_{j}∼\text{N}\left(\beta_{j},\sigma_{j}^{2}\right)$. Since *s*_*j*_ is a consistent estimator of *σ*_*j*_, it is commonly used in place of *σ*_*j*_. Hence, we assume that ${\hat{\beta }}_{j}|\beta_{j}∼\text{N}\left(\beta_{j},s_{j}^{2}\right)$. If ${\hat{\beta }}_{1},\ldots ,{\hat{\beta }}_{K}$ are uncorrelated, ${\hat{\beta }}_{j}|\beta_{j}\;\underset{˜}{ind}\;\text{N}(\beta_{j},s_{j}^{2});$ *j* = 1, …, *K*. If ${\hat{\beta }}_{1},\ldots ,{\hat{\beta }}_{K}$ are correlated with a covariance matrix *S* corresponding to the SNP, we assume that $\hat{\beta }|\beta ∼\text{MVN}\left(\beta ,S\right)$. Since the maximum likelihood estimator asymptotically follows a normal distribution and *S* is a consistent estimator of the variance-covariance matrix of $\hat{\beta }$, these assumptions should hold well for large sample sizes in contemporary GWAS.

### Continuous spike

The continuous spike and slab prior in our context [19, 20] is described as follows: for *j* = 1, …, *K*,

$$\begin{array}{l} \beta_{j}|z_{j},\tau ,d\;\underset{˜}{ind}\;(1-z_{j})\times N(0,\tau^{2})+z_{j}\times N(0,{\left(\frac{\tau }{d}\right)}^{2});\;\tau >0,\;0<d<1,\;{\left(\frac{\tau }{d}\right)}^{2}>\tau^{2}\\ P(z_{j}=1|q)=q;\;P(z_{j}=0|q)=(1-q);\;0<q<1\\ q|c_{1},c_{2}\sim \text{Beta}(c_{1},c_{2});\;d|e_{1},e_{2}\sim \text{Beta}(e_{1},e_{2}) \end{array}$$ (1)

The latent variable *z*_*j*_ denotes the association status of *Y*_*j*_. When *z*_*j*_ = 0, *β*_*j*_ ∼ *N*(0, *τ*^2^), and when *z*_*j*_ = 1, $\beta_{j}∼N\left(0,{\left(\frac{\tau }{d}\right)}^{2}\right)$, where ${\left(\frac{\tau }{d}\right)}^{2}>\tau^{2}$. The usefulness of such a formulation is that *τ* can be set small enough so that, if *z*_*j*_ = 0, |*β*_*j*_| would probably be very small to safely be considered as zero (*Y*_*j*_ is not associated with the SNP), and *d* can be chosen sufficiently small (so $\frac{1}{d}>>1$) such that, if *z*_*j*_ = 1, *β*_*j*_ can be considered as non-zero (*Y*_*j*_ is associated with the SNP). The proportion of traits having a non-null genetic effect is denoted by *q*. For simplicity and reduction in computational cost, we consider *τ* as fixed. We choose *e*_1_ = *e*_2_ = 1 which correspond to the uniform(0, 1) distribution. The parameter *d* is updated in a given range so that the slab variance ${\left(\frac{\tau }{d}\right)}^{2}$ (i.e., variance of non-null effects across traits) varies in a pre-fixed range. We describe the continuous spike and slab prior in the context of modeling pleiotropy with diagrams in S1 and S2 Figs.

### Dirac spike

The Dirac spike and slab prior in current context [18, 20] is given by: for *j* = 1, …, *K*,

$$\begin{array}{c} \beta_{j}|q,b\;\underset{˜}{i.i.d.}\;(1-q)\times \delta_{\left\{0\right\}}(\beta_{j})+q\times \text{N}(0,b^{2})\\ q|c_{1},c_{2}\sim \text{Beta}(c_{1},c_{2}),0<q<1 \end{array}$$ (2)

Here, *δ*_{0}_(*β*_*j*_) = 1 if *β*_*j*_ = 0, and *δ*_{0}_(*β*_*j*_) = 0 if *β*_*j*_ ≠ 0. So under no association, *β*_*j*_ = 0. The proportion of associated traits is given by *q*.

### Statistical inference on pleiotropy by employing MCMC

To perform a fully Bayesian analysis, we implement MCMC by the Gibbs sampling algorithm to generate posterior samples of the model parameters based on which we draw the statistical inference for pleiotropy. We derive the Gibbs samplers for both uncorrelated and correlated summary statistics. Here we describe the inference procedure for the continuous spike. The Gibbs sampling algorithm for the continuous spike (Algorithm 1) is outlined later in this section, and the algorithm for Dirac spike (Algorithm S1) is stated in supporting information (S1 Text). The mathematical derivation of the full conditional posterior distributions underlying the Gibbs samplers are also given in supporting information (S1 Text).

Let {***β***^(*i*)^, *Z*^(*i*)^, *q*^(*i*)^, *d*^(*i*)^; *i* = 1, …, *N*} denote *N* posterior samples of (***β***, *Z*, *q*, *d*) obtained by MCMC after a certain burn-in period. We have used a burn-in period of 5000 and MCMC sample size of 15000 in our simulation study and the real data application. First, we want to test the global null hypothesis of no association (*H*_0_) against the global alternative hypothesis of association with at least one trait (*H*_1_). Since, for the continuous spike, the latent association status distinguishes between an association being null or non-null, we set *H*_0_: *z*_1_ = … = *z*_*K*_ = 0 (*Z* = 0) versus *H*_1_: at least one of *z*_1_, …, *z*_*K*_ = 1 (*Z* ≠ 0).

#### Local false discovery rate (locFDR)/Posterior probability of null association (PPNA)

Let *D* denote the summary statistics data at a SNP across traits. We consider the local false discovery rate (locFDR = *P*(*H*_0_|*D*)) [26, 27] as a measure for evaluating the aggregate-level pleiotropic association. The posterior odds (PO) of *H*_1_ versus *H*_0_ is $\frac{P(H_{1}|D)}{P(H_{0}|D)}$. Stephens and Balding [38] introduced the notion of the posterior probability of association (PPA) defined as: *PPA* = *PO*/(1 + *PO*). We define the posterior probability of null association (PPNA) = 1 − *PPA*. Note that PPNA = 1/(1+PO) = *P*(*H*_0_|*D*). Hence, by definition, locFDR and PPNA are the same quantity. It can be viewed as a Bayesian analog of the p-value [38]. If the data supports *H*_1_, locFDR should be close to zero, and if the data supports *H*_0_, it should be close to one (similar to a p-value). We estimate locFDR = *P*(*H*_0_|*D*) = *P*(*Z* = 0|*D*) based on the MCMC posterior sample. Note that *P*(*Z* = 0|*D*) = ∫ ∫ ∫ *P*(*Z* = 0|***β***, *q*, *d*, *D*)*f*(***β***, *q*, *d*|*D*)*d*(***β***)*d*(*q*)*d*(*d*). Thus,

$$P(Z=0|D)=E_{\beta ,q,d|D}P(Z=0|\beta ,q,d,D)\approx \frac{1}{N}\sum_{i=1}^{N}P(Z=0|\beta^{\left(i\right)},q^{\left(i\right)},d^{\left(i\right)},D),$$ (3)

where (***β***^(*i*)^, *q*^(*i*)^, *d*^(*i*)^) denotes the *i*^*th*^ posterior sample of (***β***, *q*, *d*) obtained by the MCMC. We note that the full conditional posterior distributions of *z*_1_, …, *z*_*K*_ are independent (see step 5 in Algorithm 1 and the derivation of full conditional distributions of *z*_1_, …, *z*_*K*_ in the supporting information (S1 Text)). Hence, $P\left(Z=0|\beta^{\left(i\right)},q^{\left(i\right)},d^{\left(i\right)},D\right)=\prod_{j=1}^{K}P\left(z_{j}=0|\beta^{\left(i\right)},q^{\left(i\right)},d^{\left(i\right)},D\right)$. This independence property of the full conditional posterior distributions of *z*_1_, …, *z*_*K*_ is crucial for the explicit estimation of the locFDR.

#### Bayes factor (BF)

The Bayes Factor for testing *H*_1_ against *H*_0_ is given by:

$$\text{BF}=\frac{P(D|H_{1})}{P(D|H_{0})}=\frac{P(H_{1}|D)}{P(H_{0}|D)}\frac{P(H_{0})}{P(H_{1})}=\frac{P(Z\neq 0|D)}{P(Z=0|D)}\frac{P(Z=0)}{P(Z\neq 0)}=\frac{\text{Posterior}\;\text{odds}}{\text{Prior}\;\text{odds}}$$ (4)

The posterior odds of *H*_1_ vs. $H_{0}=\frac{P(Z\neq 0|D)}{P(Z=0|D)}=\frac{1-P(Z=0|D)}{P(Z=0|D)}$ and the prior odds of *H*_1_ vs. $H_{0}=\frac{P(Z\neq 0)}{P(Z=0)}=\frac{1-P(Z=0)}{P(Z=0)}$. Of note, $P\left(z_{j}=1\right)=E\left(q\right)=\frac{c_{1}}{c_{1}+c_{2}}=p_{1}$. Let *p*_0_ = 1 − *p*_1_. Since, *z*_*j*_s are independently distributed in the prior, $P\left(Z=0\right)=p_{0}^{K}$, and $P\left(Z\neq 0\right)=1-p_{0}^{K}$. So the prior odds = $\frac{1-p_{0}^{K}}{p_{0}^{K}}$. Of note, *P*(*Z* = 0|*D*) is the locFDR.

#### Selection of optimal subset of associated traits

For *i* = 1, …, *N*, let $S_{i}=\left\{Y_{j}:z_{j}^{\left(i\right)}=1;\;j=1,\ldots ,K\right\}$ denote the subset of associated traits detected in the *i*^*th*^ MCMC posterior sample. That subset of traits which is observed with the maximum frequency in the posterior sample is estimated as the optimal subset of associated traits. It is the maximum a posteriori (MAP) estimate of the optimal subset.

Let PPA_*j*_ denote the marginal trait-specific posterior probability of association which is estimated as $\frac{1}{N}\sum_{i=1}^{N}z_{j}^{\left(i\right)}$ for the phenotype *Y*_*j*_. PPA_*j*_ provides a better insight into a pleiotropic signal. It quantifies the relative contribution of the traits underlying a pleiotropic signal. Even if a trait is not selected in the MAP estimate of the optimal subset of non-null traits, the estimated PPA_*j*_ for the trait may not be negligible, e.g., 20%. An interpretation of such a phenomenon is that even though the estimated genetic effect on a phenotype was not substantial enough to make into the optimal subset, the possibility of the genetic variant having a pleiotropic effect on the trait along with those in the optimal subset seems promising. One can also estimate the joint posterior probability of a particular subset of traits being associated based on the posterior sample of *Z*. For example, for two traits, it may be of interest to estimate the joint posterior probability that the first trait is associated but the second trait is not [P(*z*_1_ = 1, *z*_2_ = 0|*D*)]. This probability can be estimated as the proportion of posterior samples in which *z*_1_ = 1 and *z*_2_ = 0.

The direction of association between each non-null trait and a genetic variant can be estimated based on the posterior sample of ***β***. The posterior probability that *Y*_*j*_ is positively associated is estimated as the proportion of positive *β*_*j*_ among the posterior sample of *β*_*j*_. *Y*_*j*_ is classified as being positively associated if this estimated proportion is greater than half. The posterior mean, median, and the 95% credible interval (Bayesian analog of the frequentist confidence interval) of the true genetic effect on each phenotype can be computed based on the posterior sample of ***β***.

### Specifying the hyperparameters

#### Variance of the spike and slab distributions

After extensive experimentation with simulated data, we set the variance of the spike distribution *τ*^2^ (variance of null or very weak genetic effects across traits) to a fixed value 10^−4^. If *β* (log odds ratio) follows *N*(0, 10^−4^), then *P*(0.98 < *e*^*β*^ < 1.02) = 0.954. It implies that under the spike (no association), the odds ratio for association between a variant and single trait will vary between 0.98 and 1.02 with a prior probability of 95.4%. In the MCMC, we updated the slab variance ${\left(\frac{\tau }{d}\right)}^{2}$ in the range (0.6 − 1.0) with the median value equal to 0.8. If *β* ∼ *N*(0, 0.8), then *P*(*e*^*β*^ < 0.98 or *e*^*β*^ > 1.02) = 0.99, which implies that under the slab (association) with variance 0.8, the odds ratio is smaller than 0.98 (a negative association) or larger than 1.02 (a positive association) with a prior probability of 99%. We note that a majority of the odds ratios reported for the GW significant associations with various complex traits are modest in size [39]. Hence, it is desirable to place a substantial prior probability in modest ranges of non-null odds ratios, e.g. (1.02 − 1.5) ∪ (1/1.5 − 1/1.02), (1.02 − 2.0) ∪ (1/2.0 − 1/1.02). In S1 Table, we provide the prior probability of different ranges of non-null odds ratio induced by three different choices of the slab variance as 0.6, 0.8 and 1.0, respectively. For example, when the slab variance is 0.6, P(1.02 <OR <1.5) + P(1/1.5 <OR <1/1.02) = 0.38 and P(1.02 <OR <2.0) + P(1/2.0 <OR <1/1.02) = 0.61.

We also explored other choices for these parameters by simulations, such as, *τ*^2^ = 10^−3^, 10^−2^, and ${\left(\frac{\tau }{d}\right)}^{2}$ in a range (0.5 − 1.0), (0.7 − 1.1), etc. The values used here [*τ*^2^ = 10^−4^, ${\left(\frac{\tau }{d}\right)}^{2}\in \left(0.6-1.0\right)]$ controlled the realized FDR while evaluating the aggregate-level pleiotropic association and gave an overall high level of specificity and an overall good level of sensitivity while selecting the optimal subset of associated traits across a wide range of scenarios. The choice of the spike variance and the ratio of the slab and spike variances ($\frac{1}{d^{2}}$) directly impact the selection accuracy [19]. A smaller choice of the slab variance will increase sensitivity of CPBayes, but at the expense of decreased specificity.

#### Shape parameters of the prior distribution of *q*

In the continuous spike and slab prior, *q* ∼ Beta(*c*_1_, *c*_2_), *c*_1_ > 0, *c*_2_ > 0; $E\left(q\right)=\frac{c_{1}}{c_{1}+c_{2}}$. If *c*_1_ = *c*_2_ = 1, $E\left(q\right)=\frac{1}{2}$, which implies that half of the traits are expected to be associated with a SNP in the prior. However in GWAS, a small proportion of variants are genome-wide significantly associated with complex traits. For the sake of convenience, we fix *c*_2_ as 1. Choosing a smaller value of *c*_1_ than *c*_2_ induces a small value of *E*(*q*) which is appropriate for null SNPs but can severely penalize the power of CPBayes to detect the risk SNPs. Hence we consider a simple Empirical Bayes approach here. First, apply BH_0.01_ to the univariate association p-values for a SNP across traits and let $\hat{q}$ be the proportion of associated traits detected by BH_0.01_. If $\hat{q}<0.1$, we set $\hat{q}=0.1$; if $\hat{q}>0.5$, we set $\hat{q}=0.5$. Next, we consider $E\left(q\right)=\frac{c_{1}}{c_{1}+c_{2}}=\hat{q}$ and calculate *c*_1_ fixing *c*_2_ = 1. As we restrict $0.1<\hat{q}<0.5$ and *c*_2_ = 1, *c*_1_ varies from $\frac{1}{9}$ to 1 depending on $\hat{q}$. We note that even though Majumdar et al. [31] demonstrated that BH_0.01_ provides good specificity and sensitivity while selecting non-null traits underlying a pleiotropic signal, it may not always be accurate. Hence in the prior, we do not allow *c*_1_ > 1 $\left(\hat{q}>\frac{1}{2}\right)$. Of note, *c*_1_ = *c*_2_ = 1 corresponds to the Uniform(0, 1) distribution. On the other hand, allowing $\hat{q}<0.1$ can induce a too small value of *c*_1_ which can cause computational issues in the MCMC due to generating too small value of *q*, and it can also severely affect CPBayes’ power of detecting risk variants and the sensitivity of selecting the non-null traits underlying a pleiotropic signal. We have observed in our simulation study that CPBayes performs well overall by using this Empirical Bayes approach to choose the hyperparameters in the prior of *q*.

### Estimating the correlation between summary statistics

The summary statistics across traits can be correlated due to overlap or close genetic relatedness among subjects across different studies. For case-control studies, Zaykin and Kozbur [40] and Lin and Sullivan [41] derived a simple formula of correlation among ${\hat{\beta }}_{1},\ldots ,{\hat{\beta }}_{K}$. For *k*, *l* ∈ {1, …, *K*} and *k* ≠ *l*,

$$\begin{array}{c} corr\left({\hat{\beta }}_{k},{\hat{\beta }}_{l}\right)=(n_{kl}^{\left(11\right)}\sqrt{\frac{n_{k}^{\left(0\right)}n_{l}^{\left(0\right)}}{n_{k}^{\left(1\right)}n_{l}^{\left(1\right)}}}+n_{kl}^{\left(00\right)}\sqrt{\frac{n_{k}^{\left(1\right)}n_{l}^{\left(1\right)}}{n_{k}^{\left(0\right)}n_{l}^{\left(0\right)}}})/\sqrt{n_{k}n_{l}} \end{array}$$ (5)

Here $n_{k}^{\left(1\right)}$, $n_{k}^{\left(0\right)}$, and *n*_*k*_ (or $n_{l}^{\left(1\right)}$, $n_{l}^{\left(0\right)}$, and *n*_*l*_) denote the number of cases, controls, and total sample size for the study of *Y*_*k*_ (or *Y*_*l*_); $n_{kl}^{\left(11\right)}$ and $n_{kl}^{\left(00\right)}$ denote the number of cases and controls shared between the studies of *Y*_*k*_ and *Y*_*l*_. Let $n_{kl}^{\left(10\right)}$ be the number of overlapping subjects that are cases for *Y*_*k*_ but controls for *Y*_*l*_; similarly, let $n_{kl}^{\left(01\right)}$ be the number of shared subjects that are controls for *Y*_*k*_ but cases for *Y*_*l*_. Here, the above formula can be generalized to:

$$corr({\hat{\beta }}_{k},{\hat{\beta }}_{l})=(n_{kl}^{\left(11\right)}\sqrt{\frac{n_{k}^{\left(0\right)}n_{l}^{\left(0\right)}}{n_{k}^{\left(1\right)}n_{l}^{\left(1\right)}}}-n_{kl}^{\left(10\right)}\sqrt{\frac{n_{k}^{\left(0\right)}n_{l}^{\left(1\right)}}{n_{k}^{\left(1\right)}n_{l}^{\left(0\right)}}}-n_{kl}^{\left(01\right)}\sqrt{\frac{n_{k}^{\left(1\right)}n_{l}^{\left(0\right)}}{n_{k}^{\left(0\right)}n_{l}^{\left(1\right)}}}+n_{kl}^{\left(00\right)}\sqrt{\frac{n_{k}^{\left(1\right)}n_{l}^{\left(1\right)}}{n_{k}^{\left(0\right)}n_{l}^{\left(0\right)}}})/\sqrt{n_{k}n_{l}}$$ (6)

This formula is accurate when none of the phenotypes *Y*_1_, …, *Y*_*K*_ is associated with the SNP and environmental covariates. An alternative strategy [5, 34] is based on using GW (genome-wide) summary statistics data to estimate the correlation structure, which is useful when the environmental covariates are associated with the phenotypes. For continuous and normally distributed traits, the correlation matrix of effect estimates under the null is the phenotypic correlation matrix. But, its calculation requires individual level phenotype data across multiple traits. For general type of traits (e.g. non-normal continuous traits, count phenotypes), a standard formula of correlation between the effect estimates may be difficult to derive. Such a formula may also require information only available from individual-level phenotype data. For example, the effect estimates’ correlation formula for binary traits requires the number of cases, controls in each study and the number of overlapping cases and controls between studies. In such scenarios, since the genome-wide (GW) effect estimates corresponding to multiple traits will be available in the pleiotropy analysis, the GW summary statistics based approach can be applied irrespective of the type of traits without requiring any individual-level phenotype data. So, the GW summary statistics based approach is useful to estimate the correlation structure of effect estimates in various scenarios including correlated non-binary traits.

### A combined strategy for correlated summary statistics

For strongly correlated summary statistics, when a majority of the traits are associated with the risk locus (non-sparse scenario), the Gibbs sampler can sometimes be trapped in a local mode rather than the global mode of the posterior distribution due to possible multi-modality of the posterior distribution of model parameters. We observed this pattern in our simulation study. It may result in an incorrect selection of associated traits, reducing the robustness of CPBayes. We noticed that, in such a scenario, if the summary statistics are assumed to be uncorrelated, the MCMC does not get trapped in a local mode and moves around the global mode. But ignoring the correlation can give a lower (larger) Bayes factor (locFDR) and sensitivity of the selected traits. Hence, for correlated summary statistics, we combine the correlated and the uncorrelated versions of CPBayes as follows. First, we execute CPBayes considering the correlation among ${\hat{\beta }}_{1},\ldots ,{\hat{\beta }}_{K}$. Let *A* denote the selected subset of non-null traits that contains *K*_1_ traits. Let *B* denote the subset of *K*_1_ traits that have the smallest univariate association p-values. If *A* and *B* match, we accept the results; otherwise, we implement CPBayes assuming that ${\hat{\beta }}_{1},\ldots ,{\hat{\beta }}_{K}$ are uncorrelated and accept the results obtained. Note that, if *A* is empty, we accept the results provided by the correlated version of CPBayes. In this combined strategy, we induce a frequentist sense of selection. Because, majority of the multiple testing procedures reject the null hypotheses for which smallest univariate p-values are obtained. However, we note that in the analysis of the GERA cohort, the combined strategy used the uncorrelated version very few times. The reason is that the non-sparse scenario may not occur frequently in real data.

### Theoretical comparison between the continuous and Dirac spike

It is straightforward to observe that the Dirac spike can be obtained from the continuous spike by first setting *τ* = 0 and $\frac{\tau }{d}=b$ in Eq 1, and then integrating out the latent variables *Z* from the model. We note that, the latent association status (*Z*) could only be used in the model for the continuous spike. For the Dirac spike, the inclusion of *Z* in the model makes the corresponding MCMC reducible, and hence non-convergent to its stationary distribution (details not provided). Also, for the continuous spike, the full conditional posterior distributions of *z*_1_, …, *z*_*K*_ are independent which leads to an explicit estimation of the locFDR/Bayes factor based on the MCMC sample. But, for the Dirac spike, the explicit estimation of the locFDR/Bayes factor appears to be very difficult in the correlated case, because the full conditional posterior distributions of *β*_1_, …, *β*_*K*_ are not independent for correlated summary statistics.

### ASSET

Bhattacharjee et al. [7] introduced an elegant subset-based meta analysis method ASSET to analyze pleiotropy. While regressing *k*^*th*^ phenotype *Y*_*k*_ on genotype *G*, let ${\hat{\beta }}_{k},s_{k}$ be the estimates of the association parameter and its standard error, *k* = 1, …, *K*. Adopting the framework of a fixed-effects meta analysis, for a subset of traits A, ASSET defines the *Z* statistic as: $Z\left(A\right)=\sum_{k:Y_{k}\in A}\sqrt{\pi_{k}\left(A\right)}Z_{k}$, where $Z_{k}=\frac{{\hat{\beta }}_{k}}{s_{k}}$, and $\pi_{k}\left(A\right)$ is an appropriate weight associated with *Y*_*k*_ belonging to A. For example, if there are *K* separate GWAS for *Y*_1_, …, *Y*_*K*_ with the *k*^*th*^ study having a sample size *n*_*k*_, one can consider $\pi_{k}\left(A\right)=\frac{n_{k}}{\sum_{k\in A}n_{k}}$. The global association of a SNP with at least one trait is measured by the test-statistic: $Z_{max}=max_{A}\left|Z\left(A\right)\right|$, where the maximization is taken across all possible A. In addition to the p-value of global association, ASSET also offers an optimal subset of non-null traits that are associated with the SNP, which is essentially the subset of traits that constructs *Z*_*max*_. For more details, see Bhattacharjee et al. [7].

### Benjamini Hochberg FDR controlling procedure

Benjamini and Hochberg [30] introduced a sequential procedure that controls the expected FDR in multiple hypothesis testing. Majumdar et al. [31] demonstrated that the BH procedure is a simple but efficient strategy to select non-null traits underlying a pleiotropic signal. For each individual risk SNP associated with at least one trait, we applied the BH procedure to the univariate association p-values for the phenotypes under consideration with the level of FDR as 0.01 which was suggested by Majumdar et al. [31]. We refer it as BH_0.01_.

### Gibbs sampling algorithm for continuous spike

Here we state the Gibbs sampling algorithm for the continuous spike described in Eq 1. It is a desirable practice to provide the MCMC with a good initial value of the model parameters for faster convergence to its stationary distribution. Hence, we apply BH_0.01_ on the univariate association p-values of *K* traits and assign *z*_*j*_ = 1 if *Y*_*j*_ is found to be significantly associated, otherwise set *z*_*j*_ = 0; *j* = 1, …, *K*. We also choose an initial value of *q* as the proportion of non-null traits detected by BH_0.01_ (the boundary situations of no/all non-null traits are taken care of appropriately).

Define $\Sigma_{2}=diag\left(\tau_{1}^{2},\ldots ,\tau_{K}^{2}\right)$ (a diagonal matrix with diagonal elements $\tau_{1}^{2},\ldots ,\tau_{K}^{2}$), where *τ*_*j*_ = *τ* if *z*_*j*_ = 0; and $\tau_{j}=\frac{\tau }{d}$ if *z*_*j*_ = 1; *j* = 1, …, *K*. So $\beta |Z∼\text{MVN}(\underset{˜}{0},\Sigma_{2})$. Let Σ_1_ = *S*. Also, let ***β***_−*j*_ = (*β*_1_, …, *β*_*j*−1_, *β*_*j*+1_, …, *β*_*K*_), and *Z*_−*j*_ = (*z*_1_, …, *z*_*j*−1_, *z*_*j*+1_, …, *z*_*K*_).

**Algorithm 1** Gibbs sampling for continuous spike in correlated case

1: *Start*:

2: Assign the initial values of *Z* and *q* as described above.

3: *loop*:

4: Simulate ***β*** from its full conditional posterior distribution: $\beta |Z,q,d,\hat{\beta }∼\text{MVN}\left[{\left(\Sigma_{1}^{-1}+\Sigma_{2}^{-1}\right)}^{-1}\Sigma_{1}^{-1}\hat{\beta },{\left(\Sigma_{1}^{-1}+\Sigma_{2}^{-1}\right)}^{-1}\right]$.

5: For *j* = 1, …, *K*, update *z*_*j*_ using the full conditional posterior probability: $P\left(z_{j}=0|Z_{-j},\beta ,q,d,\hat{\beta }\right)=\frac{1}{1+{\text{ratio}}_{j}}$, where ${\text{ratio}}_{j}=\frac{q}{1-q}\;d\;\text{exp}\left[-\frac{\beta_{j}^{2}}{2\tau^{2}}\left(d^{2}-1\right)\right]$.

6: Let $k_{1}=\sum_{j=1}^{K}z_{j},$ *k*_0_ = *K* − *k*_1_. Update *q* using $q|\beta ,Z,d,\hat{\beta }∼\text{Beta}\left(c_{1}+k_{1},c_{2}+k_{0}\right)$.

7: We assume that *e*_1_ = *e*_2_ = 1. Update *d* from its full conditional posterior distribution in a fixed range so that the slab variance ${\left(\frac{\tau }{d}\right)}^{2}$ varies in a given range (*v*_0_, *v*_1_); let the corresponding range of *d* be given by: *d*_0_ < *d* < *d*_1_. If $k_{1}=\sum_{j=1}^{K}z_{j}>0$, then $d=\sqrt{\frac{y}{2C}}$, where $C=\frac{1}{2\tau^{2}}\sum_{j:z_{j}=1}\beta_{j}^{2}$, and *y* follows a truncated $\left(2Cd_{0}^{2}<y<2Cd_{1}^{2}\right)$ central $\chi_{k_{1}+1}^{2}$ distribution. If *k*_1_ = 0, *d* is updated from the truncated (*d*_0_ < *d* < *d*_1_) Beta(1, 1) distribution.

8: **goto** *loop* until all the MCMC iterations are finished.

We note that, *d* can be updated using the truncated central *χ*^2^ distribution as long as the second shape parameter of its Beta prior (*e*_2_) is 1.

If the summary statistics are uncorrelated, step 4 of Algorithm 1 is modified as: for *j* = 1, …, *K*, update *β*_*j*_ by sampling from its full conditional posterior distribution: $\beta_{j}|\beta_{-j},Z,q,d,\hat{\beta }∼\text{N}\left(\frac{\sigma_{j}^{2}}{s_{j}^{2}}{\hat{\beta }}_{j},\sigma_{j}^{2}\right)$, where $\frac{1}{\sigma_{j}^{2}}=\frac{1}{s_{j}^{2}}+\frac{1}{\tau_{j}^{2}}$. All the other steps remain the same as in the Algorithm 1.

## Supporting information

S1 Text This document contains outline of mathematical derivation of the full conditional posterior distributions for continuous spike, Gibbs sampling algorithm for Dirac spike, outline of mathematical derivation of the full conditional posterior distributions for Dirac spike, simulation model to generate phenotype data in cohort study with binary traits, comparison between CPBayes and GPA, summary of the measures of overall pleiotropic association obtained while evaluating selection accuracy, simulation results for 50 traits, comparison between continuous spike and Dirac spike, assessment of different SNP filtering thresholds to estimate the effect estimates’ correlation structure in GERA cohort. (PDF)

S1 Fig An example diagram of the continuous spike and slab prior used by CPBayes to model pleiotropy. In this diagram, the spike variance is chosen as 0.1.
(PDF)

S2 Fig A diagram presenting the continuous spike and slab prior modeling pleiotropy with the spike variance *τ*^2^ = 10^−4^. (PDF)

S3 FigSelection accuracy of different methods for cohort study.The total number of phenotypes is denoted by *K* and *m* denotes the minor allele frequency at the risk SNP. $K_{1}^{+}$ and $K_{1}^{-}$ denote the number of positively and negatively associated traits, respectively. Two different colors for each method present two scenarios: 1. all non-null effects are positive (++), 2. non-null effects are both positive and negative (+−).(PDF)

S4 FigSelection accuracy of different methods for 15 non-overlapping case-control studies.The total number of studies is denoted by *K* and *m* denotes the minor allele frequency at the risk SNP. $K_{1}^{+}$ and $K_{1}^{-}$ denote the number of positively and negatively associated traits, respectively. Two different colors for each method present two scenarios: 1. all non-null effects are positive (++), 2. non-null effects are both positive and negative (+−).(PDF)

S5 Fig Selection accuracy of different methods for 15 overlapping case-control studies. (PDF)

S6 Fig Estimated joint posterior probabilities of the association configurations obtained by CPBayes and GPA for first 10 null SNPs. Here 1% of 1000 SNPs are risk SNPs and associated only with the second trait, and 99% SNPs are null.
(PDF)

S7 Fig Estimated joint posterior probabilities of the association configurations obtained by CPBayes and GPA for the risk SNPs. Here 1% of 1000 SNPs are risk SNPs and associated only with the second trait, and 99% SNPs are null.
(PDF)

S8 Fig Estimated joint posterior probabilities of the association configurations obtained by CPBayes and GPA for the risk SNPs. Here 1% of 1000 SNPs are risk SNPs and associated with both the traits, and 99% SNPs are null.
(PDF)

S9 Fig Estimated joint posterior probabilities of the association configurations obtained by CPBayes and GPA for the first 10 risk SNPs. Here 2% of 1000 SNPs are risk SNPs and associated only with the second trait, and 98% SNPs are null.
(PDF)

S10 Fig Comparison of the accuracy of selection of associated traits by the continuous and Dirac spike for multiple overlapping case-control studies. (PDF)

S11 Fig Forest plot for pleiotropic signal at rs6025 detected by CPBayes. (PDF)

S12 Fig Forest plot for pleiotropic signal at rs13211628 detected by CPBayes. (PDF)

S13 Fig Forest plot for pleiotropic signal at rs10455872 detected by CPBayes. (PDF)

S14 Fig Forest plot for pleiotropic signal at rs3957148 detected by CPBayes. (PDF)

S15 Fig Forest plot for pleiotropic signal at rs687289 detected by CPBayes. (PDF)

S1 Table Prior probabilities of various ranges of odds ratio (OR) under different choices of the slab variance in the continuous spike and slab prior. (PDF)

S2 Table Comparison between main theoretical features of CPBayes and ASSET. (PDF)

S3 Table Summary of measures of the overall pleiotropic association under the global null hypothesis of no association when multiple case-control studies with overlapping subjects are considered. (PDF)

S4 Table Summary of measures of the evidence of overall pleiotropic association when a subset of traits are associated for 10 overlapping case-control studies. Here 2 and 4 among 10 traits are associated.
(PDF)

S5 Table Summary of measures of the evidence of overall pleiotropic association when a subset of traits are associated for 10 overlapping case-control studies. Here 6 and 8 among 10 traits are associated.
(PDF)

S6 Table Simulation study for 50 traits. Summary of measures for the evidence of the overall pleiotropic association for 50 non-overlapping case-control studies. Here 0, 5, and 10 among 50 traits are associated.
(PDF)

S7 Table Simulation study for 50 traits. Accuracy in selection of associated traits by CPBayes and BH_0.01_ for 50 case-control studies.
(PDF)

S8 Table Name of 22 phenotypes in the GERA cohort analyzed by CPBayes and ASSET. (PDF)

S9 Table Distance between estimated correlation matrices of effect estimates in the GERA cohort obtained by using different thresholds of the minimum of univariate association p-value across traits and *r*^2^ value between a pair of SNPs to select independent null SNPs. At the beginning of the table, the number of independent null SNPs obtained by using different SNP filtering thresholds are listed. At the bottom of the table, we also provide the distance between the correlation matrix estimated by Eq 6 based on the number of overlapping cases and controls and the GW summary statistics based approach.
(PDF)

S10 Table Independent pleiotropic SNPs identified by CPBayes for which one phenotype was selected. (PDF)

S11 Table Independent pleiotropic signals on chromosome 1-2 detected by ASSET. (PDF)
