# Supplementary material for: An efficient Bayesian meta-analysis approach for studying cross-phenotype genetic associations
Source: PLoS Genet. 2018 Feb 12;14(2):e1007139. doi: 10.1371/journal.pgen.1007139 (PMC5825176; doi:10.1371/journal.pgen.1007139)
Supplement: S1 Text — (PDF) [file pgen.1007139.s001.pdf]

Supporting information: An efficient Bayesian meta-analysis approach for studying cross-phenotype genetic associations

Arunabha Majumdar[1], Tanushree Haldar[2], Sourabh Bhattacharya[3], John S. Witte[1]

[1] Department of Epidemiology and Biostatistics, University of California, San Francisco

[2] Institute for Human Genetics, University of California, San Francisco

[3] Interdisciplinary Statistical Research Unit, Indian Statistical Institute, Kolkata

S1 Text

1 Outline of mathematical derivation of the full conditional posterior distributions for continuous spike

1.1 Correlated case

Here we derive the full conditional posterior distributions of the model parameters to perform Gibbs sampling for correlated summary statistics. If S is the covariance matrix of $\hat{\boldsymbol{\beta}}$,

$$\hat{\boldsymbol{\beta}}|\boldsymbol{\beta}, S \sim \text{MVN}(\boldsymbol{\beta}, S).$$

For $j = 1, \ldots, K$,

$$\begin{aligned}
&\beta_j|z_j, \tau, d \overset{ind}{\sim} (1-z_j)\ N(0,\tau^2) + z_j\ N(0, \left(\frac{\tau}{d}\right)^2); \quad \tau > 0,\ 0 < d < 1,\ \left(\frac{\tau}{d}\right)^2 > \tau^2 \\
&P(z_j = 1|q) = q; \quad P(z_j = 0|q) = (1-q);\ 0 < q < 1 \\
&q|c_1, c_2 \sim \text{Beta}(c_1, c_2) \Rightarrow P(z_j = 1) = E(q) = \frac{c_1}{c_1 + c_2} \\
&d|e_1, e_2 \sim \text{Beta}(e_1, e_2)
\end{aligned} \tag{7}$$

So, $\beta_j|z_j = 0, \tau \sim \text{N}(0, \tau^2)$ and $\beta_j|z_j = 1, \tau, d \sim \text{N}(0, (\frac{\tau}{d})^2)$. Let $\Sigma_1 = S$. Define, $\Sigma_2 = diag(\tau_1^2, \ldots, \tau_K^2)$ (a diagonal matrix with the diagonal elements $\tau_1^2, \ldots, \tau_K^2$), where $\tau_j = \tau$ if $z_j = 0$, and $\tau_j = \frac{\tau}{d}$ if $z_j = 1$, $j = 1, \ldots, K$. So,

$$\boldsymbol{\beta}|Z, \tau, d \sim \text{MVN}(\underset{\sim}{0}, \Sigma_2), \tag{8}$$

1.1.1 Full conditional posterior distribution of $\boldsymbol{\beta}$

Let $[U]$ denote a generic notation of the probability distribution of a random variable U, and $[U_1|U_2]$ denote a generic notation of the conditional probability distribution of U_1 given U_2. We have considered fixed choice of τ. Hence we drop it from the set of conditional parameters while writing the expressions of the full conditional distributions. For example, we write $[\boldsymbol{\beta}|Z, q, d, \hat{\boldsymbol{\beta}}]$ instead of $[\boldsymbol{\beta}|Z, q, \tau, d, \hat{\boldsymbol{\beta}}]$. Note that,

$$[\boldsymbol{\beta}|Z, q, d, \hat{\boldsymbol{\beta}}] \propto [\hat{\boldsymbol{\beta}}, \boldsymbol{\beta}, Z, q, d] \propto [\hat{\boldsymbol{\beta}}|\boldsymbol{\beta}]\ [\boldsymbol{\beta}|Z, d] \tag{9}$$

Applying standard techniques from linear algebra and distribution theory of multivariate normal, one can obtain that:

$$\boldsymbol{\beta}|Z, q, d, \hat{\boldsymbol{\beta}} \sim \text{MVN}[(\Sigma_1^{-1} + \Sigma_2^{-1})^{-1}\Sigma_1^{-1}\hat{\boldsymbol{\beta}}, (\Sigma_1^{-1} + \Sigma_2^{-1})^{-1}] \tag{10}$$

Note that Σ_2 is dependent on Z, and Z influences the full conditional posterior distribution of $\boldsymbol{\beta}$ through the specification of Σ_2.

1.1.2 Full conditional posterior distribution of Z

Let $Z_{-j} = (z_1, \ldots, z_{j-1}, z_{j+1}, \ldots, z_K)$. Note that, $P(z_j = 0|Z_{-j}, \boldsymbol{\beta}, q, d, \hat{\boldsymbol{\beta}}) \propto [\beta_j|z_j = 0]\ [z_j = 0|q]$. Similarly, $P(z_j = 1|Z_{-j}, \boldsymbol{\beta}, q, d, \hat{\boldsymbol{\beta}}) \propto [\beta_j|z_j = 1, d]\ [z_j = 1|q]$. Combining these two equations, we obtain that:

$$P(z_j = 0|Z_{-j}, \boldsymbol{\beta}, q, d, \hat{\boldsymbol{\beta}}) = \frac{1}{1 + \text{ratio}_j}, \text{ where ratio}_j = \frac{q}{1-q}\ d \exp[-\frac{\beta_j^2}{2\tau^2}\ (d^2 - 1)] \tag{11}$$

We note that the full conditional posterior distribution of z_j does not depend on Z_{-j}. Hence, the full conditional distributions of $z_1, \ldots, z_K$ are independent.

1.1.3 Full conditional posterior distribution of q

Note that, $[q|\boldsymbol{\beta}, Z, d, \hat{\boldsymbol{\beta}}] \propto [Z|q]\ [q] \propto \prod_{j=1}^{K}[z_j|q]\ [q]$. Let $k_1 = \sum_{j=1}^{K} z_j$, and $k_0 = K - k_1$. Then it can easily be derived that:

$$q|\boldsymbol{\beta}, Z, d, \hat{\boldsymbol{\beta}} \sim \text{Beta}(c_1 + k_1, c_2 + k_0) \tag{12}$$

1.1.4 Full conditional posterior distribution of d

We assume that $e_2 = 1$ and derive a closed-form full conditional posterior distribution of d under this restriction. Let $\sum_{j:z_j=1} z_j = k_1$.

<u>Case 1</u>: $k_1 > 0$

Of note, $[d|\boldsymbol{\beta}, Z, q, \hat{\boldsymbol{\beta}}] \propto \prod_{j:z_j=1}[\beta_j|z_j = 1, d]\ [d] \propto \exp[-C \times d^2] \times d^{(k_1+e_1)-1}$, where $C = \frac{1}{2\tau^2}\sum_{j:z_j=1}\beta_j^2$. We consider the following transformation of variable: $y = 2Cd^2$. It can be derived that: $y \sim \chi^2_{k_1+e_1}$ under the assumption that $y > 0$.

Suppose that, we want to update the slab variance $(\frac{\tau}{d})^2$ in a range (say, $v_0 - v_1$) such that the corresponding range of d is given by: $d_0 < d < d_1$. Using the above transformation of variable, the corresponding range of y is given by: $2Cd_0^2 < y < 2Cd_1^2$. Hence, $y \sim$ truncated central $\chi^2_{k_1+e_1}$, where $2Cd_0^2 < y < 2Cd_1^2$. Finally, the updated d can be obtained by using the transformation $d = \sqrt{\frac{y}{2C}}$.

<u>Case 2</u>: $k_1 = 0$

It can easily be shown that: $d|\boldsymbol{\beta}, Z, q, \hat{\boldsymbol{\beta}} \sim$ truncated Beta$(e_1, 1)$, where $d_0 < d < d_1$.

In the Algorithm 1 in main text, we considered $e_1 = 1$, which is a natural choice under the absence of any prior information.

1.2 Uncorrelated case

When the summary statistics are uncorrelated, the full conditional posterior distributions of all the parameters except $\boldsymbol{\beta}$ remain the same as in the correlated case (described above). Now the full conditional distributions of $\beta_1, \ldots, \beta_K$ become independent. For $j = 1, \ldots, K$,

$$\hat{\beta}_j|\beta_j \overset{ind}{\sim} \mathrm{N}(\beta_j, s_j^2), \text{ and } [\beta_j|\boldsymbol{\beta}_{-j}, Z, q, d, \hat{\boldsymbol{\beta}}] \propto [\hat{\beta}_j|\beta_j] \times [\beta_j|z_j, d] \tag{13}$$

Using the above equation, it's easy to derive the full conditional distribution as: for $j = 1, \ldots, K$, $\beta_j|\boldsymbol{\beta}_{-j}, Z, q, d, \hat{\boldsymbol{\beta}} \overset{ind}{\sim} \mathrm{N}(\frac{\sigma_j^2}{s_j^2}\hat{\beta}_j, \sigma_j^2)$, where $\frac{1}{\sigma_j^2} = \frac{1}{s_j^2} + \frac{1}{\tau_j^2}$.

2 Gibbs sampling algorithm for Dirac spike

Here we outline the Gibbs sampler for the Dirac spike. We apply $\mathrm{BH}_{0.01}$ on the univariate association p-values of K traits and assign $\beta_j = \hat{\beta}_j$ (since $\hat{\beta}_j$ is a consistent estimator of β_j) if Y_j is found to be associated, otherwise we set $\beta_j = 0$; $j = 1, \ldots, K$. We also choose an initial value of q as the proportion of non-null/associated traits detected by $\mathrm{BH}_{0.01}$ (the boundary situations of no/all non-null traits are taken care of appropriately).

Let $\boldsymbol{\beta}_{-j} = \{\beta_1, \ldots, \beta_{j-1}, \beta_{j+1}, \ldots, \beta_K\}$. Consider the following partition of S:

$$S = \begin{bmatrix} s_j^2 & S_{j,-j} \\ S_{-j,j} & S_{-j,-j} \end{bmatrix}$$

Let $\bar{\sigma}_j^2 = s_j^2 - (S_{j,-j} \times S_{-j,-j}^{-1} \times S_{-j,j})$, and $m_{j,-j} = \hat{\beta}_j - (S_{j,-j} \times S_{-j,-j}^{-1} \times (\hat{\boldsymbol{\beta}}_{-j} - \boldsymbol{\beta}_{-j}))$.

Algorithm S1 Gibbs sampling for the Dirac spike for correlated summary statistics

1: *Start*:

2: Assign the initial values of $\boldsymbol{\beta}$ and q as discussed above.

3: *loop*:

4: For $j = 1, \ldots, K$, update β_j as follows: set $\beta_j = 0$ with probability $P(\beta_j = 0|\boldsymbol{\beta}_{-j}, q, b, \hat{\boldsymbol{\beta}}) = \frac{1}{1+\text{ratio}_j}$, where $\text{ratio}_j = \frac{q}{(1-q)} \frac{\sigma_j}{b} e^{\frac{m_{j,-j}^2 \times \sigma_j^2}{2\bar{\sigma}_j^4}}$. If β_j is selected to be non-zero, simulate it from $\beta_j|\boldsymbol{\beta}_{-j}, q, b, \hat{\boldsymbol{\beta}} \sim \text{N}(\frac{\sigma_j^2 m_{j,-j}}{\bar{\sigma}_j^2}, \sigma_j^2)$, where $\frac{1}{\sigma_j^2} = \frac{1}{\bar{\sigma}_j^2} + \frac{1}{b^2}$.

5: Let $k_0 = \#\{\beta_j : \beta_j = 0,\ j = 1, \ldots, K\}$. Update q using it's full conditional posterior distribution which is a mixture of (k_0+1) Beta distributions as follows: for $j = 0, 1, \ldots, k_0,\ \ q|\boldsymbol{\beta}, b, \hat{\boldsymbol{\beta}} \sim \text{Beta}(c_1+K-j, c_2+j)$ with probability $\propto \binom{k_0}{j}(\frac{1}{\sqrt{2\pi \times b}})^{K-j} \times Beta(c_1+K-j, c_2+j)$. Here $Beta(r_1, r_2)$ denotes the normalizing constant of a $\text{Beta}(r_1, r_2)$ distribution.

6: Let $b = \frac{1}{v}$, where $v > 0$. Suppose, we want to update b in a given range (b_0, b_1), and the corresponding range of v is given by: (v_0, v_1). We consider a uniform prior on v. Let $k_1 = K - k_0$ and $t_j = K - j + 1$.

If $k_1 > 0$, let $C = \sum_{j=1}^{K} \beta_j^2$. We update v using the transformation: $v = \frac{\sqrt{y}}{\sqrt{C}}$, where y follows a mixture of $(k_0 + 1)$ distributions – the jth distribution is a truncated (between $v_0^2 C$ – $v_1^2 C$) central chi-square distribution with degree of freedom t_j, $j = 0, 1, \ldots, k_0$. The jth distribution is selected with probability $w_j \propto w_j^{(1)} w_j^{(2)} w_j^{(3)} w_j^{(4)}$; $w_j^{(1)} = \frac{1}{(2\pi)^{\frac{K-j}{2}}} \binom{k_0}{j}(1-q)^j q^{K-j}$, $w_j^{(2)} = \frac{1}{2(v_1 - v_0)} \frac{1}{C^{\frac{t_j}{2}}}$, $w_j^{(3)} = 2^{\frac{t_j}{2}} \times \Gamma(\frac{t_j}{2})$, $w_j^{(4)} = P(v_0^2 C < \chi_{t_j}^2 < v_1^2 C)$, where $\chi_{t_j}^2$ is a central chi-square distribution with d.f. t_j.

If $k_1 = 0$, the full conditional posterior distribution of v is a mixture of $(K + 1)$ distributions. For $j = 0, 1, \ldots, K$, the jth distribution of the mixture is given by: $t_j \times \frac{v^{K-j}}{v_1^{t_j} - v_0^{t_j}}$, $v_0 < v < v_1$; and it is selected with probability $w_j \propto w_j^{(1)} \times w_j^{(2)}$, where $w_j^{(1)} = \frac{1}{v_1 - v_0} \frac{1}{(2\pi)^{\frac{K-j}{2}}} \binom{K}{j}(1-q)^j q^{K-j}$ and $w_j^{(2)} = \frac{1}{t_j}(v_1^{t_j} - v_0^{t_j})$.

7: **goto** *loop* until all MCMC iterations are finished.

If the summary statistics are uncorrelated, step 4 of Algorithm 2 is modified as: for $j = 1, \ldots, K$, set $\beta_j = 0$ with probability $P(\beta_j = 0|\boldsymbol{\beta}_{-j}, q, b, \hat{\boldsymbol{\beta}}) = \frac{1}{1+\text{ratio}_j}$, where $\text{ratio}_j = (\frac{q}{1-q}) \times \frac{\sigma_j}{b} \times \exp[\frac{\hat{\beta}_j^2 \sigma_j^2}{2 s_j^4}]$, and $\frac{1}{\sigma_j^2} = \frac{1}{s_j^2} + \frac{1}{b^2}$; if β_j is selected to be non-zero, simulate it from $\beta_j|\boldsymbol{\beta}_{-j}, q, b, \hat{\boldsymbol{\beta}} \sim \text{N}(\frac{\sigma_j^2}{s_j^2}\hat{\beta}_j, \sigma_j^2)$. All the other steps of the algorithm remain the same.

3 Outline of mathematical derivation of the full conditional posterior distributions for Dirac spike

3.1 Correlated case

$$\hat{\beta}_j|\beta_j \sim \text{N}(\beta_j, s_j^2), \text{ and } \hat{\boldsymbol{\beta}}|\boldsymbol{\beta}, S \sim \text{MVN}(\boldsymbol{\beta}, S)$$

$$\beta_j|q, b \overset{i.i.d.}{\rightsquigarrow} (1-q) \times \delta_{\{0\}}(\beta_j) + q \times \text{N}(0, b^2);\ q|c_1, c_2 \sim \text{Beta}(c_1, c_2),\ 0 < q < 1.$$

$\delta_{\{0\}}(\beta_j)$ is defined as: $\delta_{\{0\}}(\beta_j) = 1$ when $\beta_j = 0$, and $\delta_{\{0\}}(\beta_j) = 0$ when $\beta_j \neq 0$. The full likelihood of the model is given by: $[\hat{\boldsymbol{\beta}}|\boldsymbol{\beta}] \times [\boldsymbol{\beta}|q, b] \times [q] \times [b]$, where $[\boldsymbol{\beta}|q, b] = \prod_{j=1}^{K}[\beta_j|q, b]$. Next we derive the full conditional posterior distributions of different parameters. Let $\hat{\boldsymbol{\beta}}_{-j} = \{\hat{\beta}_1, \ldots, \hat{\beta}_{j-1}, \hat{\beta}_{j+1}, \ldots, \hat{\beta}_K\}$ and $\boldsymbol{\beta}_{-j} = \{\beta_1, \ldots, \beta_{j-1}, \beta_{j+1}, \ldots, \beta_K\}$.

3.1.1 Full conditional posterior distribution of β

For $j = 1$, it can be shown that:

$$\begin{aligned}[\beta_1|\boldsymbol{\beta}_{-1}, q, b, \hat{\boldsymbol{\beta}}] &\propto [\hat{\boldsymbol{\beta}}, \beta_1, \boldsymbol{\beta}_{-1}, q, b] \\ &\propto [\hat{\beta}_1|\hat{\boldsymbol{\beta}}_{-1}, \beta_1, \boldsymbol{\beta}_{-1}, q, b] \times [\hat{\boldsymbol{\beta}}_{-1}, \beta_1, \boldsymbol{\beta}_{-1}, q, b] \\ &\propto [\hat{\beta}_1|\hat{\boldsymbol{\beta}}_{-1}, \boldsymbol{\beta}] \times [\beta_1|q, b]\end{aligned} \tag{14}$$

A general result: Suppose, $Y \sim \text{MVN}(\mu, \Sigma)$ and (Y_1, Y_2) is a partition of Y with the corresponding partition of the mean vector and the covariance matrix as: $\mu = (\mu_1, \mu_2)$ and

$$\Sigma = \begin{bmatrix} \Sigma_{11} & \Sigma_{12} \\ \Sigma_{21} & \Sigma_{22} \end{bmatrix}$$

Then, $Y_1|Y_2 = y_2 \sim \text{MVN}(\bar{\mu}, \bar{\Sigma})$, where $\bar{\mu} = \mu_1 + \Sigma_{12}\Sigma_{22}^{-1}(y_2 - \mu_2)$ and $\bar{\Sigma} = \Sigma_{11} - \Sigma_{12}\Sigma_{22}^{-1}\Sigma_{21}$.

Using the above general result, we can obtain $[\hat{\beta}_1|\hat{\boldsymbol{\beta}}_{-1}, \boldsymbol{\beta}]$. Let the partition of S according to the partition of $\boldsymbol{\beta} = (\beta_1, \boldsymbol{\beta}_{-1})$ be given by:

$$S = \begin{bmatrix} S_{11} & S_{1,-1} \\ S_{-1,1} & S_{-1,-1} \end{bmatrix}$$

Here, $S_{11} = s_1^2$ is a scalar, $S_{1,-1}(= (s_2^2, \ldots, s_K^2))$ is a vector of length $(K-1)$, $S_{-1,-1}$ is a matrix of order $(K-1) \times (K-1)$. Thus, $\hat{\beta}_1|\hat{\boldsymbol{\beta}}_{-1}, \boldsymbol{\beta} \sim \text{MVN}(\bar{\mu}_1, \bar{\Sigma}_1)$, where $\bar{\mu}_1 = \beta_1 + S_{1,-1} \times S_{-1,-1}^{-1} \times (\hat{\boldsymbol{\beta}}_{-1} - \beta_{-1}) = \beta_1 + \Sigma_{1,-1} \times (\hat{\boldsymbol{\beta}}_{-1} - \beta_{-1})$; $\Sigma_{1,-1} = S_{1,-1} \times S_{-1,-1}^{-1}$. And, $\bar{\Sigma}_1 = S_{11} - S_{1,-1} \times S_{-1,-1}^{-1} \times S_{-1,1} = \bar{\sigma}_1^2$, say. Thus,

$$[\hat{\beta}_1|\hat{\boldsymbol{\beta}}_{-1}, \boldsymbol{\beta}] = \frac{1}{\sqrt{2\pi}\bar{\sigma}_1} e^{-\frac{1}{2\bar{\sigma}_1^2}(\beta_1 - m_{1,-1})^2}; \text{ where } m_{1,-1} = \hat{\beta}_1 - \Sigma_{1,-1}(\hat{\boldsymbol{\beta}}_{-1} - \boldsymbol{\beta}_{-1}). \tag{15}$$

Hence,

$$\begin{aligned}[\beta_1|\boldsymbol{\beta}_{-1}, q, b, \hat{\boldsymbol{\beta}}] &= [\hat{\beta}_1|\hat{\boldsymbol{\beta}}_{-1}, \boldsymbol{\beta}] \times [\beta_1|q, b] \\ &= \frac{1}{\sqrt{2\pi}\bar{\sigma}_1} e^{-\frac{1}{2\bar{\sigma}_1^2}(\beta_1 - m_{1,-1})^2} \times [(1-q) \times \delta_{\{0\}}(\beta_1) + q \times \frac{1}{\sqrt{2\pi}b} e^{-\frac{\beta_1^2}{2b^2}}]\end{aligned} \tag{16}$$

It is straightforward to derive from the above equation that:

$$\begin{aligned}[\beta_1|\boldsymbol{\beta}_{-1}, q, b, \hat{\boldsymbol{\beta}}] &= 0, \text{ with probability } \propto (1-q) \times \frac{1}{\sqrt{2\pi}\bar{\sigma}_1} e^{-\frac{m_{1,-1}^2}{2\bar{\sigma}_1^2}} \\ &= \text{N}(\frac{\sigma_1^2 m_{1,-1}}{\bar{\sigma}_1^2}, \sigma_1^2), \text{ with probability } \propto q \times \frac{\sigma_1}{\sqrt{2\pi}\bar{\sigma}_1 b} e^{-\frac{m_{1,-1}^2}{2\bar{\sigma}_1^2}(1 - \frac{\sigma_1^2}{\bar{\sigma}_1^2})},\end{aligned} \tag{17}$$

where $\frac{1}{\sigma_1^2} = \frac{1}{\bar{\sigma}_1^2} + \frac{1}{b^2}$. Hence, $\sigma_1^2 = \frac{1}{\frac{1}{\bar{\sigma}_1^2} + \frac{1}{b^2}}$.

More explicitly,

$$[\beta_1|\boldsymbol{\beta}_{-1}, q, b, \hat{\boldsymbol{\beta}}] = \text{pr}_1 \times \delta_{\{0\}}(\beta_1) + (1 - \text{pr}_1) \times \text{N}(\frac{\sigma_1^2 m_{1,-1}}{\bar{\sigma}_1^2}, \sigma_1^2), \quad (18)$$

where $\text{pr}_1 = \frac{1}{1+\text{ratio}_1}$, and $\text{ratio}_1 = \frac{q}{(1-q)} \frac{\sigma_1}{b} e^{\frac{m_{1,-1}^2 \times \sigma_1^2}{2\bar{\sigma}_1^4}}$

3.1.2 Full conditional posterior distribution of q

Next we derive the full conditional distribution of q. Let k_0 be the number of zeros in $\boldsymbol{\beta}$, and k_1 $(= K - k_0)$ be the number of non-zero elements in $\boldsymbol{\beta}$. Let $dnorm(x, \mu, \sigma)$ denote the probability density function of a normal distribution at x with mean μ and variance σ^2. Under the Dirac spike, since β_j has a positive mass $(1 - q)$ at 0, $[\beta_j = 0|q, b] = (1 - q) + q \times dnorm(0, 0, b)$. Let $\boldsymbol{\beta}_{obs}$ denote an observed value of $\boldsymbol{\beta}$ in a MCMC iteration.

$$\begin{aligned}
[q|\boldsymbol{\beta} = \boldsymbol{\beta}_{obs}, b, \hat{\boldsymbol{\beta}}] &\propto [\hat{\boldsymbol{\beta}}, \boldsymbol{\beta} = \boldsymbol{\beta}_{obs}, q, b] \\
&\propto [\boldsymbol{\beta} = \boldsymbol{\beta}_{obs}|q, b] \times [q] \\
&= \prod_{j=1}^{K} [\beta_j = \beta_{j,obs}|q, b] \times [q] \\
&= \prod_{i:\beta_{i,obs}=0} [\beta_i = \beta_{i,obs}|q, b] \times \prod_{i:\beta_{i,obs}\neq 0} [\beta_i = \beta_{i,obs}|q, b] \times [q] \\
&= \{(1 - q) + q \times dnorm(0, 0, b)\}^{k_0} \times \prod_{i:\beta_{i,obs}\neq 0} q \times dnorm(\beta_{i,obs}, 0, b) \times [q] \\
&= \{(1 - q) + q \times \frac{1}{\sqrt{2\pi}b}\}^{k_0} \times \prod_{i:\beta_{i,obs}\neq 0} q \times \frac{1}{\sqrt{2\pi}b} e^{-\frac{\beta_{i,obs}^2}{2b^2}} \times [q] \\
&= \{(1 - q) + q \times \frac{1}{\sqrt{2\pi}b}\}^{k_0} \times q^{k_1} \times (\frac{1}{\sqrt{2\pi}b})^{k_1} e^{-\frac{\sum_{i:\beta_{i,obs}\neq 0} \beta_{i,obs}^2}{2b^2}} \times [q] \\
&= \sum_{j=0}^{k_0} \binom{k_0}{j} \times (1 - q)^j \times q^{k_0-j} \times (\frac{1}{\sqrt{2\pi}b})^{k_0-j} \times q^{k_1} \times (\frac{1}{\sqrt{2\pi}b})^{k_1} \times e^{-\frac{const}{b^2}} \times [q]; \; const = \frac{\sum_{i=1}^{K} \beta_{i,obs}^2}{2} \\
&\propto \sum_{j=0}^{k_0} \binom{k_0}{j} \times (1 - q)^j \times q^{k_0-j+k_1} \times (\frac{1}{\sqrt{2\pi}b})^{k_0-j+k_1} \times \frac{1}{Beta(c_1, c_2)} q^{c_1-1}(1 - q)^{c_2-1} \\
&\propto \sum_{j=0}^{k_0} \binom{k_0}{j} (\frac{1}{\sqrt{2\pi}b})^{K-j} \times Beta(c_1 + K - j, c_2 + j) \times [q \sim \text{Beta}(c_1 + K - j, c_2 + j)]
\end{aligned} \quad (19)$$

Thus, the full conditional posterior distribution of q is a mixture of $(k_0 + 1)$ Beta distributions as follows: for $j = 0, 1, \ldots, k_0$, $q|\boldsymbol{\beta}, b, \hat{\boldsymbol{\beta}} \sim \text{Beta}(c_1 + K - j, c_2 + j)$ with probability $\propto \binom{k_0}{j}(\frac{1}{\sqrt{2\pi}b})^{K-j} \times Beta(c_1 + K - j, c_2 + j)$. Here $Beta(r_1, r_2)$ denotes the normalizing constant of the $\text{Beta}(r_1, r_2)$ distribution.

3.1.3 Full conditional posterior distribution of b

Since $b > 0$, let $b = \frac{1}{v}$, where $v > 0$. Thus, for $j = 1, \ldots, K$, $\beta_j | q, v \overset{i.i.d.}{\sim} (1-q) \times \delta_{\{0\}}(\beta_j) + q \times \mathrm{N}(0, \frac{1}{v^2})$. Suppose, we want to update b in a given range (b_0, b_1). Let the corresponding range of v be given by (v_0, v_1). We assume a uniform prior on v. So, $[v] = \frac{1}{v_1 - v_0}$, where $v_0 < v < v_1$. Suppose, $(\beta_{1,obs}, \ldots, \beta_{K,obs})$ denote an observed value of $(\beta_1, \ldots, \beta_K)$ in a MCMC iteration. Let $k_1 = \#\{\beta_j \neq 0 : j = 1, \ldots, K\}$.

Case 1: $k_1 > 0$

$$
\begin{aligned}
[v|\boldsymbol{\beta}, q, \hat{\boldsymbol{\beta}}] &\propto [\hat{\boldsymbol{\beta}}, \boldsymbol{\beta}, q, v] \\
&\propto [\boldsymbol{\beta}|q, v] \times [v] \\
&= \prod_{j=1}^{K} [\beta_j | q, v] \times [v] \\
&= \prod_{i:\beta_{i,obs}=0} [\beta_i = \beta_{i,obs} | q, v] \prod_{i:\beta_{i,obs}\neq 0} [\beta_i = \beta_{i,obs} | q, v] \times [v] \\
&= \prod_{i:\beta_{i,obs}=0} \{(1-q) + q \times \frac{v}{\sqrt{2\pi}}\} \prod_{i:\beta_{i,obs}\neq 0} q \times \frac{v}{\sqrt{2\pi}} e^{-\frac{v^2 \beta_{i,obs}^2}{2}} \times [v] \\
&= \{(1-q) + q \times \frac{v}{\sqrt{2\pi}}\}^{k_0} \times q^{k_1} \times \frac{v^{k_1}}{(\sqrt{2\pi})^{k_1}} \times e^{-\frac{v^2}{2}C} \times [v], \text{ where } C = \sum_{i=1}^{K} \beta_{i,obs}^2 \\
&= \sum_{j=0}^{k_0} \binom{k_0}{j} \times (1-q)^j \times q^{K-j} \times \frac{1}{(\sqrt{2\pi})^{K-j}} \times v^{K-j} \times e^{-\frac{v^2}{2}C} \times [v] \\
&= \sum_{j=0}^{k_0} w_j^1 \times v^{K-j} \times e^{-\frac{v^2}{2}C} \times [v], \text{ where } w_j^1 = \binom{k_0}{j} \times (1-q)^j \times q^{K-j} \times \frac{1}{(\sqrt{2\pi})^{K-j}} \\
&\propto \sum_{j=0}^{k_0} w_j^1 \times \frac{1}{v_1 - v_0} v^{K-j} \times e^{-\frac{v^2}{2}C}, \; v_0 < v < v_1.
\end{aligned}
\tag{20}
$$

Now we consider the transformation: $y = v^2 C \Rightarrow v = \frac{\sqrt{y}}{\sqrt{C}}$, and $v_0^2 C < y < v_1^2 C$. Using the above equation, we obtain that y follows a mixture of $(k_0 + 1)$ truncated central chi-square distributions as follows: for $j = 0, \ldots, k_0$, $y \sim$ truncated central $\chi^2_{t_j}$ with probability w_j, where $v_0^2 C < y < v_1^2 C$ and $t_j = K - j + 1$. The mixture weight w_j is given by: $w_j \propto w_j^{(1)} w_j^{(2)} w_j^{(3)} w_j^{(4)}$; $w_j^{(1)} = \frac{1}{(2\pi)^{\frac{K-j}{2}}} \binom{k_0}{j} (1-q)^j q^{K-j}$, $w_j^{(2)} = \frac{1}{2(v_1 - v_0)} \frac{1}{C^{\frac{t_j}{2}}}$, $w_j^{(3)} = 2^{\frac{t_j}{2}} \times \Gamma(\frac{t_j}{2})$, $w_j^{(4)} = P(v_0^2 C < \chi^2_{t_j} < v_1^2 C)$. The updated v is obtained from updated y using the transformation: $v = \frac{\sqrt{y}}{\sqrt{C}}$.

Case 2: $k_1 = 0$

Similarly, if $k_1 = 0$, we can derive the full conditional posterior distribution of v which appears to be a mixture of $(K+1)$ distributions. For $j = 0, 1, \ldots, K$, the jth distribution of the mixture is given by: $t_j \times \frac{v^{K-j}}{v_1^{t_j} - v_0^{t_j}}$, $v_0 < v < v_1$. Here $t_j = K - j + 1$. The probability of the jth mixture component is given by:

$w_j \propto w_j^{(1)} \times w_j^{(2)}$; $w_j^{(1)} = \frac{1}{v_1 - v_0} \frac{1}{(2\pi)^{\frac{K-j}{2}}} \binom{K}{j} (1-q)^j q^{K-j}$ and $w_j^{(2)} = \frac{1}{t_j}(v_1^{t_j} - v_0^{t_j})$.

3.2 Uncorrelated case

For uncorrelated summary statistics, the full conditional posterior distribution of all the parameters except $\boldsymbol{\beta}$ remain the same. The derivation of full conditional posterior distribution of $\boldsymbol{\beta}$ for uncorrelated summary statistics is straightforward and will easily follow from the derivation for correlated summary statistics.

4 Simulation model to generate phenotype data in cohort study with binary traits

We assume that a QTL with two alleles A (minor) and a has genetic effect on a contiuous multivariate phenotype $\boldsymbol{X} = (X_1, \dots, X_K)$. Let $p = P(A)$ and G denote the genotype at the QTL; $G = AA, Aa, aa$. We first simulate the genotype data at the QTL for all individuals assuming HWE. Let g denote the count of minor allele at the QTL. For $j = 1, \dots, K$, consider an additive model: $X_j = \beta_j g + e_j$, $g = 0, 1, 2$. The random residual $e_j \sim N(0, \sigma_{ej}^2)$. Thus, $E(X_j|AA) = 2\beta_j$, $E(X_j|Aa) = \beta_j$, $E(X_j|aa) = 0$. Let σ_j^2 denote the total variance of X_j. $V(X_j) = V(E(X_j|G)) + E(V(X_j|G))$, and under the above model, $V(E(X_j|G)) = 2p(1-p)\beta_j^2$. Since, $V(X_j|G) = \sigma_{ej}^2$, irrespective of the QTL genotype G, $E(V(X_j|G)) = \sigma_{ej}^2$. Hence, $\sigma_j^2 = 2p(1-p)\beta_j^2 + \sigma_{ej}^2$. Let h_j^2 denote the trait-specific heritability of X_j due to the QTL. Hence, $h_j^2 = \frac{V(E(X_j|G))}{\sigma_j^2}$. For a given h_j^2, the residual variance is given by: $\sigma_{ej}^2 = (1 - h_j^2)\sigma_j^2$. Since, $V(E(X_j|G)) = h_j^2 \sigma_j^2$, for a given (h_j^2, σ_j^2, p), $\beta_j = \sqrt{\frac{h_j^2 \sigma_j^2}{2p(1-p)}}$. We consider that $e_1, \dots, e_K$ follow multivariate normal with mean zero and a correlation matrix $((\rho_{e,jk}))$. For $j \neq k$, ρ_{ejk} is the correlation between e_j and e_k. Thus, the set of parameters that completely specifies the simulation model for generating genotype and continuous multiple phenotype data is given by: $(p, h_j^2, \sigma_j^2, \rho_{e,jk})$, for $j, k \in \{1, \dots, K\}$. For a trait not associated with the QTL, the trait-specific heritability (h_j^2) due to the QTL is zero; for an associated trait, we simulate the trait-specific heritability at random from $(0.2\% - 0.5\%)$. We consider $\sigma_j^2 = 1$ and $\rho_{e,jk} = 0.5$ for all j, k. Finally, we dichotomize each continuous phenotype (liability) into a binary trait subject to a disease prevalence of 10% in the whole population.

5 Comparison between CPBayes and GPA

Chung et al. [2014] introduced GPA to incorporate pleiotropy and annotation information for multiple GWAS to accurately prioritize risk SNPs. We briefly outline the basic probabilistic model underlying GPA in pleiotropy context. Consider two traits Y_1, Y_2 and M SNPs. For j^{th} SNP, $j = 1, \dots, M$, let P_{j1} and P_{j2} denote the univariate p-values for Y_1 and Y_2. Let $Z_{j,00}, Z_{j,10}, Z_{j,01}$ and $Z_{j,11}$ denote the association status of jth SNP with Y_1, Y_2 as follows: $Z_{j,00} = 1$ means neither Y_1 nor Y_2 is associated with j^{th} SNP, $Z_{j,10} = 1$

means Y_1 is associated but Y_2 is not, $Z_{j,01} = 1$ means Y_2 is associated but Y_1 is not, $Z_{j,11} = 1$ means both Y_1 and Y_2 are associated. The probabilistic model of GPA is as follows:

$$
\begin{aligned}
&\Pr(Z_{j,00} = 1) = \pi_{00},\ P_{j1}|Z_{j,00} = 1 \sim \text{U}[0,1] \text{ and } P_{j2}|Z_{j,00} = 1 \sim \text{U}[0,1] \\
&\Pr(Z_{j,10} = 1) = \pi_{10},\ P_{j1}|Z_{j,10} = 1 \sim \text{Beta}(\alpha_1, 1) \text{ and } P_{j2}|Z_{j,10} = 1 \sim \text{U}[0,1] \\
&\Pr(Z_{j,01} = 1) = \pi_{01},\ P_{j1}|Z_{j,01} = 1 \sim \text{U}[0,1] \text{ and } P_{j2}|Z_{j,01} = 1 \sim \text{Beta}(\alpha_2, 1) \\
&\Pr(Z_{j,11} = 1) = \pi_{11},\ P_{j1}|Z_{j,11} = 1 \sim \text{Beta}(\alpha_1, 1) \text{ and } P_{j2}|Z_{j,11} = 1 \sim \text{Beta}(\alpha_2, 1),
\end{aligned}
$$

where $0 < \alpha_1, \alpha_2 < 1$. In the Beta distribution, first shape parameter is set smaller than the second shape parameter to induce a small p-value when the corresponding phenotype is associated. For more details, see Chung et al. [2014].

5.1 Comparison study

We compare CPBayes with GPA for two non-overlapping case-control studies since GPA does not account for shared controls. As the summary statistics are uncorrelated here, we implement the uncorrelated version of CPBayes. We consider 1000 SNPs of which $r\%$ ($r = 1, 2$) are risk SNPs (associated with at least one trait) and $(100 - r)\%$ are null SNPs (not associated with both traits). SNPs are considered to be in linkage equilibrium (a modeling assumption in GPA).

We consider three different settings: one trait is associated with all the risk SNPs (setting 1), both traits are associated with all the risk SNPs (setting 2), half of the risk SNPs are associated with one trait and the other half of the risk SNPs are associated with both traits (setting 3). Initially, in all three settings, we assumed that the minor allele at each risk SNP is positively associated with a non-null trait. Later, under setting 1, we also considered that the minor allele at each risk SNP is negatively associated with a non-null trait, and under setting 2, the minor allele at each risk SNP is positively associated with the first trait and negatively associated with the second trait. We simulate the minor allele frequency (MAF) across all the SNPs randomly from the Uniform$(0.05, 0.5)$ distribution. The odds ratio for a positively associated trait is randomly simulated from Uniform$(1.05, 1.25)$. The odds ratio for a negatively associated trait is generated from $\sim$ Uniform$(1/1.25, 1/1.05)$. We note that the MAF at a risk SNP and the odds ratio for an associated trait are simulated at random from Uniform distributions. Hence, the simulation results do not depend on which (first or second) trait is chosen to be associated with a risk SNP in setting 1 (we chose the second trait), and which (first or second) trait is positively (or negatively) associated in setting 2 (we always chose the second trait to be negatively associated).

In each simulation scenario, we implement both the methods and estimate the joint posterior probability of all the four possible different configurations of association with two traits: none of the traits (p_{00}), 1st trait but not the 2nd trait (p_{10}), 2nd trait but not the 1st trait (p_{01}), both the traits (p_{11}) are associated.

In GPA, these joint posterior probabilities evaluate pleiotropy at a particular SNP. For example, a small value of p_{00} will indicate that a risk SNP is associated with at least one trait. For a pair of traits, locFDR in CPBayes is analogous to p_{00} in GPA.

Under setting 1, we present the estimates of joint posterior probabilities for first 10 null SNPs in Figure 6 when 1% of 1000 SNPs are risk SNPs and associated only with the second trait, and 99% SNPs are null. For the null SNPs, the estimate of p_{00} should be large and the estimates of p_{10}, p_{01}, p_{11} should be small. In Figure 6, we observe that both the methods correctly estimated a high value of p_{00}. To save space, we did not include the other figures presenting results for the null SNPs in other simulation settings, where we also observed that both methods correctly reflected the null association.

In Figure 7, we present the results for risk SNPs when 1% of 1000 SNPs are risk SNPs and associated only with the second trait. So, for these risk SNPs, estimated p_{01} should be large and p_{00}, p_{10}, p_{11} should be small. Here, CPBayes and GPA produce similar estimates (Figure 7). For SNP2, SNP3, and SNP5 – SNP8, most of the total posterior probability is placed on the '01' association configuration of the traits. However, for SNP1, SNP4, SNP9, with larger MAF and smaller OR for the associated trait, both the methods placed higher mass on the '00' configuration which is expected from the perspective of power and sample size issue.

In Figure 8, 1% of 1000 (10) SNPs are risk SNPs, each of which is associated with both the traits. Hence, the estimate of p_{11} should be large. Here for all the risk SNPs, CPBayes and GPA estimated a large value of p_{11}. However, at SNP4, SNP6 and SNP9, CPBayes placed a bit of posterior mass on "01" configuration. Thus, CPBayes performed a bit conservatively at these three SNPs. A possible reason is that at SNP4 the second associated trait has a modest odds ratio 1.1; at SNP6, first associated trait has a modest odds ratio 1.09; at SNP9, second associated trait has a modest odds ratio 1.07.

In Figure 9, the results for the first 10 risk SNPs are presented when 2% of 1000 (20) SNPs are risk SNPs, each of which is associated only with the second trait. At SNP1, SNP4, SNP9 in Figure 9, both the methods conservatively produced a peak at "00" configuration which is mainly due to a modest odds ratio for the associated trait. For the other seven risk SNPs, GPA wrongly estimated a high value of p_{11} implying that both the traits are associated. Here, only the second trait is associated which is correctly reflected by a large value of p_{01} estimated by CPBayes.

For brevity, we skip presenting the rest of the results in which we observed similar pattern of the two methods' performance. In summary, CPBayes and GPA produced similar estimates of the joint posterior probabilities across many simulation scenarios. However, GPA can wrongly conclude sometimes that both traits are associated even when only one of them is associated, and hence can lead to lower specificity in selection. We also performed simulations for 1000 SNPs with all of them being null (not associated with any trait) and observed that GPA consistently estimates p_{00} to be substantially lower than CPBayes (results not presented to save space). Thus, CPBayes is more robust with respect to correctly detecting a null association; however, it can be slightly more conservative than GPA in some situations as demonstrated by

the simulations.

Under setting 1 when only one trait is non-null and negatively associated, and under setting 2 when one trait is negatively and one trait is positively associated, we noticed a similar behavior of the methods. That is, CPBayes and GPA performed similarly in many scenarios, but in some cases GPA wrongly reflected a null trait to be associated. Also, CPBayes performed a bit conservatively compared to GPA in some situations. We note that it is possible to increase the sensitivity of CPBayes by choosing a smaller interval of the slab variance. However, we emphasized more on higher specificity and robustness of CPBayes in a wide range of scenarios of pleiotropy across two or more phenotypes.

In setting 3, when one half of the risk SNPs are associated with one trait and another half of the risk SNPs are associated with both traits, CPBayes and GPA again performed similarly for most of the SNPs irrespective of the number of traits associated with the risk SNP and correctly reflected the associations. For few risk SNPs, CPBayes performed slightly more conservatively than GPA, and for few risk SNPs, GPA performed more conservatively than CPBayes.

CPBayes and GPA use a mixture of two probability distributions – one to model null effects and the other to model non-null effects. While CPBayes directly models the effect estimates by a scale mixture of two normal distributions with mean zero (one with small variance to model the null effects), GPA models the univariate association p-values by a mixture of Uniform(0,1) [Beta(1,1)] distribution (modeling null effects) and a Beta distribution with its first shape parameter smaller than the second shape parameter (modeling non-null effects). This connection in the probabilistic modeling is a reason behind the similar performance of the two approaches. The conditional FDR approach and GPA are mainly suited for analyzing a pair of traits at a time. For more than two traits, all possible pairs of traits have to be separately analyzed and then combined which is not a simultaneous analysis of multiple traits. Chung et al. [2014] suggested in the GPA R-package to analyze a pair of traits at one time.

CPBayes and ASSET are explicitly designed to analyze two or more traits simultaneously. GPA fits the model by EM algorithm and CPBayes employs MCMC that allows for estimating locFDR and Bayes factor along with the marginal or joint posterior probabilities of a specific trait or a subset of traits being associated. While CPBayes and ASSET explicitly adjust for correlation between summary statistics, GPA does not allow for such correlation.

6 Summary of the measures of overall pleiotropic association obtained while evaluating selection accuracy

While evaluating the selection accuracy of different approaches in our main simulation study (see the main text), we also computed various summary measures of $\log_{10}$(Bayes factor) (abbreviated as $\log_{10}$BF), locFDR and ASSET p-value (denoted by ASTpv) obtained across 500 replications. For example, 5%, 25%, 50%, 75%

and 95% quantiles of $\log_{10}$BF provide a brief overview of its empirical distribution under a given simulation scenario. Under the global null hypothesis of no association with any trait ($K_1 = 0$), we describe the summary measures in Table 7 for overlapping case-control studies. When at least one of the phenotypes is associated ($K_1 \geq 1$), we present the summary measures only for 10 overlapping case control studies in Table 8 and 9. We skipped providing the other tables to save space.

6.1 Main observations

The summary measures in Table 7 show that $\log_{10}$BF and locFDR are very well-controlled under the global null hypothesis. For example, 95% quantile of $\log_{10}$BF is observed to be negative (BF < 1); the minimum of observed 5% quantiles of locFDR is 0.97. Note that under the global null hypothesis, ASTpv should follow a Uniform distribution. Hence the p% quantile should be $p/100$, $0 < p < 100$. For example, in Table 7, for $K = 5$, 5% quantile of ASTpv is 0.09 and 0.07 when $m = 0.3$ and 0.1, respectively. But the 25% and 50% quantiles are 0.46 and 0.80, respectively, when $K = 5, m = 0.3$. Such inflation also increases as K increases (Table 7). So ASSET seems to control the false positive rate over-stringently. We observed similar pattern for non-overlapping case-control studies and cohort study as well.

When at least one of the traits is associated, as expected, given a choice of K, $\log_{10}$BF (locFDR) increases (decreases) as K_1 increases. For example, in Table 8, for $K_1 = 2$ ($K_1^+ = 2, K_1^- = 0$) and $m = 0.1$, the mean and median of $\log_{10}$BF (locFDR) are 10.25 (0.24) and 4.05 (4.05×10^{-05}); whereas for $K_1 = 4$ ($K_1^+ = 4, K_1^- = 0$) and $m = 0.1$, the mean and median of $\log_{10}$BF (locFDR) are 26.78 (0.09) and 16.91 (9.43×10^{-19}). For overlapping case-control studies when the summary statistics are expected to be correlated, for the same choice of K_1, when the non-null effects are both positive and negative, $\log_{10}$BF (locFDR) tends to increase (decrease) in comparison with when all the non-null effects are positive. For example, in Table 8, for $K_1^+ = 4, K_1^- = 0$ ($K_1 = 4$) and $m = 0.3$, the mean and median of $\log_{10}$BF (locFDR) are 56.53 (0.009) and 52.24 (5.96×10^{-55}); whereas for $K_1^+ = 2, K_1^- = 2$ ($K_1 = 4$) and $m = 0.3$, the mean and median of $\log_{10}$BF (locFDR) are 77.76 (0.0008) and 72.83 (2.6×10^{-75}). We also observe that ASTpv behaves similarly to locFDR.

For each simulation scenario in Table 8 and 9, we provide the percentage of replications in which the combined strategy of CPBayes chose the uncorrelated version (denoted by uncor%). From the tables, we observe that uncor% increases as K_1 increases for a given choice of K. For example, in Table 9, for $m = 0.3$, uncor% increases from 3% to 5.6% as K_1 increases from 6 ($K_1^+ = 6, K_1^- = 0$) to 8 ($K_1^+ = 8, K_1^- = 0$). For a sparse scenario when less than half of the traits are associated (e.g., $K_1 = 2$ and $K = 10$), uncor% is substantially smaller than that for a non-sparse scenario (e.g., $K_1 = 8$ and $K = 10$). For example, in Table 8, for $m = 0.1$ and $K_1 = 2$ ($K_1^+ = 1, K_1^- = 1$), uncor% is 2.4%, and it increases to 21% when $K_1 = 8$ ($K_1^+ = 4, K_1^- = 4$) and $m = 0.1$ in Table 9.

For non-overlapping case-control studies and cohort study (tables not presented here to save space), we

noticed similar pattern as observed for overlapping case-control study.

7 Simulation results for 50 traits

We carried out some simulations for 50 traits. We considered the same set-up of non-overlapping and overlapping case-control studies as considered while evaluating selection accuracy of different methods, and $K_1 = 0, 5, 10$. Since ASSET is computationally very slow for 50 traits due to an extremely large number of possible subsets of traits, we only implemented CPBayes. We also applied $\text{BH}_{0.01}$ to select the non-null traits. Different summary measures of $\log_{10}\text{BF}$ and locFDR obtained across 200 replications are described in Table 10 only for non-overlapping studies (to save space). The mean specificity and sensitivity of CPBayes and $\text{BH}_{0.01}$ obtained across only those replications in which locFDR < 0.01 are provided in Table 11 for non-overlapping and overlapping studies. While selecting the associated traits, both CPBayes and $\text{BH}_{0.01}$ produced very high level of specificity. For non-overlapping studies, $\text{BH}_{0.01}$ produced marginally higher sensitivity than CPBayes (Table 11). However, for overlapping studies, CPBayes produced marginally higher sensitivity than $\text{BH}_{0.01}$ (Table 11), in particular for lower minor allele frequency (MAF $= 0.01$).

8 Comparison between continuous spike and Dirac spike

We carried out simulation study to compare the selection accuracy of two different type of spike and slab priors. We chose the same set-up of multiple overlapping case-control studies considered while evaluating the selection accuracy of different methods. We implemented the Gibbs sampling algorithm for the Dirac spike described in Algorithm S1. Since the summary statistics are correlated, we implemented the combined strategy for the Dirac spike as well as for the continuous spike. The slab variance for the Dirac spike (b^2) is considered to vary in $0.6 - 1.0$ (the same as that for the continuous spike). We compute the mean specificity and sensitivity across only those replications among a total of 200 replications in which the locFDR for the continuous spike is < 0.01. The results are provided in Figure 10 (next page).

We observe that the Dirac spike produces less specificity than the continuous spike. The Dirac spike suffers from reduced specificity more for larger number of associated traits (K_1). The continuous spike consistently yields very good level of specificity across different scenarios. The Dirac spike offers higher sensitivity, but at the expense of lower specificity compared to the continuous spike. For example, for $K = 10$ and $m = 0.1$, $K_1^+ = 4$ and $K_1^- = 4$ ($K_1 = 8$), the Dirac spike produced a mean specificity of 47% and sensitivity of 93%, whereas the continuous spike gave a mean specificity of 100% and sensitivity of 68%. Similarly, for $K = 15$ and $m = 0.3$, $K_1^+ = 5$ and $K_1^- = 4$ ($K_1 = 9$), the Dirac spike gave 50% specificity and 100% sensitivity, whereas the continuous spike produced 100% specificity and 83% sensitivity.

9 Assessment of different SNP filtering thresholds to estimate the effect estimates' correlation structure in GERA cohort

In the GERA cohort analysis, we computed the correlation matrix of the effect estimates based on a set of independent null SNPs selected by using a threshold of the minimum of univariate association p-value across traits (denoted by minPV) and r^2 measuring the LD between a pair of SNPs. We chose the minPV threshold as 0.1 and r^2 threshold as 0.01. However, we also considered other choices of the SNP filtering thresholds and compared the resulting correlation matrices. In particular, we considered minPV $> 0.05, 0.1$ and $r^2 < 0.01, 0.05, 0.1$. For each of these six different combinations, we estimated the correlation matrix based on the summary statistics of the selected independent null SNPs. The number of independent null SNPs obtained by different combinations are provided at the top of Table 13. We computed distance between these correlation matrices based on three different notions of matrix distance. If a_{ij} and b_{ij} denote the $(i,j)^{th}$ element of two $m \times n$ matrices A and B, respectively, the element-wise mean absolute distance between A and B is $\frac{1}{mn}\sum_{i=1}^{m}\sum_{j=1}^{n}|a_{ij} - b_{ij}|$; the Euclidean distance is $\sqrt{\frac{1}{mn}\sum_{i=1}^{m}\sum_{j=1}^{n}(a_{ij} - b_{ij})^2}$; the element-wise maximum distance is $\max_{i=1,\ldots,m;j=1,\ldots,n}|a_{ij} - b_{ij}|$. We observe that the mean absolute and Euclidean distances between different pairs of the correlation matrices are very small (Table 13). However, the element-wise maximum distance is slightly larger than the mean absolute and Euclidean distances. This is expected, because for few pairs of traits, the correlation difference can be marginally larger than the average difference across all pairs of traits. These results show that the estimated correlation matrices were not very sensitive to the SNP filtering thresholds as long as the number of selected SNPs was sufficiently large, and our primary choice of the thresholds worked well. We also ran CPBayes for the GERA cohort analysis using the six different correlation matrices and observed that the results did not vary substantially.

At the bottom of Table 13, we also provide the distance between the correlation matrix obtained by the formulae in Equation 6 using the overlapping case-control counts and the six correlation matrices obtained by the GW summary statistics based approach using different SNP filtering thresholds. We see that even though the mean absolute and Euclidean distances are reasonably small, the element-wise maximum distance is substantially large indicating that the sample overlap based correlation matrix (using Equation 6) can be biased in the presence of environmental covariates.
