# Supplementary material for: An efficient Bayesian meta-analysis approach for studying cross-phenotype genetic associations
Source: PLoS Genet. 2018 Feb 12;14(2):e1007139. doi: 10.1371/journal.pgen.1007139 (PMC5825176; doi:10.1371/journal.pgen.1007139)

S1 Fig: An example diagram of the continuous spike and slab prior used by CPBayes to model pleiotropy. In this diagram, the spike variance is chosen as 0.1. However, we set this value to 10^{-4} in our simulation study and real data analysis (a diagram corresponding to this choice is presented in Figure 2).

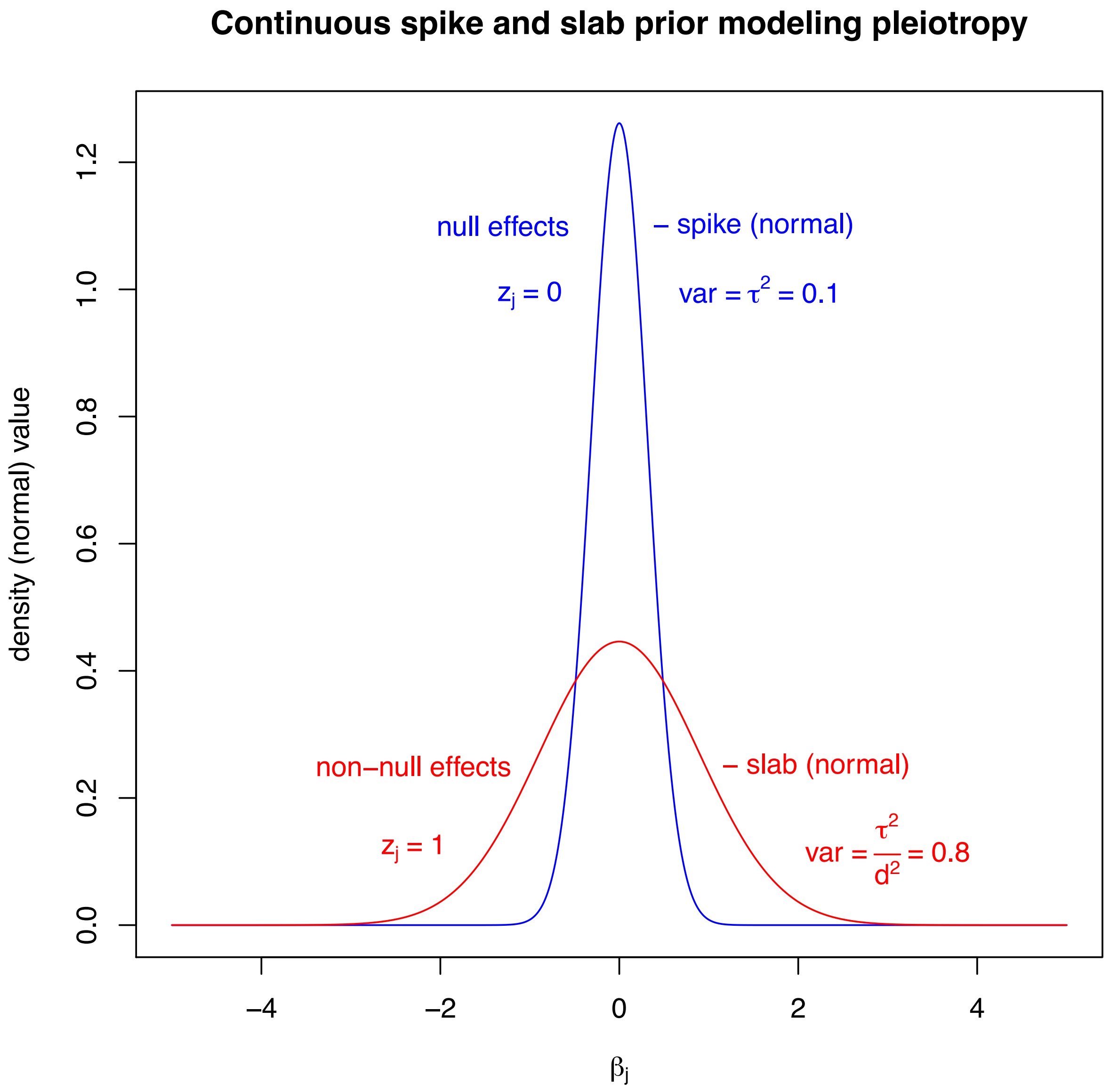

Supplement: S1 Fig — In this diagram, the spike variance is chosen as 0.1. (PDF) [file pgen.1007139.s002.pdf]
