# Supplementary material for: An efficient Bayesian meta-analysis approach for studying cross-phenotype genetic associations
Source: PLoS Genet. 2018 Feb 12;14(2):e1007139. doi: 10.1371/journal.pgen.1007139 (PMC5825176; doi:10.1371/journal.pgen.1007139)

S2 Fig: A diagram presenting the continuous spike and slab prior modeling pleiotropy with the spike variance $\tau^2 = 10^{-4}$.

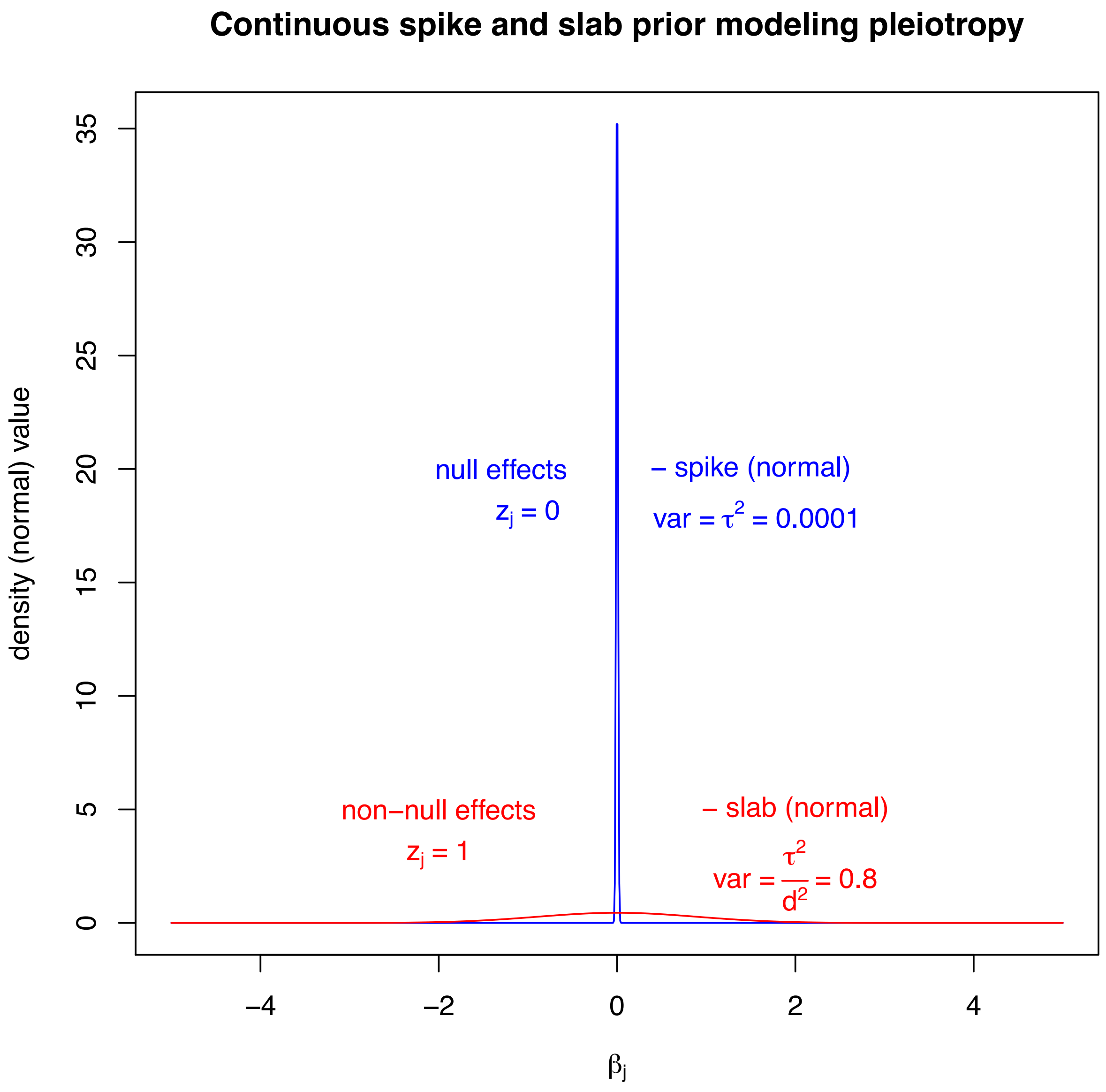

Supplement: S2 Fig — (PDF) [file pgen.1007139.s003.pdf]
