# Supplementary material for: An efficient Bayesian meta-analysis approach for studying cross-phenotype genetic associations
Source: PLoS Genet. 2018 Feb 12;14(2):e1007139. doi: 10.1371/journal.pgen.1007139 (PMC5825176; doi:10.1371/journal.pgen.1007139)

S4 Fig: Selection accuracy of different methods for 15 non-overlapping case-control studies. The total number of studies is denoted by K and m denotes the minor allele frequency at the risk SNP. K_1^+ and K_1^- denote the number of positively and negatively associated traits, respectively. Two different colors for each method present two scenarios: 1. all non-null effects are positive (++), 2. non-null effects are both positive and negative (+−).

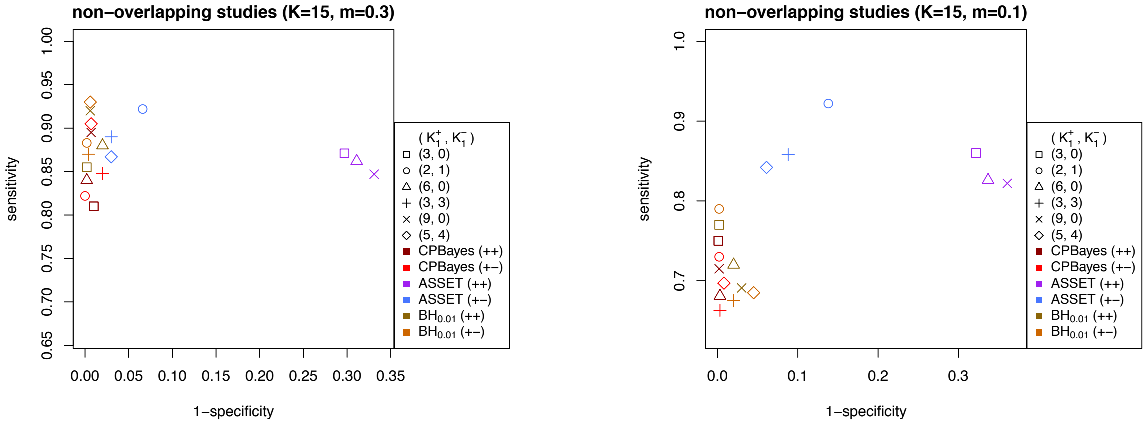

Supplement: S4 Fig — The total number of studies is denoted by K and m denotes the minor allele frequency at the risk SNP. K1+ and K1- denote the number of positively and negatively associated traits, respectively. Two different colors for each method present two scenarios: 1. all non-null effects are positive (++), 2. non-null effects are both positive and negative (+−). (PDF) [file pgen.1007139.s005.pdf]
