# Supplementary figures and images for: An efficient Bayesian meta-analysis approach for studying cross-phenotype genetic associations
Source: PLoS Genet. 2018 Feb 12;14(2):e1007139. doi: 10.1371/journal.pgen.1007139 (PMC5825176; doi:10.1371/journal.pgen.1007139)

S5 Fig: Selection accuracy of different methods for 15 overlapping case-control studies.

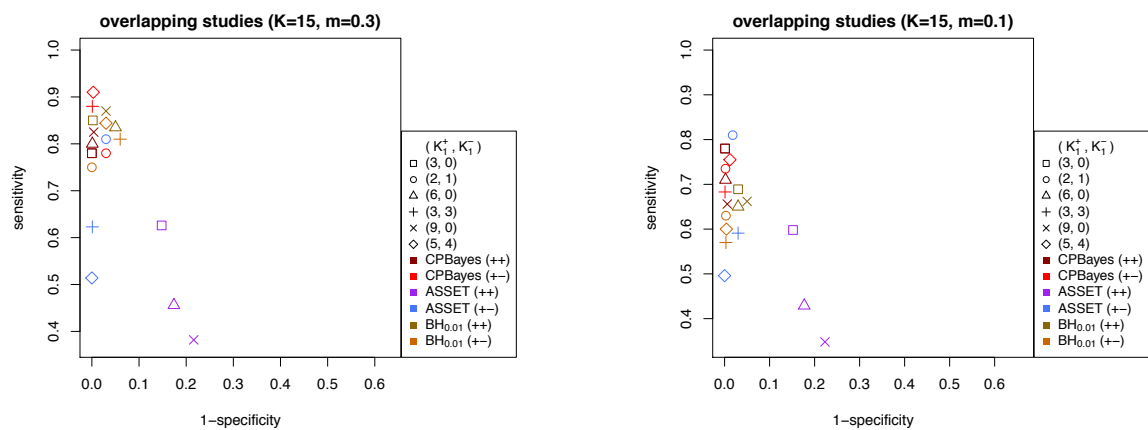

Supplement: S5 Fig — (PDF) [file pgen.1007139.s006.pdf]

S11 Fig: Forest plot for pleiotropic signal at rs6025 detected by CPBayes.

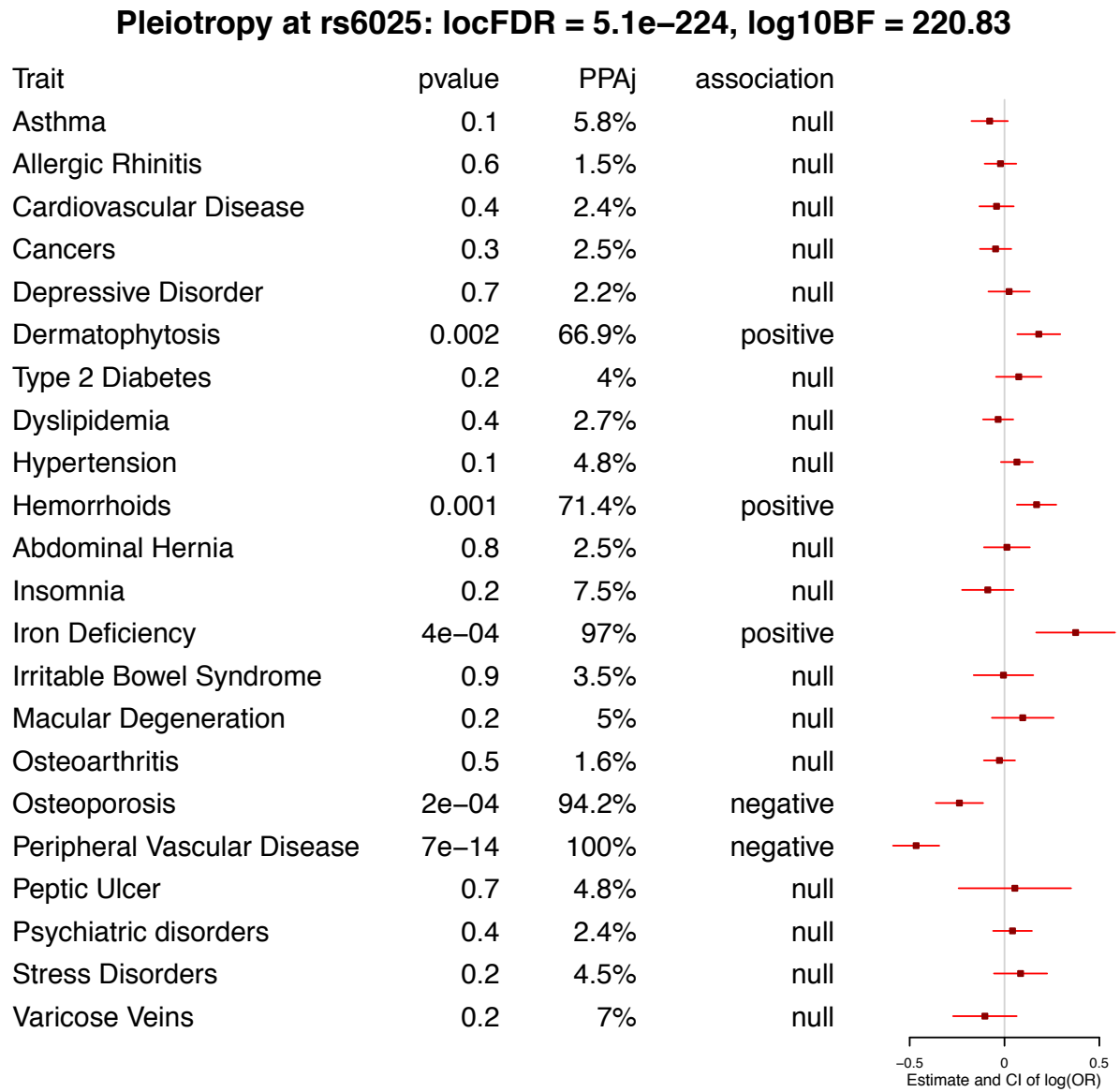

Supplement: S11 Fig — (PDF) [file pgen.1007139.s012.pdf]

S13 Fig: Forest plot for pleiotropic signal at rs10455872 detected by CPBayes.

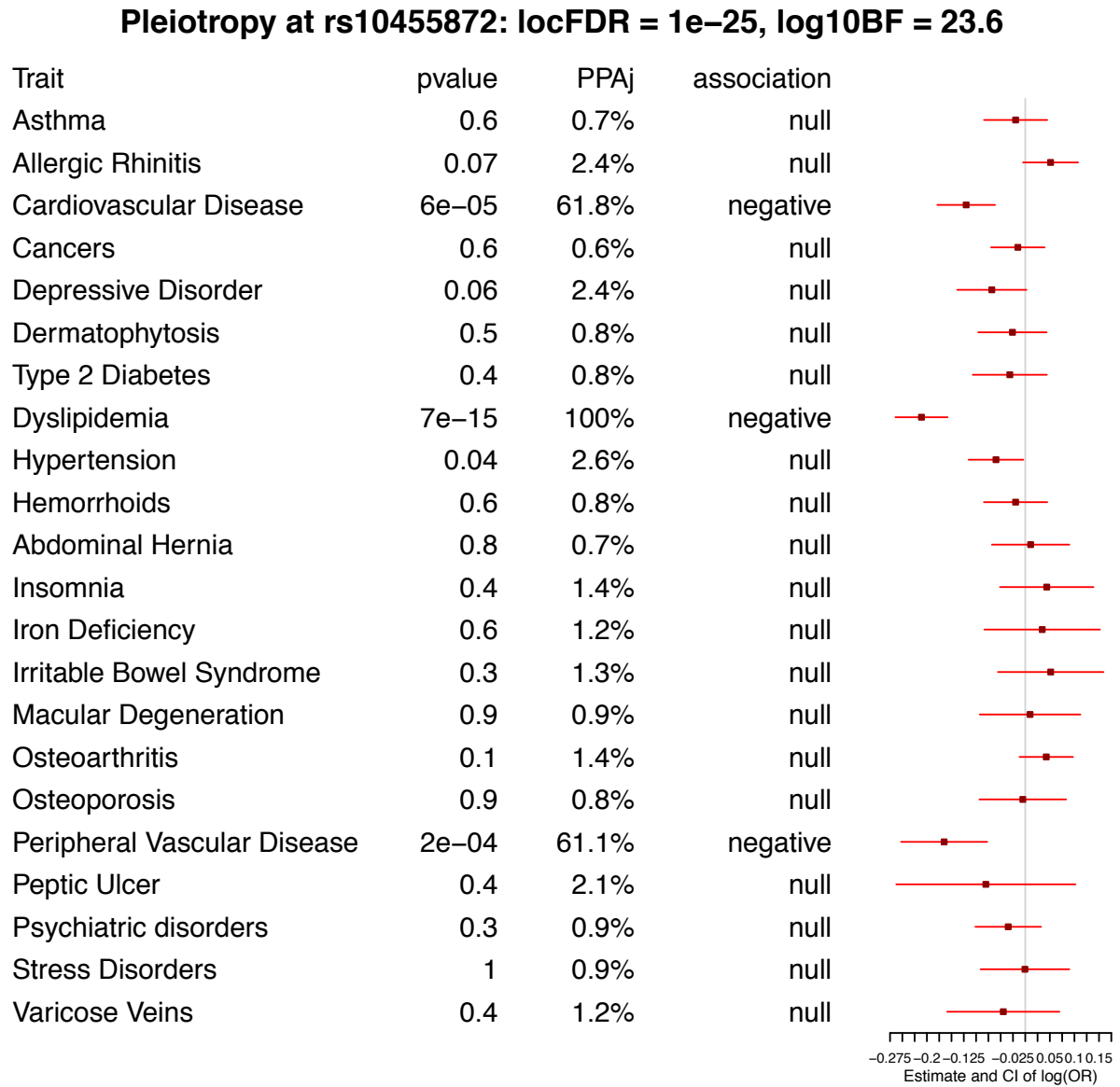

Supplement: S13 Fig — (PDF) [file pgen.1007139.s014.pdf]

S14 Fig: Forest plot for pleiotropic signal at rs3957148 detected by CPBayes.

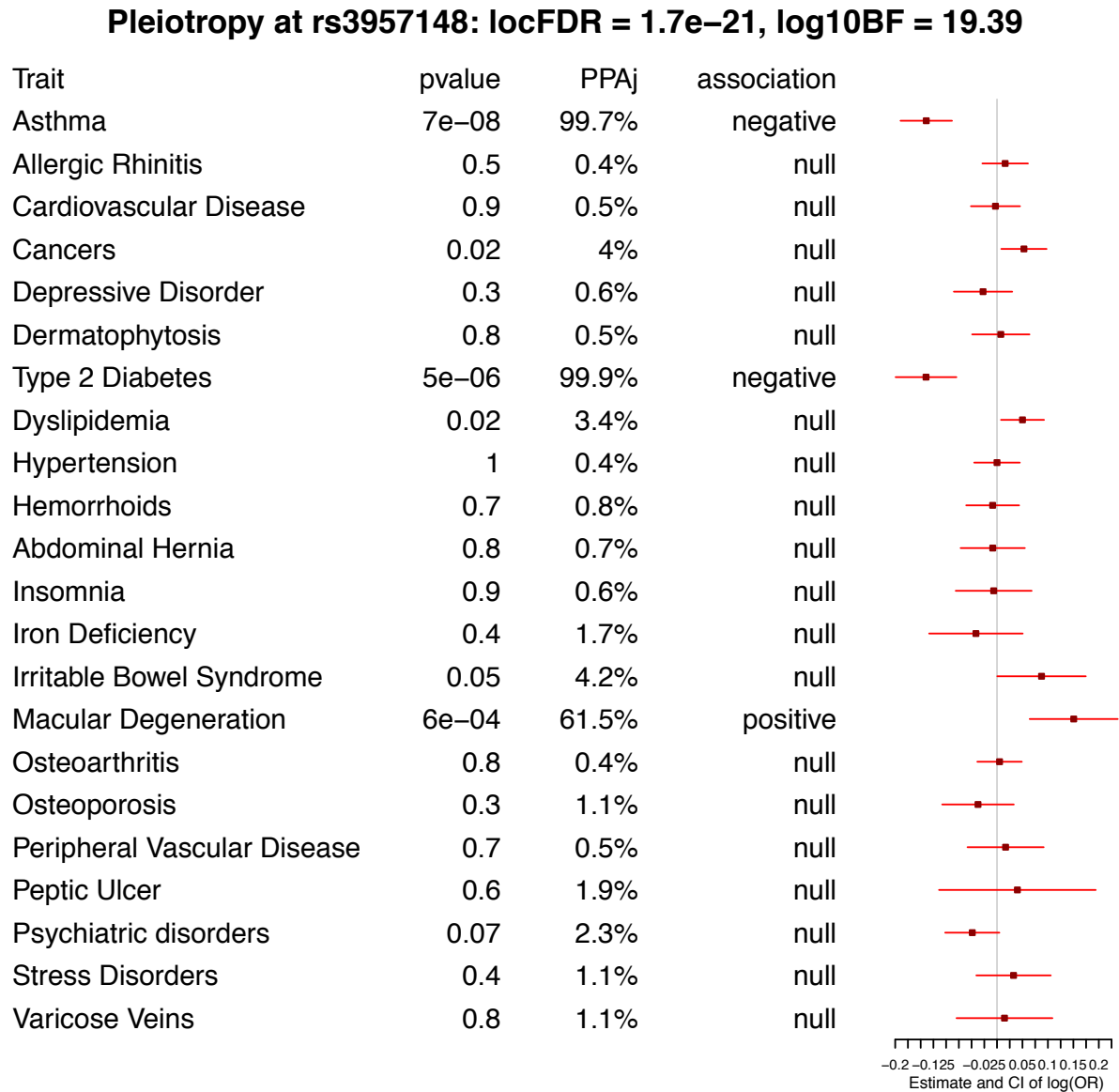

Supplement: S14 Fig — (PDF) [file pgen.1007139.s015.pdf]

S15 Fig: Forest plot for pleiotropic signal at rs687289 detected by CPBayes.

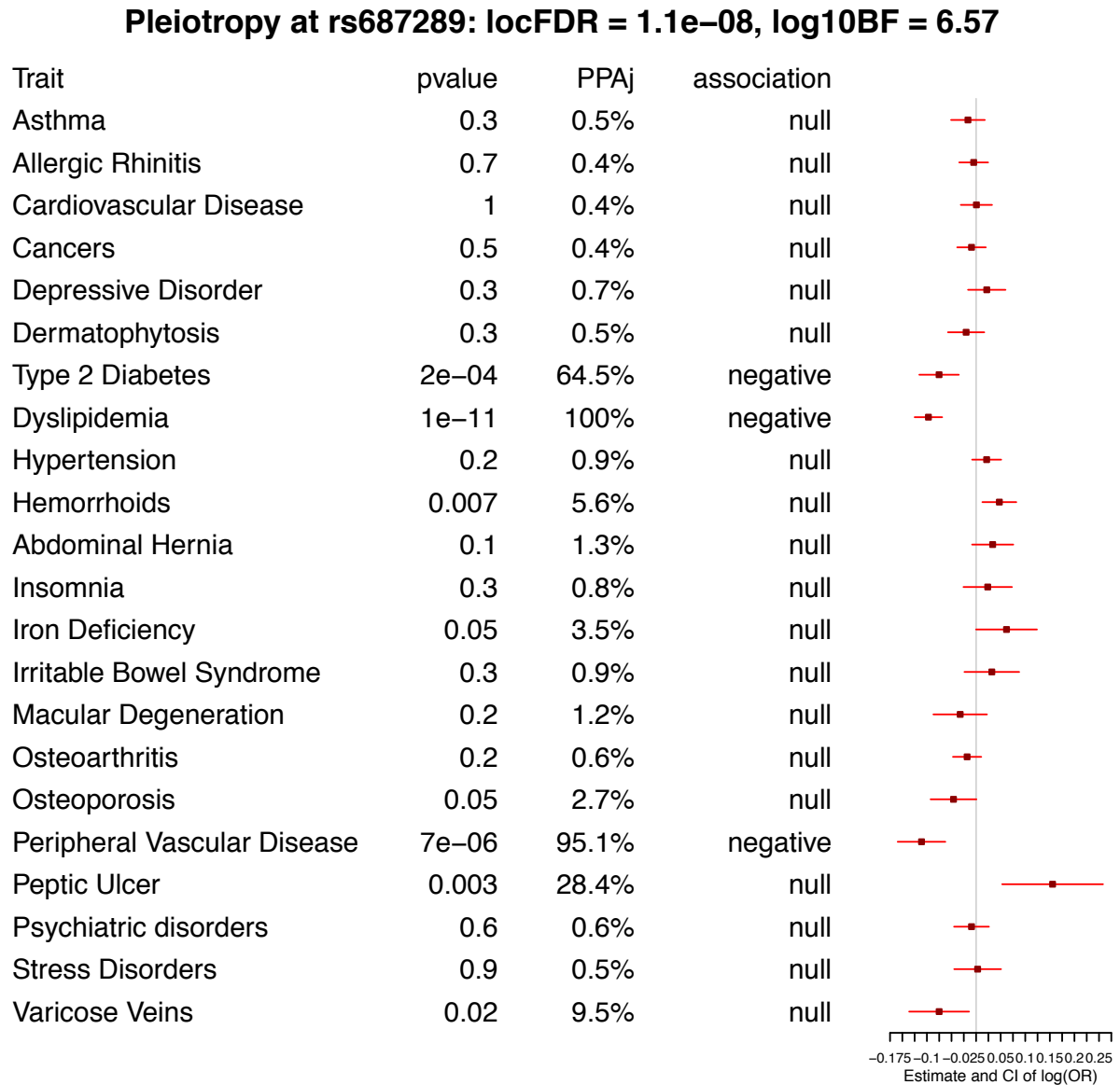

Supplement: S15 Fig — (PDF) [file pgen.1007139.s016.pdf]
