# Supplementary material for: An efficient Bayesian meta-analysis approach for studying cross-phenotype genetic associations
Source: PLoS Genet. 2018 Feb 12;14(2):e1007139. doi: 10.1371/journal.pgen.1007139 (PMC5825176; doi:10.1371/journal.pgen.1007139)

S8 Fig: Estimated joint posterior probabilities of the association configurations obtained by CPBayes and GPA for the risk SNPs. Here 1% of 1000 SNPs are risk SNPs and associated with both the traits, and 99% SNPs are null.

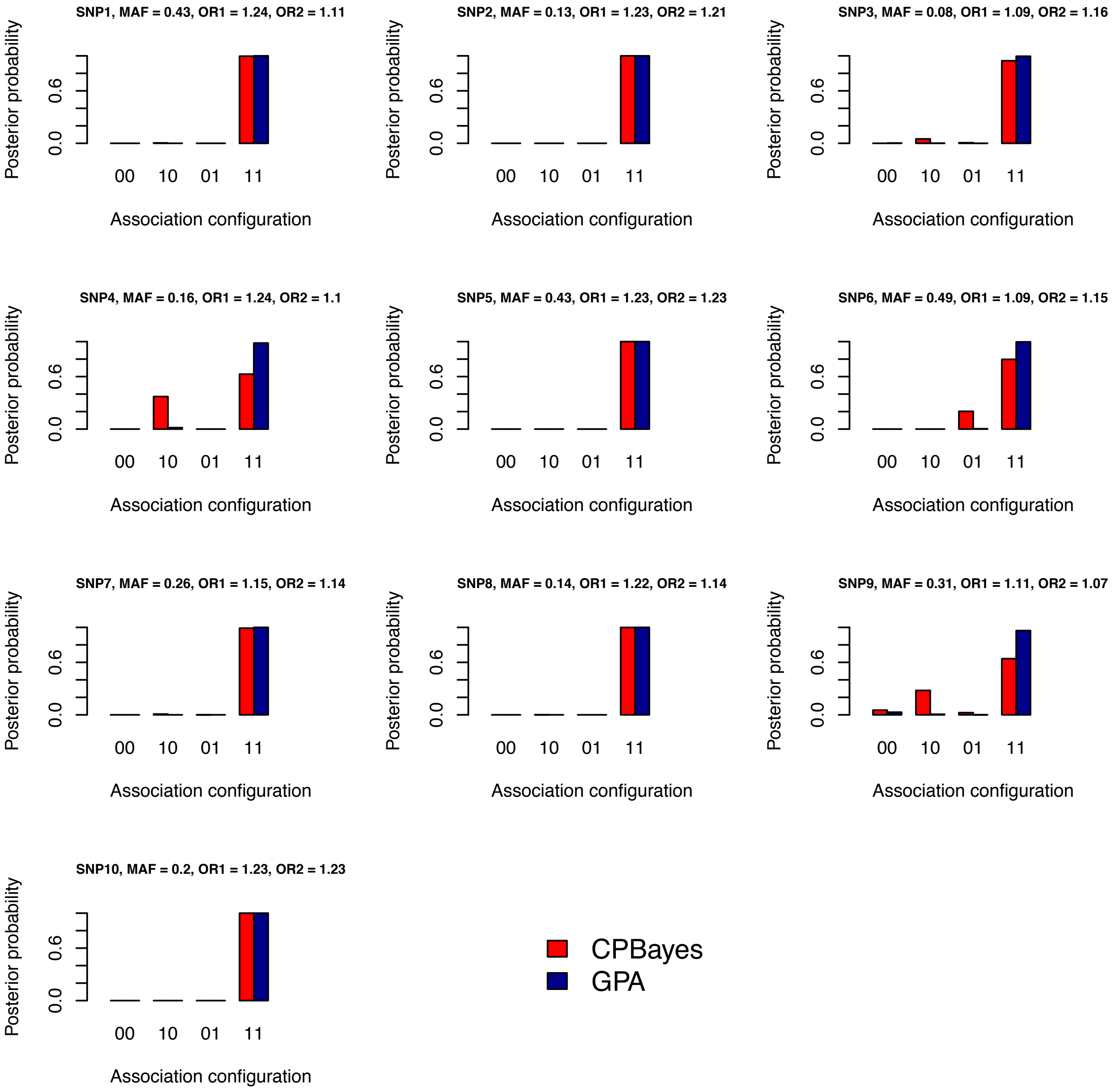

Supplement: S8 Fig — Here 1% of 1000 SNPs are risk SNPs and associated with both the traits, and 99% SNPs are null. (PDF) [file pgen.1007139.s009.pdf]
