# Supplementary material for: An efficient Bayesian meta-analysis approach for studying cross-phenotype genetic associations
Source: PLoS Genet. 2018 Feb 12;14(2):e1007139. doi: 10.1371/journal.pgen.1007139 (PMC5825176; doi:10.1371/journal.pgen.1007139)

S9 Fig: Estimated joint posterior probabilities of the association configurations obtained by CPBayes and GPA for the first 10 risk SNPs. Here 2% of 1000 SNPs are risk SNPs and associated only with the second trait, and 98% SNPs are null.

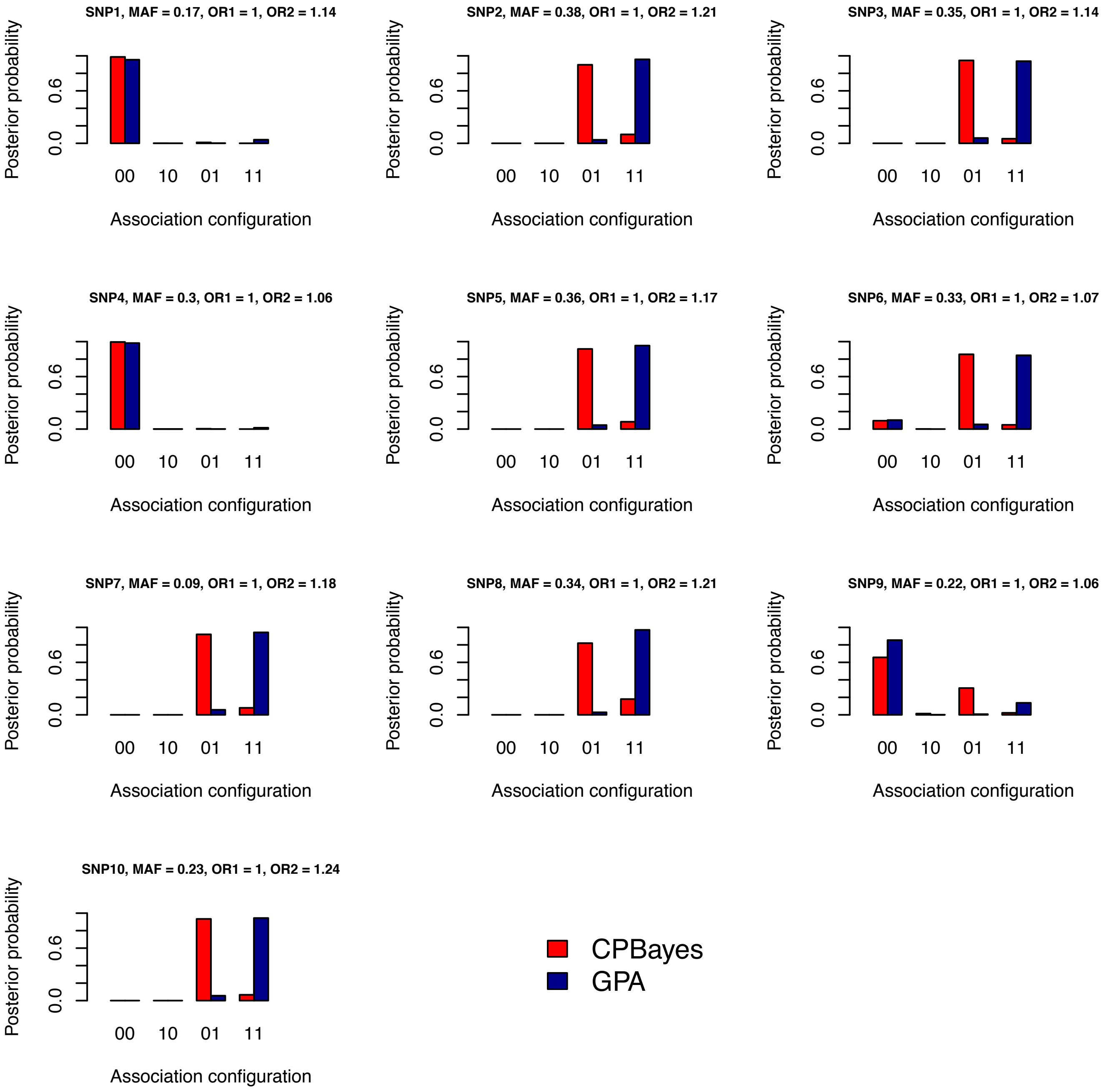

Supplement: S9 Fig — Here 2% of 1000 SNPs are risk SNPs and associated only with the second trait, and 98% SNPs are null. (PDF) [file pgen.1007139.s010.pdf]
