# Supplementary material for: An efficient Bayesian meta-analysis approach for studying cross-phenotype genetic associations
Source: PLoS Genet. 2018 Feb 12;14(2):e1007139. doi: 10.1371/journal.pgen.1007139 (PMC5825176; doi:10.1371/journal.pgen.1007139)

S10 Fig: Comparison of the accuracy of selection of associated traits by the continuous and Dirac spike for multiple overlapping case-control studies. The total number of phenotypes/studies is denoted by K and m denotes the minor allele frequency at the risk SNP.

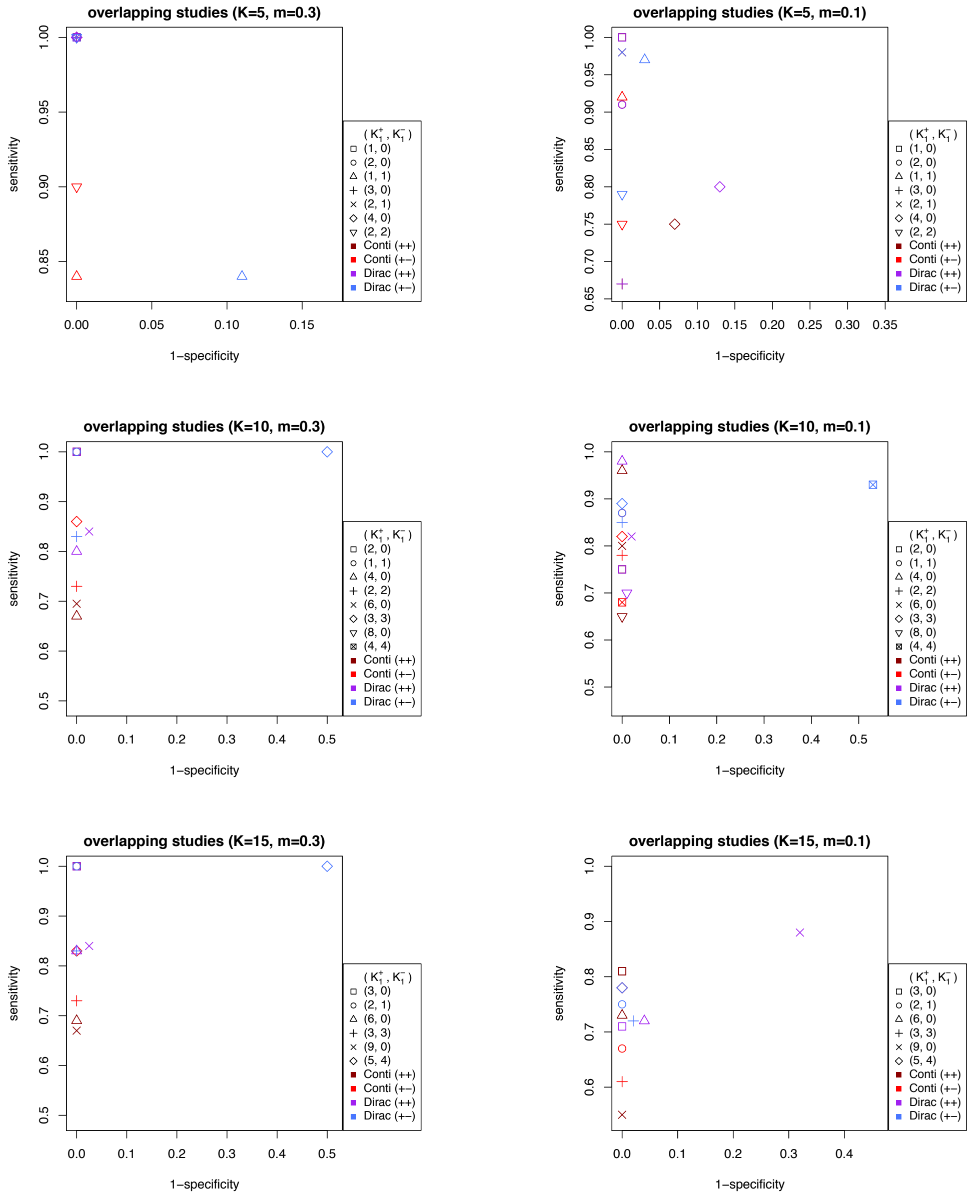

Supplement: S10 Fig — (PDF) [file pgen.1007139.s011.pdf]
