# Supplementary material for: An efficient Bayesian meta-analysis approach for studying cross-phenotype genetic associations
Source: PLoS Genet. 2018 Feb 12;14(2):e1007139. doi: 10.1371/journal.pgen.1007139 (PMC5825176; doi:10.1371/journal.pgen.1007139)

S12 Fig: Forest plot for pleiotropic signal at rs13211628 detected by CPBayes.

Pleiotropy at rs13211628: locFDR = 2e–10, log10BF = 8.32

Trait	pvalue	PPAj	association
Asthma	2e–07	98.2%	positive
Allergic Rhinitis	0.006	6.5%	null
Cardiovascular Disease	0.9	0.4%	null
Cancers	7e–05	59.2%	negative
Depressive Disorder	0.08	1.5%	null
Dermatophytosis	0.08	1.7%	null
Type 2 Diabetes	0.7	0.6%	null
Dyslipidemia	2e–10	100%	negative
Hypertension	0.9	0.4%	null
Hemorrhoids	0.06	1.8%	null
Abdominal Hernia	0.2	0.9%	null
Insomnia	0.8	0.5%	null
Iron Deficiency	0.006	14.7%	null
Irritable Bowel Syndrome	0.9	0.8%	null
Macular Degeneration	0.03	4.8%	null
Osteoarthritis	0.6	0.4%	null
Osteoporosis	0.8	0.6%	null
Peripheral Vascular Disease	0.1	1.2%	null
Peptic Ulcer	0.5	1.1%	null
Psychiatric disorders	0.7	0.5%	null
Stress Disorders	0.4	0.8%	null
Varicose Veins	0.5	1.1%	null

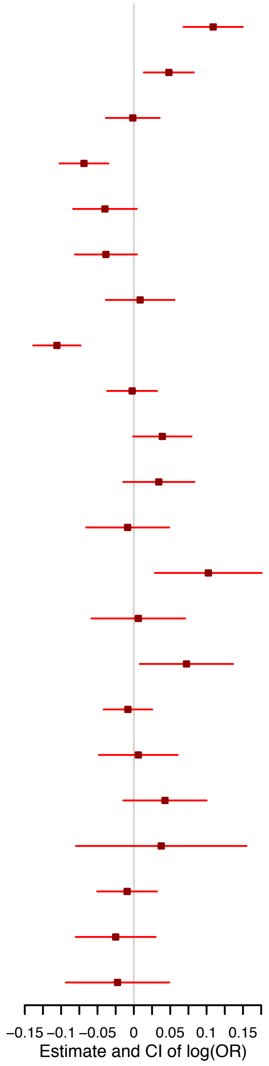

Supplement: S12 Fig — (PDF) [file pgen.1007139.s013.pdf]
