# Supplementary material for: An efficient Bayesian meta-analysis approach for studying cross-phenotype genetic associations
Source: PLoS Genet. 2018 Feb 12;14(2):e1007139. doi: 10.1371/journal.pgen.1007139 (PMC5825176; doi:10.1371/journal.pgen.1007139)
Supplement: S1 Table — (PDF) [file pgen.1007139.s017.pdf]

S1 Table: Prior probabilities of various ranges of odds ratio (OR) under different choices of the slab variance in the continuous spike and slab prior.

Slab variance	P(OR <0.98) +P(OR >1.02)	P(1.02 <OR <1.5) +P(1/1.5 <OR <1/1.02)	P(1.02 <OR <2.0) +P(1/2.0 <OR <1/1.02)
0.6	0.991	0.38	0.61
0.8	0.99	0.33	0.54
1	0.99	0.30	0.50
