# Supplementary material for: An efficient Bayesian meta-analysis approach for studying cross-phenotype genetic associations
Source: PLoS Genet. 2018 Feb 12;14(2):e1007139. doi: 10.1371/journal.pgen.1007139 (PMC5825176; doi:10.1371/journal.pgen.1007139)
Supplement: S2 Table — (PDF) [file pgen.1007139.s018.pdf]

S2 Table: Comparison between main theoretical features of CPBayes and ASSET.

Features	CPBayes	ASSET
Paradigm	Bayesian	Frequentist
Measure of overall pleiotropic association	locFDR<br>Bayes factor	P-value
Simultaneous selection of non-null traits	Yes	Yes
Specificity of selection	High	Low-high
Sensitivity of selection	Moderate-high	Low-high
Heterogeneity in direction of effects	Yes	Yes
Heterogeneity in size of effects	Yes	No
