# Supplementary material for: An efficient Bayesian meta-analysis approach for studying cross-phenotype genetic associations
Source: PLoS Genet. 2018 Feb 12;14(2):e1007139. doi: 10.1371/journal.pgen.1007139 (PMC5825176; doi:10.1371/journal.pgen.1007139)
Supplement: S3 Table — (PDF) [file pgen.1007139.s019.pdf]

S3 Table: Summary of measures of the overall pleiotropic association under the global null hypothesis of no association when multiple case-control studies with overlapping subjects are considered. We considered a distinct set of 7000 cases in each study and a common set of 10000 controls shared across all the studies.

					Quantiles				
K	m		mean	sd	5%	25%	50%	75%	95%
5	0.3	$\log_{10}$BF	-2.17	0.19	-2.36	-2.30	-2.22	-2.09	-1.80
		locFDR	0.99	0.00	0.99	0.99	0.99	1.00	1.00
		ASTpv	0.69	0.31	0.09	0.46	0.80	0.96	1.00
	0.1	$\log_{10}$BF	-1.97	0.26	-2.22	-2.15	-2.04	-1.87	-1.49
		locFDR	0.99	0.02	0.97	0.99	0.99	1.00	1.00
		ASTpv	0.69	0.31	0.07	0.47	0.79	0.96	1.00
10	0.3	$\log_{10}$BF	-2.58	0.18	-2.76	-2.70	-2.63	-2.51	-2.22
		locFDR	0.99	0.00	0.99	0.99	1.00	1.00	1.00
		ASTpv	0.85	0.29	0.11	0.90	1.00	1.00	1.00
	0.1	$\log_{10}$BF	-2.39	0.25	-2.64	-2.54	-2.45	-2.30	-1.99
		locFDR	0.99	0.03	0.98	0.99	0.99	0.99	1.00
		ASTpv	0.86	0.28	0.10	0.91	1.00	1.00	1.00
15	0.3	$\log_{10}$BF	-2.91	0.14	-3.07	-3.01	-2.95	-2.85	-2.63
		locFDR	1.00	0.00	0.99	0.99	1.00	1.00	1.00
		ASTpv	0.91	0.24	0.15	1.00	1.00	1.00	1.00
	0.1	$\log_{10}$BF	-2.73	0.20	-2.92	-2.85	-2.78	-2.67	-2.37
		locFDR	0.99	0.01	0.98	0.99	0.99	0.99	1.00
		ASTpv	0.90	0.26	0.09	0.99	1.00	1.00	1.00

K - total number of phenotypes, m - allele frequency at the marker SNP; K = 5, 10, 15, and m = 0.3, 0.1. The abbreviations used in the table are – $\log_{10}$BF: $\log_{10}$(Bayes factor), locFDR: local false discovery rate, ASTpv: ASSET p-value. For multiple studies with overlapping subjects, since the summary statistics are correlated, the combined strategy of CPBayes is implemented. Different summary measures obtained across 500 replications are provided: mean, standard deviation (sd), and 5%, 25%, 50%, 75%, 95% quantiles.
