# Supplementary material for: An efficient Bayesian meta-analysis approach for studying cross-phenotype genetic associations
Source: PLoS Genet. 2018 Feb 12;14(2):e1007139. doi: 10.1371/journal.pgen.1007139 (PMC5825176; doi:10.1371/journal.pgen.1007139)
Supplement: S4 Table — Here 2 and 4 among 10 traits are associated. (PDF) [file pgen.1007139.s020.pdf]

S4 Table: Summary of measures of the evidence of overall pleiotropic association when a subset of traits are associated for 10 overlapping case-control studies. Here 2 and 4 among 10 traits are associated.

							Quantiles				
K	K_1^+, K_1^-	m	uncor%		mean	sd	5%	25%	50%	75%	95%
10	2,0	0.3	0.2	$\log_{10}$BF	23.24	21.79	-0.96	5.40	17.69	35.80	64.70
				locFDR	0.07	0.22	2.38E-66	1.19E-37	5.10E-19	1.53E-06	0.71
				ASTpv	0.02	0.12	1.48E-17	9.04E-12	4.18E-08	7.54E-05	0.10
		0.1	1.2	$\log_{10}$BF	10.25	16.46	-2.00	-0.31	4.05	14.19	48.04
				locFDR	0.24	0.37	1.12E-49	1.73E-15	4.05E-05	0.46	0.98
				ASTpv	0.15	0.29	2.97E-08	5.79E-05	0.004	0.12	0.94
	1,1	0.3	0.8	$\log_{10}$BF	29.89	24.08	-0.30	9.18	27.33	44.94	79.91
				locFDR	0.05	0.20	1.53E-81	1.49E-46	1.02E-28	1.87E-10	0.50
				ASTpv	0.02	0.10	2.54E-22	1.08E-14	2.79E-10	7.53E-06	0.07
		0.1	2.4	$\log_{10}$BF	14.42	18.77	-1.92	0.52	7.37	23.11	52.98
				locFDR	0.18	0.34	3.63E-55	1.60E-24	2.14E-08	0.14	0.98
				ASTpv	0.12	0.26	3.42E-09	1.21E-05	0.002	0.06	0.85
10	4,0	0.3	0.6	$\log_{10}$BF	56.53	36.74	4.68	29.10	52.24	78.06	125.18
				locFDR	0.009	0.08	4.27E-128	7.90E-81	5.96E-55	1.84E-31	2.01E-06
				ASTpv	0.002	0.02	3.15E-22	1.28E-15	9.30E-12	9.47E-09	3.45E-05
		0.1	2.6	$\log_{10}$BF	26.78	30.33	-1.08	1.56	16.91	42.77	91.83
				locFDR	0.09	0.23	2.55E-94	4.78E-45	9.43E-19	0.003	0.79
				ASTpv	0.03	0.10	1.71E-10	1.33E-06	9.40E-05	0.005	0.19
	2,2	0.3	4	$\log_{10}$BF	77.76	39.68	19.01	49.97	72.83	101.41	151.72
				locFDR	0.0008	0.01	1.25E-154	3.85E-104	2.60E-75	3.98E-52	7.31E-21
				ASTpv	6.53E-05	0.001	1.01E-28	4.06E-22	6.70E-17	5.41E-13	1.94E-07
		0.1	11.6	$\log_{10}$BF	46.45	35.79	0.15	18.71	39.43	68.72	115.19
				locFDR	0.04	0.15	2.27E-117	1.51E-71	6.25E-41	3.67E-20	0.25
				ASTpv	0.008	0.05	2.58E-11	1.59E-08	1.95E-06	0.0002	0.03

K - total number of phenotypes, m - allele frequency at the risk SNP; m = 0.3, 0.1. The number of positively and negatively associated traits are denoted by K_1^+ and K_1^-, respectively. Hence the total number of associated traits is $K_1 = K_1^+ + K_1^-$. The abbreviations used in the table are – $\log_{10}$BF: $\log_{10}$(Bayes factor), locFDR: local false discovery rate, ASTpv: ASSET p-value. For multiple studies with overlapping subjects, the combined strategy of CPBayes is implemented. Of note, uncor% denotes the percentage of replications in which the combined strategy of CPBayes used the uncorrelated version of it. Different summary measures obtained across 500 replications are provided: mean, standard deviation (sd), and 5%, 25%, 50%, 75%, 95% quantiles. E-10 denotes 10^{-10}.
