# Supplementary material for: An efficient Bayesian meta-analysis approach for studying cross-phenotype genetic associations
Source: PLoS Genet. 2018 Feb 12;14(2):e1007139. doi: 10.1371/journal.pgen.1007139 (PMC5825176; doi:10.1371/journal.pgen.1007139)
Supplement: S5 Table — Here 6 and 8 among 10 traits are associated. (PDF) [file pgen.1007139.s021.pdf]

S5 Table: Summary of measures of the evidence of overall pleiotropic association when a subset of traits are associated for 10 overlapping case-control studies. Here 6 and 8 among 10 traits are associated.

							Quantiles				
K	K_1^+, K_1^-	m	uncor%		mean	sd	5%	25%	50%	75%	95%
10	6,0	0.3	3	$\log_{10}$BF	82.37	47.75	12.59	47.18	76.49	114.18	165.65
				locFDR	0.002	0.04	2.20E-169	8.35E-118	6.35E-80	1.40E-50	8.08E-16
				ASTpv	0.0002	0.01	8.67E-25	5.64E-18	1.92E-14	9.45E-11	2.64E-07
		0.1	6.8	$\log_{10}$BF	34.08	40.93	-1.12	2.56	18.16	54.59	120.22
				locFDR	0.05	0.17	1.06E-123	2.42E-57	2.28E-21	0.0003	0.42
				ASTpv	0.01	0.07	3.91E-12	8.59E-08	1.23E-05	0.0008	0.03
	3,3	0.3	6.4	$\log_{10}$BF	137.24	53.08	58.56	97.77	134.80	170.88	229.79
				locFDR	4.21E-14	9.41E-13	1.60E-233	5.82E-174	3.96E-138	1.67E-101	4.03E-62
				ASTpv	1.95E-08	3.04E-07	7.00E-33	2.24E-26	1.42E-21	3.35E-17	5.89E-12
		0.1	15	$\log_{10}$BF	95.65	56.31	4.94	55.30	90.89	134.03	198.57
				locFDR	0.01	0.08	2.62E-202	9.11E-137	8.04E-94	1.79E-57	1.14E-06
				ASTpv	0.001	0.008	1.38E-13	1.46E-10	2.02E-08	2.04E-06	0.0006
10	8,0	0.3	5.6	$\log_{10}$BF	89.30	57.79	12.67	44.88	79.87	120.42	204.79
				locFDR	0.003	0.04	1.88E-208	4.06E-124	1.50E-83	1.34E-48	2.49E-16
				ASTpv	0.0003	0.008	2.80E-27	1.84E-20	2.78E-16	7.37E-13	4.19E-09
		0.1	20.6	$\log_{10}$BF	38.82	51.46	-2.08	0.10	19.17	58.09	142.32
				locFDR	0.06	0.18	4.65E-146	9.28E-62	4.24E-22	0.004	0.52
				ASTpv	0.007	0.05	1.87E-13	3.74E-09	1.46E-06	0.0002	0.01
	4,4	0.3	5.8	$\log_{10}$BF	200.83	58.74	105.83	159.47	200.65	242.97	300.00
				locFDR	8.31E-33	1.82E-31	1.00E-300	1.04E-246	2.28E-204	3.35E-163	1.59E-109
				ASTpv	4.07E-09	9.10E-08	2.92E-36	6.13E-30	3.81E-26	5.70E-21	2.40E-15
		0.1	21	$\log_{10}$BF	152.68	74.49	29.96	98.77	153.69	205.17	272.53
				locFDR	0.003	0.04	3.12E-276	7.17E-209	2.35E-157	7.98E-102	2.60E-32
				ASTpv	0.0004	0.006	2.90E-15	2.05E-12	3.48E-10	4.65E-08	2.85E-05

K - total number of phenotypes, m - allele frequency at the risk SNP; m = 0.3, 0.1. The number of positively and negatively associated traits are denoted by K_1^+ and K_1^-, respectively. Hence the total number of associated traits is $K_1 = K_1^+ + K_1^-$. The abbreviations used in the table are – $\log_{10}$BF: $\log_{10}$(Bayes factor), locFDR: local false discovery rate, ASTpv: ASSET p-value. For multiple studies with overlapping subjects, the combined strategy of CPBayes is implemented. Of note, uncor% denotes the percentage of replications in which the combined strategy of CPBayes used the uncorrelated version of it. Different summary measures obtained across 500 replications are provided: mean, standard deviation (sd), and 5%, 25%, 50%, 75%, 95% quantiles. E-10 denotes 10^{-10}.
