# Supplementary material for: An efficient Bayesian meta-analysis approach for studying cross-phenotype genetic associations
Source: PLoS Genet. 2018 Feb 12;14(2):e1007139. doi: 10.1371/journal.pgen.1007139 (PMC5825176; doi:10.1371/journal.pgen.1007139)
Supplement: S6 Table — Summary of measures for the evidence of the overall pleiotropic association for 50 non-overlapping case-control studies. Here 0, 5, and 10 among 50 traits are associated. (PDF) [file pgen.1007139.s022.pdf]

S6 Table: Simulation study for 50 traits. Summary of measures for the evidence of the overall pleiotropic association for 50 non-overlapping case-control studies. Here 0, 5, and 10 among 50 traits are associated.

					Quantiles				
K_1^+, K_1^-	m		mean	sd	5%	25%	50%	75%	95%
0,0	0.3	log10BF	-4.41	0.21	-4.59	-4.52	-4.45	-4.36	-4.13
		locFDR	0.99	0.02	0.99	0.99	0.99	0.99	1.00
	0.1	log10BF	-4.19	0.25	-4.41	-4.35	-4.25	-4.11	-3.72
		locFDR	0.98	0.03	0.96	0.99	0.99	0.99	0.99
5,0	0.3	log10BF	113.78	60.19	19.75	69.81	108.31	152.90	226.50
		locFDR	1.8E-04	1.9E-03	3.25E-229	8.67E-156	2.82E-111	1.70E-72	8.66E-20
	0.1	log10BF	61.63	59.80	-2.27	11.00	47.14	88.93	176.83
		locFDR	0.06	0.18	9.55E-180	1.84E-91	3.76E-50	5.23E-14	0.49
3,2	0.3	log10BF	116.43	61.88	24.42	71.01	114.63	149.67	228.81
		locFDR	2.02E-09	2.81E-08	1.03E-231	1.68E-152	1.53E-117	9.50E-74	1.27E-26
	0.1	log10BF	58.25	54.98	-2.11	10.56	47.15	89.40	160.99
		locFDR	0.05	0.17	1.04E-163	2.04E-92	3.67E-50	1.43E-13	0.40
10,0	0.3	log10BF	257.96	54.55	141.78	228.03	286.80	300.00	300.00
		locFDR	1.75E-85	2.48E-84	1.38e-313	1.00E-300	2.27E-292	1.34E-231	7.88E-143
	0.1	log10BF	184.55	86.68	42.99	116.40	180.02	271.08	300.00
		locFDR	0.001	0.02	3.34E-305	1.13E-274	1.99E-183	1.20E-117	4.75E-46
5,5	0.3	log10BF	257.61	54.29	144.86	223.89	290.12	300.00	300.00
		locFDR	2.00E-114	2.71E-113	2.98e-315	1.00E-300	8.06E-295	2.11E-228	8.23E-150
	0.1	log10BF	172.58	85.36	43.18	104.64	167.87	240.96	300.00
		locFDR	0.003	0.05	1.00E-300	1.02E-244	1.28E-171	6.87E-106	1.41E-43

K - total number of phenotypes, m - allele frequency at the risk SNP; m = 0.3, 0.1. The number of positively and negatively associated traits are denoted by K_1^+ and K_1^-, respectively. Hence the total number of associated traits is $K_1 = K_1^+ + K_1^-$. The abbreviations used in the table are – $\log_{10}$BF: $\log_{10}$(Bayes factor), locFDR: local false discovery rate. For multiple studies with no overlapping subjects, the uncorrelated version of CPBayes is implemented. Different summary measures obtained across 200 replications are provided: mean, standard deviation (sd), and 5%, 25%, 50%, 75%, 95% quantiles. E-10 denotes 10^{-10}.
