# Supplementary material for: An efficient Bayesian meta-analysis approach for studying cross-phenotype genetic associations
Source: PLoS Genet. 2018 Feb 12;14(2):e1007139. doi: 10.1371/journal.pgen.1007139 (PMC5825176; doi:10.1371/journal.pgen.1007139)
Supplement: S7 Table — Accuracy in selection of associated traits by CPBayes and BH0.01 for 50 case-control studies. (PDF) [file pgen.1007139.s023.pdf]

S7 Table: Simulation study for 50 traits. Accuracy in selection of associated traits by CPBayes and $BH_{0.01}$ for 50 case-control studies. The number of positively and negatively associated traits are denoted by K_1^+ and K_1^-, respectively. And m denotes the minor allele frequency at the risk SNP.

50 non-overlapping case-control studies					
		CPBayes		$BH_{0.01}$	
K_1^+, K_1^-	m	specificity	sensitivity	specificity	sensitivity
5,0	0.3	1.00	0.80	1.00	0.84
	0.1	1.00	0.61	1.00	0.66
3,2	0.3	1.00	0.81	1.00	0.85
	0.1	1.00	0.61	1.00	0.65
10,0	0.3	1.00	0.82	1.00	0.86
	0.1	1.00	0.62	1.00	0.65
5,5	0.3	1.00	0.82	1.00	0.86
	0.1	1.00	0.58	1.00	0.62
50 overlapping case-control studies					
		CPBayes		$BH_{0.01}$	
K_1^+, K_1^-	m	specificity	sensitivity	specificity	sensitivity
5,0	0.3	1.00	0.81	1.00	0.80
	0.1	1.00	0.66	1.00	0.61
3,2	0.3	1.00	0.81	1.00	0.80
	0.1	1.00	0.62	1.00	0.55
10,0	0.3	1.00	0.83	1.00	0.83
	0.1	1.00	0.65	1.00	0.61
5,5	0.3	1.00	0.82	1.00	0.81
	0.1	1.00	0.61	1.00	0.54
