# Supplementary material for: An efficient Bayesian meta-analysis approach for studying cross-phenotype genetic associations
Source: PLoS Genet. 2018 Feb 12;14(2):e1007139. doi: 10.1371/journal.pgen.1007139 (PMC5825176; doi:10.1371/journal.pgen.1007139)
Supplement: S8 Table — (PDF) [file pgen.1007139.s024.pdf]

S8 Table: Name of 22 phenotypes in the GERA cohort analyzed by CPBayes and ASSET

Disease name	Case sample size	Control sample size
Asthma	9061	44748
Allergic Rhinitis	13584	40225
Cardiovascular Disease	14701	39108
Cancers	16719	37090
Depressive Disorder	7130	46679
Dermatophytosis	7527	46282
Type 2 Diabetes	6814	46995
Dyslipidemia	29608	24201
Hypertension	27754	26055
Hemorrhoids	8963	44846
Abdominal Hernia	6088	47721
Insomnia	3919	49890
Iron Deficiency	2441	51368
Irritable Bowel Syndrome	3051	50758
Macular Degeneration	3605	50204
Osteoarthritis	19924	33885
Osteoporosis	5335	48474
Peripheral Vascular Disease	4257	49552
Peptic Ulcer	898	52911
Psychiatric disorders	8371	45438
Stress Disorders	4244	49565
Varicose Veins	2437	51372
