# Supplementary material for: An efficient Bayesian meta-analysis approach for studying cross-phenotype genetic associations
Source: PLoS Genet. 2018 Feb 12;14(2):e1007139. doi: 10.1371/journal.pgen.1007139 (PMC5825176; doi:10.1371/journal.pgen.1007139)
Supplement: S9 Table — At the beginning of the table, the number of independent null SNPs obtained by using different SNP filtering thresholds are listed. At the bottom of the table, we also provide the distance between the correlation matrix estimated by Eq 6 based on the number of overlapping cases and controls and the GW summary statistics based approach. (PDF) [file pgen.1007139.s025.pdf]

S9 Table: Distance between estimated correlation matrices of effect estimates in the GERA cohort obtained by using different thresholds of the minimum of univariate association p-value across traits and r^2 value between a pair of SNPs to select independent null SNPs. At the beginning of the table, the number of independent null SNPs obtained by using different SNP filtering thresholds are listed. At the bottom of the table, we also provide the distance between the correlation matrix estimated by Equation 6 based on the number of overlapping cases and controls and the GW summary statistics based approach.

SNP filtering thresholds	minPV > 0.1 $r^2 < 0.01$	minPV > 0.1 $r^2 < 0.05$	minPV > 0.1 $r^2 < 0.1$	minPV > 0.05 $r^2 < 0.01$	minPV > 0.05 $r^2 < 0.05$	minPV > 0.05 $r^2 < 0.1$
Number of selected SNPs	24510	34190	38339	66164	103324	120307
	Mean absolute distance					
SNP filtering thresholds	minPV > 0.1 $r^2 < 0.01$	minPV > 0.1 $r^2 < 0.05$	minPV > 0.1 $r^2 < 0.1$	minPV > 0.05 $r^2 < 0.01$	minPV > 0.05 $r^2 < 0.05$	minPV > 0.05 $r^2 < 0.1$
minPV > 0.1 $r^2 < 0.01$	0	0.003	0.003	0.008	0.008	0.008
minPV > 0.1 $r^2 < 0.05$	0.003	0	0.001	0.008	0.008	0.008
minPV > 0.1 $r^2 < 0.1$	0.003	0.001	0	0.008	0.008	0.008
minPV > 0.05 $r^2 < 0.01$	0.008	0.008	0.008	0	0.002	0.002
minPV > 0.05 $r^2 < 0.05$	0.008	0.008	0.008	0.002	0	0.001
minPV > 0.05 $r^2 < 0.1$	0.008	0.008	0.008	0.002	0.001	0
	Euclidean distance					
	minPV > 0.1 $r^2 < 0.01$	minPV > 0.1 $r^2 < 0.05$	minPV > 0.1 $r^2 < 0.1$	minPV > 0.05 $r^2 < 0.01$	minPV > 0.05 $r^2 < 0.05$	minPV > 0.05 $r^2 < 0.1$
minPV > 0.1 $r^2 < 0.01$	0	0.003	0.004	0.011	0.011	0.011
minPV > 0.1 $r^2 < 0.05$	0.003	0	0.002	0.01	0.01	0.01
minPV > 0.1 $r^2 < 0.1$	0.004	0.002	0	0.01	0.01	0.01
minPV > 0.05 $r^2 < 0.01$	0.01	0.01	0.01	0	0.002	0.002
minPV > 0.05 $r^2 < 0.05$	0.01	0.01	0.01	0.002	0	0.001
minPV > 0.05 $r^2 < 0.1$	0.01	0.01	0.01	0.002	0.001	0
	Element-wise maximum distance					
	minPV > 0.1 $r^2 < 0.01$	minPV > 0.1 $r^2 < 0.05$	minPV > 0.1 $r^2 < 0.1$	minPV > 0.05 $r^2 < 0.01$	minPV > 0.05 $r^2 < 0.05$	minPV > 0.05 $r^2 < 0.1$
minPV > 0.1 $r^2 < 0.01$	0	0.01	0.01	0.04	0.05	0.05
minPV > 0.1 $r^2 < 0.05$	0.01	0	0.005	0.04	0.05	0.05
minPV > 0.1 $r^2 < 0.1$	0.01	0.005	0	0.04	0.05	0.05
minPV > 0.05 $r^2 < 0.01$	0.04	0.04	0.04	0	0.01	0.01
minPV > 0.05 $r^2 < 0.05$	0.05	0.05	0.05	0.01	0	0.003
minPV > 0.05 $r^2 < 0.1$	0.05	0.05	0.05	0.01	0.003	0
	Distance between correlation matrix estimated by Eq. 6 and GW strategy					
Matrix distance	minPV > 0.1 $r^2 < 0.01$	minPV > 0.1 $r^2 < 0.05$	minPV > 0.1 $r^2 < 0.1$	minPV > 0.05 $r^2 < 0.01$	minPV > 0.05 $r^2 < 0.05$	minPV > 0.05 $r^2 < 0.1$
Mean absolute	0.04	0.04	0.04	0.03	0.03	0.03
Euclidean	0.06	0.06	0.06	0.05	0.05	0.05
Element-wise max	0.24	0.24	0.23	0.22	0.21	0.21

Following abbreviations were used: minPV denotes the minimum of univariate association p-value across traits for a SNP and r^2 denotes the square of the correlation between genotypes at a pair of SNPs. While describing the distances between the correlation matrix estimated by Equation 6 and using the GW summary statistics based approach, following abbreviations were used: Mean absolute – Mean absolute distance, Euclidean – Euclidean distance, Element-wise max – element-wise maximum distance.
