# Supplementary material for: An efficient Bayesian meta-analysis approach for studying cross-phenotype genetic associations
Source: PLoS Genet. 2018 Feb 12;14(2):e1007139. doi: 10.1371/journal.pgen.1007139 (PMC5825176; doi:10.1371/journal.pgen.1007139)
Supplement: S10 Table — (PDF) [file pgen.1007139.s026.pdf]

S10 Table: Independent pleiotropic SNPs identified by CPBayes for which one phenotype was selected.

rsID	chrom band	CPBayes locFDR	CPBayes $\log_{10}$BF	Associated trait selected by CPBayes	PPA_j	Direction	Univariate p-values	Subset of associated traits detcted by $BH_{0.01}$
rs35505017	1q31.3	2.78E-173	171.59	Macular Degeneration	100%	negative	3.14E-14	Macular Degeneration
rs77394225	1q31.3	1.05E-83	82.01	Macular Degeneration	100%	negative	6.04E-16	Macular Degeneration
rs111465441	1q31.3	1.93E-11	9.75	Macular Degeneration	100%	negative	1.76E-07	Macular Degeneration
rs1367117	2p24.1	2.00E-47	45.74	Dyslipidemia	100%	negative	5.26E-43	Dyslipidemia
rs562338	2p24.1	1.14E-44	42.98	Dyslipidemia	100%	positive	3.43E-35	Dyslipidemia
rs7604788	2p24.1	2.14E-38	36.7	Dyslipidemia	100%	positive	1.19E-13	Dyslipidemia
rs12477935	2p16.1	1.87E-09	7.77	Varicose Veins	100%	negative	3.88E-06	Varicose Veins
rs11899888	2p16.1	3.83E-08	6.46	Abdominal Hernia	100%	negative	9.76E-10	Abdominal Hernia
rs780094	2p23.3	7.13E-07	5.19	Dyslipidemia	100%	negative	4.24E-13	Dyslipidemia
rs11709077	3p25.2	1.01E-10	9.03	Type 2 Diabetes	100%	positive	1.26E-09	Type 2 Diabetes
rs35407	5p13.2	2.67E-18	16.61	Cancers	100%	positive	1.07E-08	Cancers
rs12916	5q13.3	8.82E-13	11.09	Dyslipidemia	100%	negative	1.46E-22	Dyslipidemia
rs12203592	6p25.3	3.42E-84	82.50	Cancers	100%	negative	2.36E-48	Cancers
rs4151671	6p21.33	6.68E-30	28.21	Macular Degeneration	100%	positive	3.64E-08	Macular Degeneration
rs6907034	6p21.32	1.85E-19	17.77	Type 2 Diabetes	100%	negative	2.33E-07	Type 2 Diabetes
rs11575852	6p21.33	2.44E-12	10.65	Asthma	100%	negative	2.13E-06	Asthma
rs3130347	6p21.32	4.64E-10	8.37	Macular Degeneration	100%	negative	5.76E-09	Macular Degeneration
rs11962994	6p21.33	1.28E-07	5.93	Macular Degeneration	100%	positive	1.05E-06	Macular Degeneration<br>Cancers
rs16877989	6q14.3	2.48E-07	5.64	Irritable Bowel Syndrome	100%	positive	1.89E-06	Irritable Bowel Syndrome
rs2300051	7q31.1	6.72E-30	28.21	Peptic Ulcer	100%	negative	3.93E-07	Peptic Ulcer
rs74580577	9q22.33	1.14E-09	7.98	Peptic Ulcer	100%	negative	1.81E-05	Peptic Ulcer
rs11200630	10q26.13	1.00E-300	300.00	Macular Degeneration	100%	negative	5.15E-74	Macular Degeneration
rs2292627	10q26.13	6.07E-88	86.25	Macular Degeneration	100%	negative	8.23E-21	Macular Degeneration
rs2253755	10q26.13	1.80E-54	52.78	Macular Degeneration	100%	negative	2.94E-21	Macular Degeneration
rs2672589	10q26.13	1.29E-32	30.93	Macular Degeneration	100%	positive	3.36E-16	Macular Degeneration
rs7079711	10q25.2	1.93E-19	17.75	Type 2 Diabetes	100%	positive	6.74E-12	Type 2 Diabetes
rs11196187	10q25.2	1.33E-07	5.92	Type 2 Diabetes	100%	negative	2.87E-09	Type 2 Diabetes
rs7896811	10q25.2	8.29E-07	5.12	Type 2 Diabetes	100%	positive	7.25E-09	Type 2 Diabetes
rs1799963	11p11.2	2.38E-71	69.66	Peripheral Vascular Disease	100%	negative	1.19E-08	Peripheral Vascular Disease
rs964184	11q23.3	1.46E-33	31.87	Dyslipidemia	100%	negative	5.49E-28	Dyslipidemia
rs3736508	11p11.2	3.74E-15	13.47	Iron Deficiency	100%	negative	3.60E-06	Iron Deficiency
rs60538784	11q13.2	5.76E-15	13.28	Osteoporosis	100%	negative	7.85E-10	Osteoporosis
rs74836424	16q24.3	7.71E-20	18.15	Cancers	100%	negative	1.14E-12	Cancers
rs2000999	16q22.2	1.16E-07	5.98	Dyslipidemia	100%	negative	2.98E-15	Dyslipidemia
rs1801689	17q24.2	1.86E-13	11.77	Dyslipidemia	100%	negative	4.80E-09	Dyslipidemia
rs56289821	19p13.2	2.24E-149	147.69	Dyslipidemia	100%	positive	2.09E-62	Dyslipidemia
rs28399654	19q13.32	1.35E-73	71.91	Dyslipidemia	100%	positive	2.79E-17	Dyslipidemia
rs34095326	19q13.32	9.88E-40	38.04	Dyslipidemia	100%	negative	4.03E-25	Dyslipidemia
rs2927472	19q13.32	2.34E-24	22.67	Dyslipidemia	100%	positive	1.56E-21	Dyslipidemia
rs2230199	19p13.3	9.89E-10	8.04	Macular Degeneration	100%	negative	4.42E-09	Macular Degeneration
rs73015007	19p13.2	3.64E-08	6.48	Dyslipidemia	100%	positive	5.55E-14	Dyslipidemia
rs76186695	19p13.2	1.33E-07	5.91	Dyslipidemia	100%	positive	6.08E-13	Dyslipidemia
rs62211619	20q11.22	1.26E-10	8.94	Cancers	100%	negative	1.32E-10	Cancers
rs2207132	20q12	1.15E-07	5.98	Dyslipidemia	100%	negative	1.99E-07	Dyslipidemia

The chromosome band of a SNP is denoted by 'chrom band'. Direction means whether the SNP is positively or negatively associated with the phenotype. PPA_j denotes the marginal trait-specific posterior probability of association with a risk SNP. E-10 denotes 10^{-10}.
