# Supplementary material for: An efficient Bayesian meta-analysis approach for studying cross-phenotype genetic associations
Source: PLoS Genet. 2018 Feb 12;14(2):e1007139. doi: 10.1371/journal.pgen.1007139 (PMC5825176; doi:10.1371/journal.pgen.1007139)
Supplement: S11 Table — (PDF) [file pgen.1007139.s027.pdf]

S11 Table: Independent pleiotropic signals on chromosome 1-2 detected by ASSET

rsID	chrom band	ASSET p-value	Subset of associated traits selected by ASSET	Univariate p-values	Subset of associated traits selected by $BH_{0.01}$
rs4044578	1q31.3	1.13E-59	Allergic Rhinitis	0.25	Macular-Degeneration
			Cardiovascular Disease	0.47	
			Depressive Disorder	0.54	
			Dermatophytosis	0.26	
			Type 2 Diabetes	0.42	
			Abdominal Hernia	0.14	
			Insomnia	0.58	
			Iron Deficiency	0.49	
			Macular Degeneration	1.16E-63	
			Osteoporosis	0.23	
			Peptic Ulcer	0.63	
			Varicose Veins	0.68	
rs10494745	1q31.3	1.05E-14	Cancers	0.04	Macular-Degeneration
			Dermatophytosis	0.27	
			Type 2 Diabetes	0.13	
			Insomnia	0.03	
			Iron Deficiency	0.28	
			Macular Degeneration	6.15E-21	
			Peripheral Vascular Disease	0.24	
			Peptic Ulcer	0.35	
rs6025	1q24.2	1.38E-11	Dermatophytosis	0.0018	Dermatophytosis
			Hemorrhoids	0.0014	Hemorrhoids
			Iron Deficiency	0.0004	Iron Deficiency
			Osteoporosis	0.0002	Osteoporosis
			Peripheral Vascular Disease	6.81E-14	Peripheral Vascular Disease
rs77394225	1q31.3	6.84E-10	Dermatophytosis	0.62	Macular Degeneration
			Dyslipidemia	0.47	
			Abdominal Hernia	0.65	
			Macular Degeneration	6.04E-16	
			Osteoarthritis	0.30	
			Osteoporosis	0.34	
			Peptic Ulcer	0.46	
			Psychiatric disorders	0.39	
			Stress Disorders	0.32	
rs35505017	1q31.3	3.18E-08	Dermatophytosis	0.56	Macular Degeneration
			Dyslipidemia	0.40	
			Macular Degeneration	3.14E-14	
			Peptic Ulcer	0.63	
			Stress Disorders	0.21	
rs1367117	2p24.1	5.19E-34	Depressive Disorder	0.61	Dyslipidemia
			Dyslipidemia	5.26E-43	
			Hemorrhoids	0.67	
			Abdominal Hernia	0.34	
			Macular Degeneration	0.50	
			Peptic Ulcer	0.57	
			Varicose Veins	0.70	
rs560408	2p24.1	1.17E-18	Cardiovascular Disease	0.23	Dyslipidemia
			Type 2 Diabetes	0.51	
			Dyslipidemia	5.95E-27	
			Abdominal Hernia	0.14	
			Peptic Ulcer	0.05	
			Psychiatric disorders	0.14	
rs79281791	2p24.1	9.75E-10	Dyslipidemia	4.91E-17	Dyslipidemia
			Insomnia	0.007	
			Iron Deficiency	0.04	
			Macular Degeneration	0.17	
			Peptic Ulcer	0.31	
			Varicose Veins	0.13	
rs7601401	2p16.1	1.55E-08	Asthma	0.49	Abdominal-Hernia
			Abdominal Hernia	3.88E-12	Osteoarthritis
			Insomnia	0.24	
			Macular Degeneration	0.34	
			Osteoarthritis	3.46E-06	
			Osteoporosis	0.04	
			Peripheral Vascular Disease	0.59	
			Psychiatric disorders	0.31	
			Stress Disorders	0.27	
			Varicose Veins	0.04	
